# Elucidation of hydrogen bonding networks in theophylline cocrystals by ^1^H-detected ^17^O and ^14^N solid-state NMR spectroscopy

**DOI:** 10.1039/d6cp02064j

**Published:** 2026-09-04

**Authors:** Joseph P. Bequette, Kipper Riemersma, Scott L. Carnahan, Alexander L. Paterson, Aaron J. Rossini

**Affiliations:** a Iowa State University, Department of Chemistry Ames IA 50011 USA arossini@iastate.edu +1 515-294-8952; b National Magnetic Resonance Facility at Madison (NMRFAM), University of Wisconsin-Madison Madison Wisconsin 53706 USA

## Abstract

Determining hydrogen bonding networks and proton positions in pharmaceutical cocrystals remains challenging, particularly for microcrystalline materials and systems involving strong hydrogen bonds and partial proton transfer. Here, we demonstrate a strategy that combines fast magic-angle spinning (MAS) solid-state NMR with ^1^H-detected ^17^O → ^1^H and ^1^H{^14^N} heteronuclear correlation experiments to directly probe hydrogen bonding interactions in theophylline–carboxylic acid cocrystals. Facile ^17^O enrichment of carboxylic acid coformers (benzoic, oxalic, maleic, and malonic acids) was achieved by isotope exchange with ^17^O-enriched water under mild conditions. Isotope exchange reactions can be easily monitored by ^17^O solution NMR spectroscopy. Two-dimensional ^17^O → ^1^H D-RINEPT and ^1^H{^14^N} D-HMQC spectra recorded with variable dipolar recoupling times enable assignment of overlapping ^1^H resonances and provide site-specific identification of intermolecular hydrogen bonds between theophylline and carboxylic acid coformers. High magnetic field SSNMR experiments (up to 25.8 T) further enhance ^17^O spectral resolution and enable resolution of overlapping ^17^O resonances associated with distinct hydroxyl, carbonyl, and dynamically exchanging oxygen environments. Complementary ^1^H spin-diffusion and dipolar double-quantum experiments corroborate intermolecular contacts and confirm cocrystal formation. Experimental ^17^O and ^14^N solid-state NMR parameters are in good agreement with Gauge Including Projector Augmented Wave (GIPAW) density functional theory (DFT) calculations. Together, these results establish a practical multinuclear SSNMR approach for identifying hydrogen bonding networks in pharmaceutical cocrystals and other multicomponent organic solids.

## Abstract 2

^1^H-detected ^17^O and ^14^N solid-state NMR experiments are shown to reveal the hydrogen bonding interactions between components of cocrystals.

## Graphical abstract

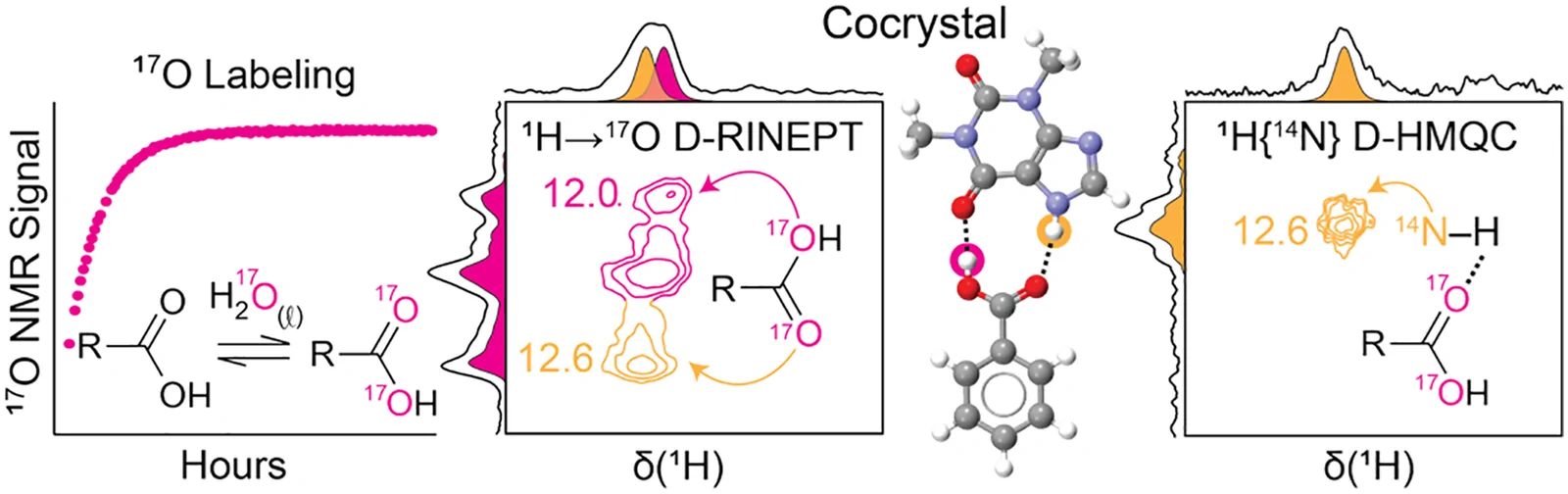

## Introduction

A persistent challenge in pharmaceutical development is the formulation of solid dosage forms for the effective administration of active pharmaceutical ingredients (APIs).^1–5^ Solid APIs may exist in a range of solid forms, including crystalline polymorphs, solvates (most commonly hydrates), salts, cocrystals, and amorphous phases. During solid drug form development, it is essential to identify forms that provide optimal bioavailability and stability while remaining compatible with manufacturing and processing requirements. In addition, the ability to patent novel solid forms provides further incentive for their development and systematic identification.^6–9^ As a result, cocrystallization has emerged over the past several decades as a strategy for expanding the accessible solid-state landscape of pharmaceutical compounds.^10–24^

Pharmaceutical cocrystals are multicomponent crystalline solids composed of an API and a pharmaceutically acceptable coformer in a well-defined stoichiometric ratio that coexist within a single crystal lattice through non-ionic intermolecular interactions.^9,25^ In contrast, cocrystallization of an API with a suitable coformer may also result in the formation of salts, in which proton transfer occurs between acid–base pairs. The likelihood of salt *versus* cocrystal formation is often rationalized using the Δp*K*_a_ rule, where Δp*K*_a_ = p*K*_a_(BH^+^) − p*K*_a_(A). Empirically, Δp*K*_a_ > 4 almost always leads to salt formation, whereas Δp*K*_a_ < −1 almost always leads to cocrystal formation, and intermediate values may yield either phase depending on the crystal environment.^9,26–28^

X-ray diffraction (XRD) is widely used for characterizing pharmaceutical solids,^29,30^ however, it is frequently inapplicable to formulated APIs or amorphous drug forms. Even for crystalline APIs, the growth of diffraction-quality single crystals often presents a significant challenge.^31,32^ Many pharmaceutical cocrystals are obtained through mechanochemical methods, which typically produce microcrystalline powders unsuitable for single-crystal X-ray diffraction.^20,33,34^ In addition, even when X-ray crystal structures are available, the accurate localization of hydrogen atoms, essential for distinguishing salts from cocrystals, can be difficult, owing to the weak X-ray scattering of hydrogen and the resulting ambiguity in proton positions.^35,36^

Due to the limitations of XRD, solid-state nuclear magnetic resonance (SSNMR) spectroscopy has become increasingly important for pharmaceutical solid-form analysis. Numerous studies have demonstrated that chemical shifts, quadrupolar interactions, and heteronuclear correlation experiments provide sensitive, local probes of hydrogen bonding motifs, protonation states, and intermolecular association in multicomponent organic solids.^37–51^ In particular, the development of ^1^H-detected heteronuclear SSNMR experiments with fast magic-angle spinning (MAS) has enabled direct, site-specific interrogation of hydrogen bonding in organic materials. For example, ^1^H-detected ^17^O → ^1^H D-RINEPT experiments or dephasing experiments can be used to establish ^17^O–^1^H dipolar interactions and can be exploited to probe O–H bonding environments and constrain proton positions relative to oxygen atoms.^52–56^ Similarly, ^1^H-detected ^1^H{^14^N} HMQC or dephasing NMR experiments can be used to establish dipolar interactions between ^1^H and ^14^N.^57–60^ The feasibility of recording 2D ^15^N{^17^O} NMR spectra and measuring nitrogen–oxygen inter-atomic distances has also been demonstrated on ^15^N enriched amino acids and peptides.^55,61,62^ There are several recent examples detailing the use of ^1^H{^14^N} solid-state NMR experiments to probe hydrogen bonding and protonation states within multicomponent APIs.^48,49,63^

This work demonstrates that the combination of 2D ^1^H-detected ^14^N and ^17^O SSNMR spectra can be used to directly observe hydrogen bonding interactions between the components of cocrystals. Theophylline, a methylxanthine related to caffeine prescribed for respiratory disorders,^64^ serves as a model API that readily forms cocrystals with pharmaceutically acceptable carboxylic acid coformers benzoic,^65,66^ oxalic,^67^ maleic,^67^ and malonic acids.^67,68^ Notably, we demonstrate facile ^17^O-enrichment of carboxylic acid coformers by isotope exchange with ^17^O-enriched water.^69^ We show that the isotope exchange reactions can easily be monitored by ^17^O solution NMR spectroscopy. 2D ^17^O → ^1^H D-RINEPT and ^1^H{^14^N} D-HMQC experiments are used to assign overlapping ^1^H NMR signals and identify hydrogen bonding interactions between theophylline and carboxylic acid coformers. 2D homonuclear ^1^H spin-diffusion single-quantum and ^1^H dipolar double-quantum single-quantum NMR spectra are shown to further corroborate interactions and substantiate cocrystal purity. Plane-wave density functional theory (DFT) calculations of ^1^H, ^17^O, and ^14^N NMR parameters were also performed to aid spectral assignment and to validate the experimentally determined hydrogen bonding interactions.

## Results and discussion

### Facile ^17^O labeling of carboxylic acids and *in situ* solution ^17^O NMR

**Fig. 1:**
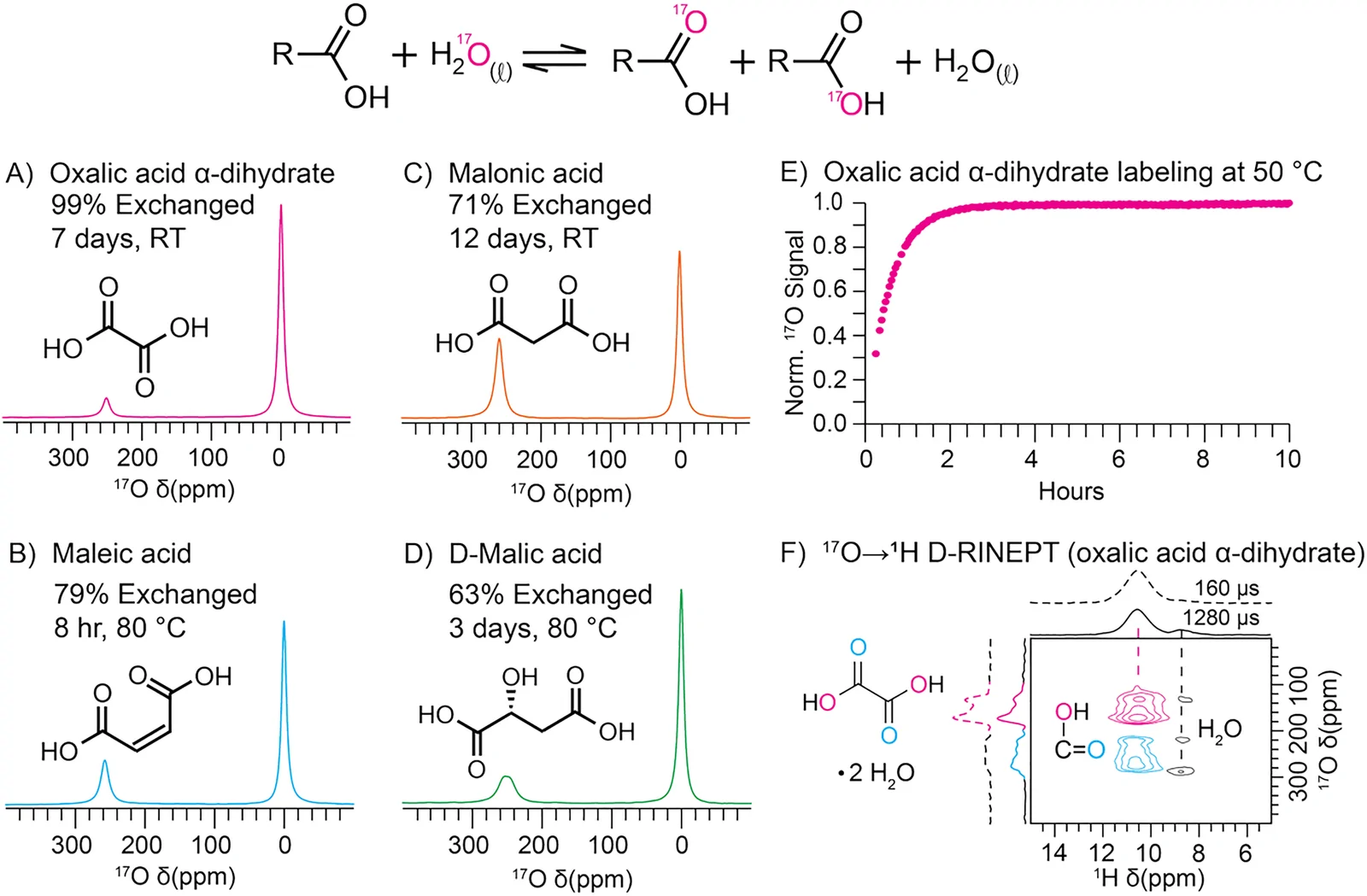
Facile ^17^O labeling of simple carboxylic acids monitored by ^17^O solution NMR spectroscopy. (Top) Scheme illustrating nucleophilic substitution reactions leading to ^17^O isotope exchange in carboxylic acids. (A–D) Solution ^17^O NMR spectra of the indicated carboxylic acids, showing resonances from the carboxylic acid oxygen atoms and water at *ca.* 250 and 0 ppm, respectively. ^17^O-enriched water (3–5%) was used. (E) Time dependence of the oxalic acid ^17^O NMR signal intensity showing that the exchange is complete after *ca.* 5 hours at 50 °C. (F) 2D ^17^O → ^1^H D-RINEPT solid-state NMR spectrum of ^17^O-labeled oxalic acid α-dihydrate acquired with a total recoupling time of 1280 µs at 14.1 T with 50 kHz MAS. Dashed projections correspond to a spectrum acquired with a shorter recoupling time of 160 µs (shown fully in Fig. S1B). The longer recoupling time enhances correlations involving the carbonyl oxygen (C=O), whereas the hydroxyl oxygen is observed at both recoupling times due to its directly attached ^1^H atom.

There are numerous established methods for ^17^O enrichment of carboxylic acids, including acid catalyzed isotope exchange in ^17^O-water and labelling *via* the use of reagents, such as diimides.^70–73^ Most reported isotope exchange conditions for carboxylic acids have focused on using extreme pH with acidification by Cl_2_ or HCl gas, elevated temperatures and long exchange times.^74,75^ To examine the ^17^O-labeling efficiency of dicarboxylic acids, 3–5% ^17^O-labeled water was used to prepare concentrated solutions of oxalic, maleic, malonic, and malic acids. The exchange reactions were monitored by solution ^17^O NMR spectroscopy and the exchange efficiencies were quantified by integration of the acid and water ^17^O NMR signals (Fig. 1A–D). To eliminate effects of limited excitation bandwidth and improve the accuracy of the measured integrals, a ^17^O NMR spectrum was obtained with the transmitter offset on resonance with each peak. Mild heating of the oxalic, maleic, and malic acid solutions afforded isotope exchange efficiencies of approximately 60%–100% within 8 hours (oxalic, maleic) or 3 days (d-malic) of reaction time. Heating the malonic acid solution resulted in decomposition and formation of acetic acid, however a prolonged reaction time of 12 days at room temperature afforded an exchange efficiency of approximately 70%. To more closely follow the exchange progression, time resolved solution ^17^O NMR spectra of a concentrated aqueous oxalic acid solution were obtained that shows the exchange to be complete within a few hours at 50 °C (Fig. 1E). Once the acid concentration and temperature were optimized for each dicarboxylic acid, the exchange was repeated with 39.3% ^17^O water. To obtain the pure solids, the water was removed by heating the solutions uncapped in an 80 °C oven overnight (oxalic, maleic, d-malic) or placed under vacuum (malonic).

In contrast to the dicarboxylic acids, ^17^O-enrichment of benzoic acid required heating at 110 °C for 24 hours in a sealed vial with a catalytic amount of concentrated HCl.^76^ Detailed procedures for ^17^O-enrichment of all carboxylic acids are provided in the SI. The ^17^O-enriched benzoic, oxalic, maleic, and malonic acids were used to synthesize theophylline cocrystals that were subsequently analyzed by ^1^H, ^17^O, and ^14^N solid-state NMR spectroscopy.

### ^1^H and ^17^O SSNMR spectroscopy of oxalic acid α-dihydrate

To demonstrate the feasibility of ^17^O SSNMR experiments on ^17^O labeled carboxylic acids, we acquired the 1D ^17^O spin-echo SSNMR spectrum and 2D ^17^O → ^1^H D-RINEPT spectra of oxalic acid dihydrate (Fig. 1F and Fig. S1). A 1.3 mm rotor and a 50 kHz MAS frequency were used for these experiments to enhance the resolution and sensitivity of the ^1^H SSNMR spectra. All 2D ^1^H–^17^O solid-state NMR spectra were obtained with a dipolar refocused INEPT (D-RINEPT) pulse sequence,^53,77,78^ with *S*R4^2^_1_ dipolar recoupling.^52^ It was previously shown that ^17^O → ^1^H D-RINEPT is an efficient method for acquisition of 2D ^1^H–^17^O heteronuclear correlation solid-state NMR spectra.^53^ The 2D ^17^O → ^1^H D-RINEPT NMR spectrum of oxalic acid dihydrate was obtained with 1280 μs of *S*R4^2^_1_ dipolar recoupling to observe both the carbonyl oxygen (C=O) and hydroxyl oxygen (C–O–H) that are present. The 2D ^17^O → ^1^H D-RINEPT NMR spectrum shows intense correlations between the acid ^1^H NMR signal at 10.6 ppm and both ^17^O NMR signals. Weaker correlations are observed to the ^1^H NMR signals of the lattice waters of hydration. The water ^1^H NMR signals are likely weaker because they exhibit shorter transverse homogeneous relaxation times (*T*_2_′), arising from strong ^1^H–^1^H homonuclear dipolar couplings within the water molecules. Acquisition of the 2D ^17^O → ^1^H D-RINEPT NMR with a shorter recoupling time of 160 µs enhances the relative intensity of the hydroxyl oxygen ^17^O NMR signal (Fig. 1F dashed trace and Fig. S1B). The hydroxyl oxygen has the largest ^1^H–^17^O dipolar coupling constant (*ca.* 13–15 kHz).

### Hydrogen bonding networks in theophylline carboxylic acid cocrystals by ^17^O → ^1^H and ^1^H{^14^N} SSNMR

**Fig. 2:**
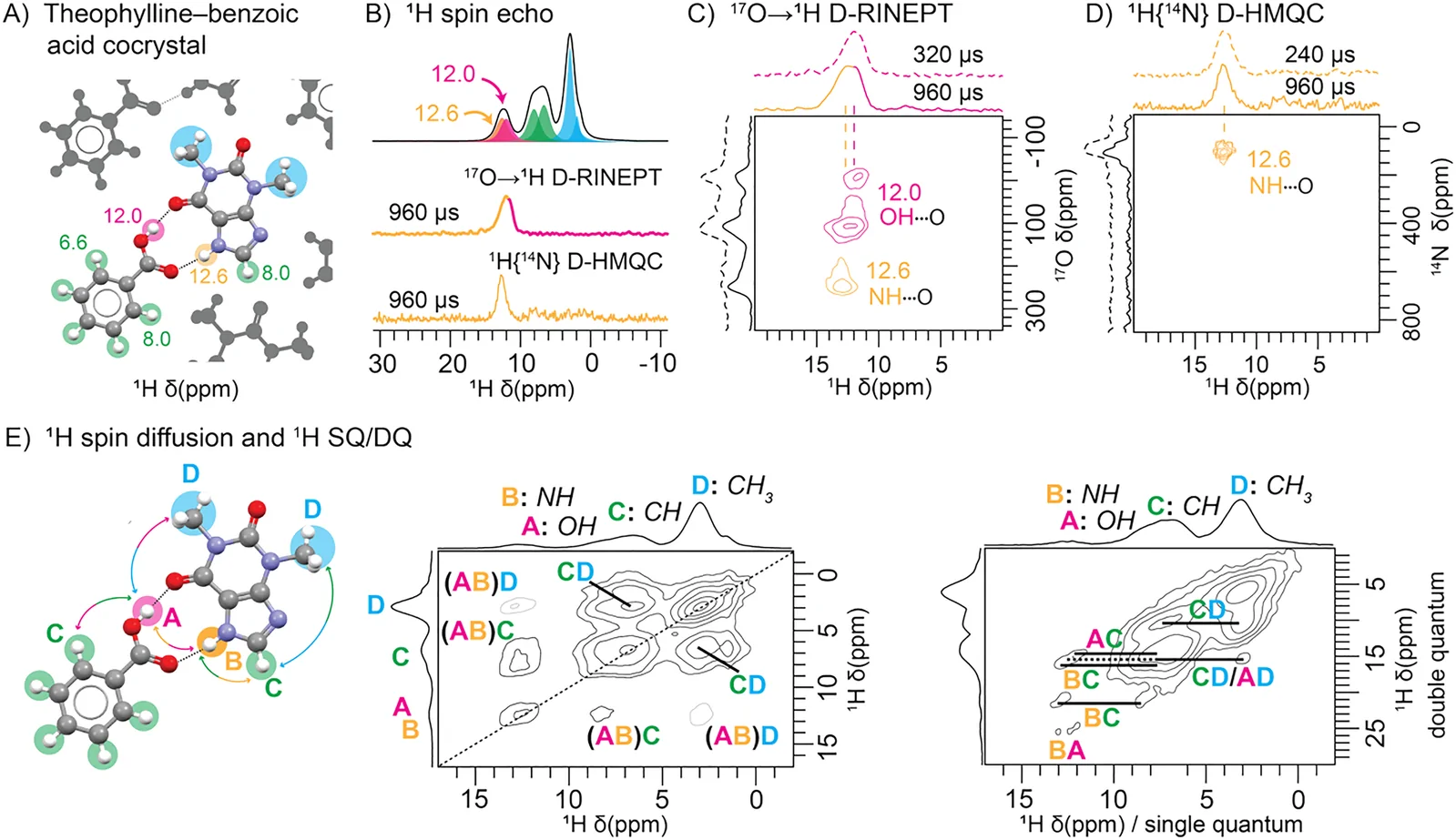
^1^H, ^14^N and ^17^O solid-state NMR experiments on the theophylline–benzoic acid cocrystal. (A) X-ray crystal structure illustrating the hydrogen bonding network between theophylline and benzoic acid with assigned ^1^H chemical shifts. (B) ^1^H solid-state spectrum compared with ^17^O- and ^14^N-filtered ^1^H solid-state NMR spectra. (C) and (D) 2D ^17^O → ^1^H D-RINEPT and ^1^H{^14^N} D-HMQC spectra. Alignment of the ^1^H–^17^O and ^1^H–^14^N correlations, together with comparison of spectra acquired with different recoupling times (dashed traces; full spectra shown in Fig. S3 and S4), enables assignment of the ^1^H, ^17^O, and ^14^N resonances and mapping of the hydrogen bonding network in the cocrystal. (E) ^1^H homonuclear SQ spin-diffusion and ^1^H SQ–DQ correlation spectra with schematic illustrating the corresponding interactions. All NMR experiments were performed with a 9.4 T magnetic field and a 50 kHz MAS frequency. The ^1^H SQ spin diffusion spectrum was acquired with a 200 ms spin diffusion tine, and the ^1^H SQ–DQ spectrum employed BABA recoupling for 1 rotor period for the excitation and reconversion block.

**Fig. 3:**
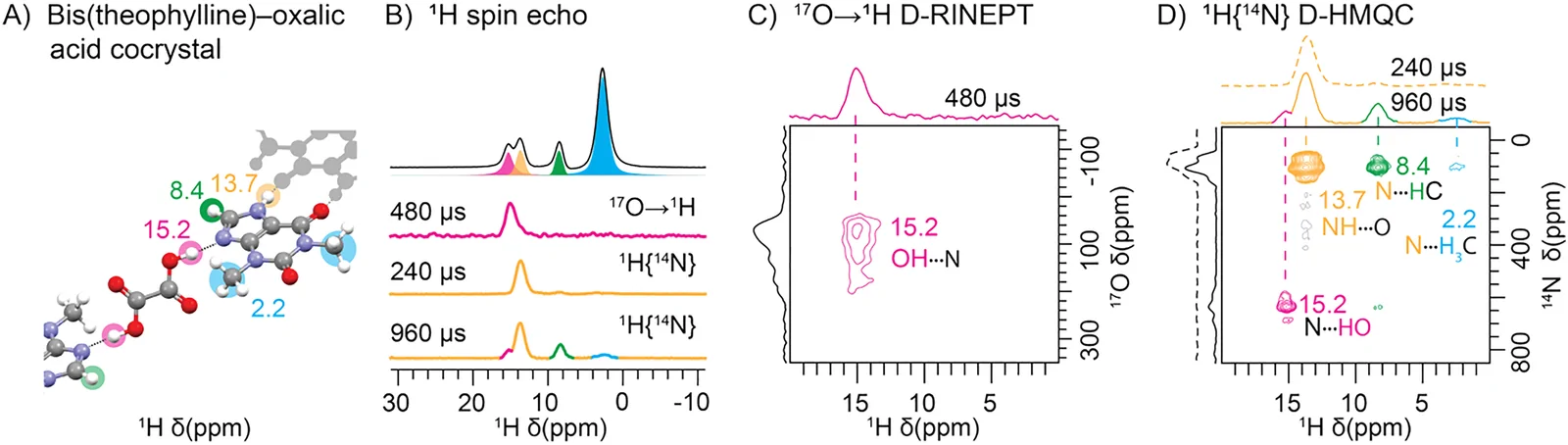
^1^H, ^14^N and ^17^O solid-state NMR experiments on the on the bis(theophylline)-oxalic acid cocrystal. (A) X-ray crystal structure illustrating the hydrogen bonding network between theophylline and oxalic acid with assigned ^1^H chemical shifts. (B) ^1^H solid-state NMR spectrum compared with ^17^O- and ^14^N-filtered ^1^H solid-state NMR spectra. (C) ^17^O → ^1^H D-RINEPT spectrum acquired with a total recoupling time of 480 µs. (D) ^1^H{^14^N} D-HMQC spectrum acquired with a total recoupling time of 960 µs; dashed traces show projections from a spectrum acquired with a shorter recoupling time of 240 µs (full spectrum shown in Fig. S4). All NMR experiments were performed with a 9.4 T magnetic field and a 50 kHz MAS frequency.

**Fig. 4:**
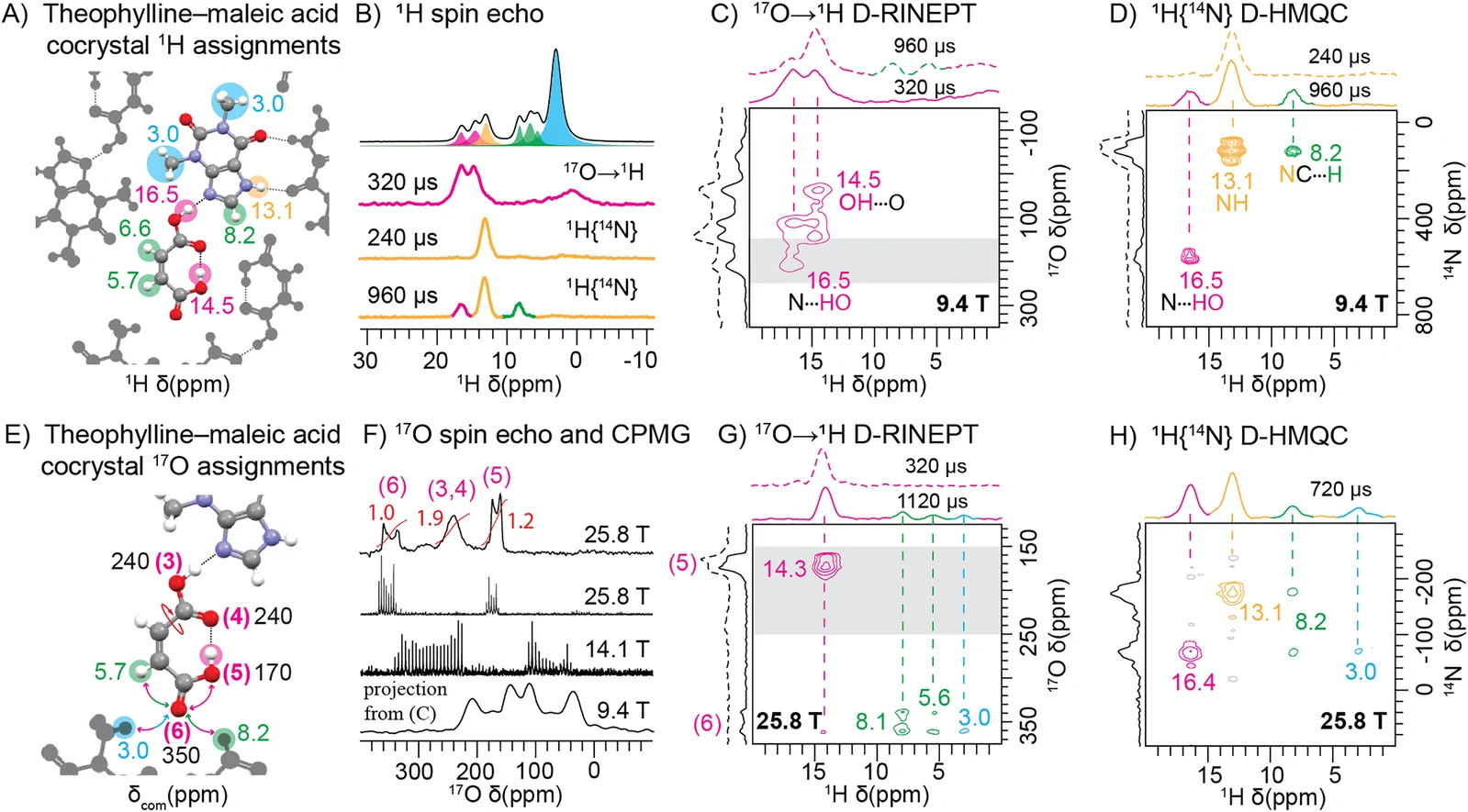
^1^H, ^14^N and ^17^O solid-state NMR experiments on the theophylline–maleic acid cocrystal. (A) X-ray crystal structure illustrating the hydrogen bonding network between theophylline and maleic acid with assigned ^1^H chemical shifts. (B) ^1^H solid-state NMR spectrum compared with ^17^O- and ^14^N-filtered ^1^H solid-state NMR spectra. (C) ^17^O → ^1^H D-RINEPT spectrum acquired with a total recoupling time of 320 µs; dashed traces show projections from a spectrum acquired with a longer recoupling time of 960 µs (full spectrum shown in Fig. S3B). (D) ^1^H{^14^N} D-HMQC spectrum acquired with a total recoupling time of 960 µs; dashed traces show projections from a spectrum acquired with a shorter recoupling time of 240 µs (full spectrum shown in Fig. S4C). Experiments were performed with a 9.4 T magnetic field and a 50 kHz MAS frequency. (E) X-ray crystal structure with ^17^O center-of-mass shift (*δ*_com_) assignments. Values in parentheses denote oxygen numbering; oxygen atoms 3 and 4 undergo fast exchange *via* rotation around the C–C bond axis. (F) MAS ^17^O spin-echo and QCPMG NMR spectra recorded at magnetic field strengths of 25.8 T, 14.1 T, and 9.4 T, as indicated. The top spectrum (25.8 T) was integrated to determine the relative populations of the three distinct oxygen environments, consistent with the assignments shown. (G) ^17^O → ^1^H D-RINEPT spectrum recorded at 25.8 T acquired with a total recoupling time of 1120 µs; dashed traces show projections from a spectrum acquired with a shorter recoupling time of 320 µs (full spectrum shown in Fig. S3D). (H) ^1^H{^14^N} D-HMQC spectrum recorded at 25.8 T with a total recoupling time of 720 µs. Unless otherwise noted, experiments in panels F–H were performed with a 25.8 T magnetic field and a 50 kHz MAS frequency.

**Fig. 5:**
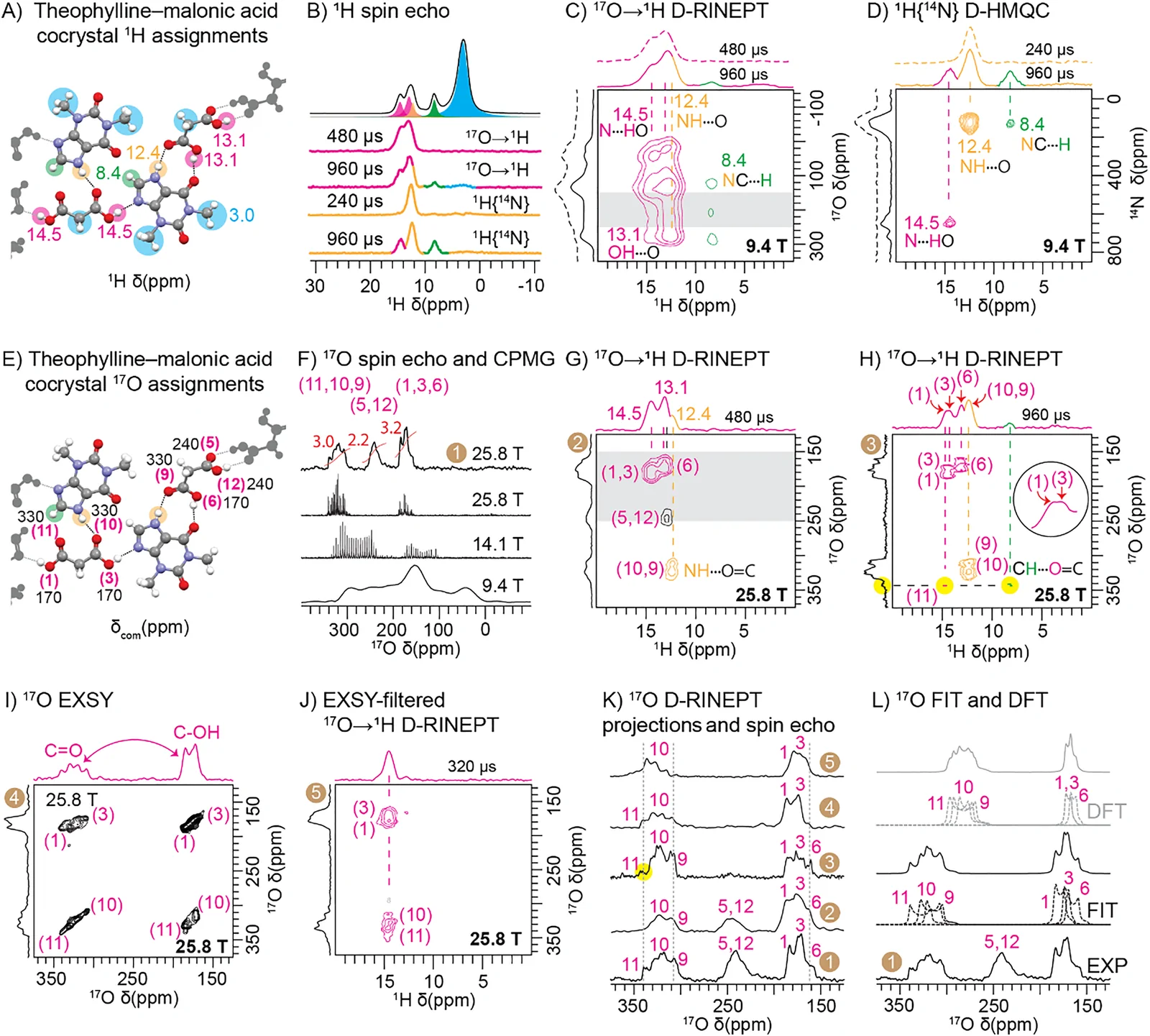
^1^H, ^14^N and ^17^O solid-state NMR experiments on the theophylline–malonic acid cocrystal. (A) X-ray crystal structure illustrating the hydrogen bonding network between theophylline and malonic acid with assigned ^1^H chemical shifts. (B) ^1^H solid-state NMR spectrum compared with ^17^O- and ^14^N-filtered ^1^H solid-state NMR spectra. (C) ^17^O → ^1^H D-RINEPT spectrum acquired with a total recoupling time of 960 µs; dashed traces show projections from a spectrum acquired with a shorter recoupling time of 480 µs (full spectrum shown in Fig. S3C). (D) ^1^H{^14^N} D-HMQC spectrum acquired with a total recoupling time of 960 µs; dashed traces show projections from a spectrum acquired with a shorter recoupling time of 240 µs (full spectrum shown in Fig. S4D). Experiments in panels B–D were performed with a 9.4 T magnetic field and a 50 kHz MAS frequency. (E) X-ray crystal structure with ^17^O center-of-mass shift (*δ*_com_) assignments. Values in parentheses denote oxygen numbering; oxygen atoms 5 and 12 undergo tautomerization (Fig. S9) and therefore give rise to the same resonance at 240 ppm. (F) MAS ^17^O spin-echo and QCPMG NMR spectra recorded at magnetic field strengths of 25.8 T, 14.1 T, and 9.4 T as indicated. The top spectrum (25.8 T) was integrated to determine the relative populations of the three distinct oxygen environments, consistent with the assignments shown. (G) ^17^O → ^1^H D-RINEPT spectrum recorded at 25.8 T with a total recoupling time of 480 µs. (H) ^17^O → ^1^H D-RINEPT spectrum recorded at 25.8 T with a total recoupling time of 960 µs. (I) 2D ^17^O EXSY spectrum recorded at 25.8 T with an exchange time of 100 ms. Oxygen atom pairs 11/1 and 3/10 undergo slow exchange *via* 180° rotations around the C-C bonds. (J) EXSY-filtered ^17^O → ^1^H D-RINEPT spectrum recorded at 25.8 T with a total recoupling time of 320 µs. (K) Comparison of ^17^O spectra from the spin-echo experiment in panel F (top) with D-RINEPT projections from panels G–J. (L) Analytical simulations of ^17^O spin-echo NMR spectra and simulated spectra using DFT-calculated parameters. Unless otherwise noted, experiments in panels F–L were performed with a 25.8 T magnetic field and a 50 kHz MAS frequency. Complementary ^1^H{^14^N} D-HMQC spectra recorded at 25.8 T with total recoupling times of 240 μs and 720 µs are shown in Fig. S4E and F.

To directly elucidate the hydrogen bonding networks and intermolecular interactions in theophylline-carboxylic acid cocrystals, we performed 1D and 2D ^1^H solid-state NMR experiments, combined with 2D ^17^O → ^1^H and ^1^H{^14^N} heteronuclear correlation experiments. We have obtained solid-state NMR spectra of theophylline cocrystals of benzoic, oxalic, maleic, and malonic acid (Fig. 2–5). All theophylline cocrystals were synthesized by solution phase reactions of theophylline with the desired carboxylic acid coformer. Powder X-ray diffraction (PXRD) was used to confirm the successful synthesis of the cocrystals with the desired crystal structures (Fig. S2A–F). All solid-state NMR experiments were performed with a MAS frequency of 50 kHz. Solid-state NMR experiments were primarily performed with a magnetic field of 9.4 T since this magnet was readily available for experiments. However, we also performed some NMR experiments at magnetic fields of 14.1 T and 25.8 T.

### Solid-state NMR experiments on the theophylline–benzoic acid cocrystal

We start with a description of NMR experiments on the theophylline–benzoic acid cocrystal first because this is the simplest system. From ^17^O solution NMR experiments the ^17^O enrichment of the benzoic acid oxygen atoms was estimated to be 20%. The previously published single-crystal X-ray diffraction structure shows that theophylline–benzoic acid dimers are connected *via* two hydrogen bonding interactions: (1) between the carboxylic acid proton (OH) of benzoic acid and the carbonyl oxygen (C=O) of theophylline and (2) between the imidazole nitrogen (NH) of theophylline and the carbonyl oxygen (C=O) of benzoic acid (Fig. 2A).

The 50 kHz MAS ^1^H spin-echo NMR spectrum of the theophylline–benzoic acid cocrystal is shown in Fig. 2B, followed by the 2D ^1^H–^17^O and ^1^H–^14^N dipolar-correlation NMR spectra (Fig. 2C and D). All 2D ^1^H–^14^N solid-state NMR spectra were obtained with a ^1^H{^14^N} D-HMQC pulse sequence^77,79^ with the *S*R4^2^_1_ symmetry-based pulse sequence used for dipolar recoupling.^52^^14^N excitation pulses were one rotor cycle in duration (20 µs).^79^ These pulses were observed to be more efficient than using short duration excitation pulses for the systems studied here. The optimal ^14^N pulse duration depends on the quadrupolar product (*P*_*Q*_) of the ^14^N site, and therefore may vary between different nitrogen environments. Notably, a broad high frequency ^1^H NMR signal centered at 12.6 ppm is observed in the ^1^H spin-echo NMR spectrum. Partial overlap of the carboxylic acid ^1^H NMR signal (pink trace) and the imidazole NH ^1^H NMR signal (orange trace) occurs in the ^1^H NMR spectrum. The 2D ^17^O → ^1^H D-RINEPT spectrum of the theophylline–benzoic acid cocrystal obtained with a 960 µs total duration of *S*R4^2^_1_ dipolar recoupling shows correlations between the ^17^O NMR signal of the protonated O of the carboxylic acid (C–OH) to a ^1^H chemical shift of 12.0 ppm. This ^1^H chemical shift corresponds to the carboxylic acid proton, which was separately confirmed by acquiring a ^17^O → ^1^H D-RINEPT with a shorter total recoupling time of 320 µs (dashed traces and Fig. S3). The non-protonated carbonyl oxygen (C=O) of the carboxylic acid shows correlations to a ^1^H NMR signal with a chemical shift of 12.6 ppm. The ^1^H NMR signal at 12.6 ppm corresponds to the theophylline NH within the imidazole ring, which was confirmed by the 2D ^1^H{^14^N} D-HMQC spectrum (Fig. 2D and Fig. S4). Therefore, from comparison of the 2D ^1^H–^17^O and ^1^H–^14^N NMR spectra, it is immediately apparent that there is a hydrogen bond between the benzoic acid carbonyl oxygen (C=O) and the theophylline imidazole (NH). The absence of longer-range H–O correlations for the carbonyl carboxylic acid proton (OH) suggests that it must be hydrogen bonded to a spin-inactive atom, in this case, the nearby carbonyl oxygen (C=O) of theophylline which has natural ^17^O abundance.

The ^17^O → ^1^H D-RINEPT and ^1^H{^14^N} D-HMQC experiments offer reasonable sensitivity, enabling acquisition of the 2D NMR spectra in Fig. 2C and D within approximately 10 hours and 4 hours, respectively. To further probe intermolecular contacts, 2D ^1^H–^1^H single-quantum–single quantum (SQ–SQ) spin-diffusion and ^1^H–^1^H single-quantum–double quantum (SQ–DQ) correlation experiments were performed (Fig. 2E). These experiments provide complementary information on proton proximities within and between theophylline and benzoic acid molecules. In the ^1^H spin-diffusion spectrum acquired with a mixing time of 200 ms, cross peaks are observed between several proton sites; however, overlap of the benzoic acid hydroxyl proton (12.0 ppm, site A) and the theophylline imidazole NH proton (12.6 ppm, site B) prevents unambiguous differentiation of their correlations. In contrast, the ^1^H SQ–DQ spectrum resolves a clear correlation between these two sites (B–A), directly indicating spatial proximity between the benzoic acid OH and the theophylline NH groups. Several additional correlations are observed between the benzoic acid hydroxyl proton and the other ^1^H atoms of benzoic acid the theophylline.

### Solid-state NMR experiments on the theophylline–oxalic acid cocrystal

Theophylline dicarboxylic acid cocrystals of oxalic, maleic, and malonic acid are extended (open) hydrogen bonded networks and unlike the benzoic acid cocrystal which consists of closed dimeric synthons, the carboxylic acid ^1^H NMR signals all appear at higher frequencies than the theophylline imidazole N–H proton signal. In the bis(theophylline)–oxalic acid cocrystal, an oxalic acid molecule is sandwiched between two theophylline molecules and forms symmetric OH⋯N hydrogen bonds, in which each oxalic acid hydroxyl group forms a hydrogen bond to the imine (pyridine-like) nitrogen of a theophylline molecule (Fig. 3A). The ^1^H NMR spectrum, together with complementary 2D ^17^O → ^1^H D-RINEPT and ^1^H{^14^N} D-HMQC spectra, shows that the hydrogen bonded carboxylic acid proton resonates at 15.2 ppm, while the theophylline imidazole N–H proton, which is hydrogen bonded to a non-enriched theophylline carbonyl oxygen, appears at 13.7 ppm (Fig. 3B–D). The ^1^H{^14^N} D-HMQC spectrum additionally reveals weaker correlations between the imine nitrogen of theophylline and the ^1^H resonances at 8.4 ppm and 2.2 ppm, assigned to the theophylline CH and CH_3_ protons, respectively (Fig. 3D). The ^17^O solid-state NMR spectra of this cocrystal extracted from a directly detected spin echo and from the 2D ^17^O → ^1^H D-RINEPT show a single broad resonance at 9.4 T (Fig. S5). The position of this resonance is intermediate between that expected for a carbonyl oxygen (C=O) and hydroxyl oxygen (C–OH), suggesting that chemical exchange of these two oxygen atoms is intermediate or fast on the NMR time scale.^80^ Wu and co-workers have previously observed dynamic 180° rotations of carboxylic acid groups in nicotinic acid by 1D variable temperature ^17^O solid-state NMR spectroscopy and ^17^O EXSY experiments.^80^ As is shown below, several of the carboxylic acids groups in other cocrystals studied here also undergo similar dynamic reorientations. Acquisition of the ^17^O solid-state NMR spectrum of the oxalic acid cocrystal at a higher magnetic field of 14.1 T results in two well-defined resonances for the carbonyl and hydroxyl oxygen (Fig. S5), likely due to increased frequency separation of the two peaks which results in slow exchange in the NMR time scale. ^1^H–^1^H SQ–SQ spin-diffusion and ^1^H–^1^H SQ–DQ NMR spectra are shown in Fig. S6 and S7.

### Solid-state NMR experiments on the theophylline–maleic acid cocrystal

The single-crystal X-ray structure of theophylline–maleic acid cocrystal shows the two carboxylic acid groups of maleic acid are non-equivalent (Fig. 4A). One of the carboxylic acid protons hydrogen bonds to the imine nitrogen of theophylline as stated above, while the other forms an intramolecular hydrogen bond. Analysis of the ^1^H NMR spectrum and complementary 2D ^17^O → ^1^H D-RINEPT and ^1^H{^14^N} D-HMQC NMR spectra shows the intermolecular H-bonded carboxylic acid proton appears at 16.5 ppm, followed by the intramolecular H-bonded carboxylic acid proton at 14.5 ppm and the theophylline imidazole NH proton at 13.1 ppm (Fig. 4B–D). The ^1^H{^14^N} D-HMQC NMR spectrum shows an additional weaker correlation between the imidazole NH nitrogen of theophylline and the ^1^H signal at 8.2 ppm attributed to the CH hydrogen of theophylline. The remaining non-methyl signals at 5.7 ppm and 6.6 ppm were assigned by comparison to plane-wave GIPAW DFT calculations of predicted ^1^H chemical shifts (Table S1), consistent with previous studies demonstrating the accuracy of GIPAW calculations for predicting ^1^H chemical shifts in molecular solids.^81^

To improve ^17^O NMR resolution, we repeated 1D and 2D NMR experiment at magnetic fields of 14.1 T and 25.8 T. Fig. 4E shows the theophylline–maleic acid cocrystal crystal structure annotated with ^17^O center-of-mass shifts. At 9.4 T the spectrum consists of broad overlapping MAS quadrupolar powder patterns. At 25.8 T the improved resolution separates the spectrum into three distinct regions centered near 170 ppm, 240 ppm, and 350 ppm. The hydroxyl oxygen (5) appears at the lowest frequency (∼170 ppm), the oxygen pair undergoing dynamic exchange (3,4) appears at ∼240 ppm, and the carbonyl oxygen (6) appears at ∼350 ppm. Integrals are shown for the 1D ^17^O NMR spectrum obtained at 25.8 T (Fig. 4F and Fig. S8). Integration of the 25.8 T spin-echo ^17^O MAS NMR spectrum confirms the relative populations of the three oxygen environments, consistent with the assignments shown in Fig. 4E. The signal at ∼240 ppm exhibits a broad lineshape consistent with dynamic exchange between oxygen sites (3) and (4), indicative of 180° rotations around the C–C bond axis. If exchange is fast on the NMR time scale between the C=O and C–OH oxygen atoms, we expect that the NMR signal will appear at an averaged shift, explaining why there is a ^17^O NMR signal centered at 240 ppm. This exchange process results in a short homogeneous transverse relaxation time constant (*T*_2_′) for oxygens 3 and 4, explaining why these NMR signals are absent from QCPMG spectra recorded at 25.8 T and 14.1 T. Oxygen atom (5) corresponds to the hydroxyl oxygen involved in the intramolecular hydrogen bond and resonates near 170 ppm. The remaining oxygen atom (6) appears near 350 ppm and shows correlations to protons at 14.5, 8.2, 5.7, and 3.0 ppm, as established from the experiments described in Fig. 4F–H.

Temperature-dependent ^17^O NMR experiments further support the interpretation given above. The 25.8 T spin-echo spectrum shown in Fig. 4F was recorded with an estimated sample temperature of approximately 51 °C. Comparison of ^17^O solid-state NMR spectra acquired with estimated sample temperatures of approximately 40 °C and −5 °C show that the resonance corresponding to oxygen atoms (3,4) disappears at lower temperature (Fig. S8), consistent with an intermediate exchange regime at lower temperatures that broadens the ^17^O NMR signal beyond detection. Further details on the variable temperature experiments are provided in the SI.

Fig. 4G shows the ^17^O → ^1^H D-RINEPT spectrum recorded at 25.8 T with a total recoupling time of 1120 µs together with projections from a spectrum recorded with a shorter 320 µs recoupling time (full spectrum shown in Fig. S3D). The longer recoupling time experiment shows an intense correlation at 14.3 ppm corresponding to the hydroxyl proton assigned at 14.5 ppm in Fig. 4A. The small difference in chemical shift likely arises from the different temperatures used in the measurements (Fig. 4A was recorded at *ca.* 40 °C, whereas the spectrum in Fig. 4G was acquired at *ca.* 51 °C). This proton is directly bonded to the oxygen that resonates near 170 ppm in the ^17^O dimension. Additional weaker correlations at 8.1 ppm, 5.6 ppm, and 3.0 ppm correlate with the ^17^O resonance near 350 ppm, indicating spatial proximity of oxygen (6) to the theophylline CH and methyl protons. In particular, the weak correlation to the 5.7 ppm proton supports the structural assignment predicted from DFT calculations. The corresponding ^17^O → ^1^H INEPT correlation for the 16.5 ppm hydroxyl proton is absent from the 2D spectrum because the ^17^O coherence undergoes significant homogeneous transverse relaxation during the initial INEPT transfer period, attenuating the signal.

Finally, Fig. 4H shows the ^1^H{^14^N} D-HMQC spectrum recorded at 25.8 T with a total recoupling time of 720 µs. The ^14^N NMR signals should be narrowed at 25.8 T as compared to 9.4 T due to a reduction in second- and third-order quadrupolar interactions at higher field, however, it was difficult to accurately set the magic angle on the probe used for the 25.8 T NMR experiments. Two nitrogen environments are observed at approximately −170 ppm and −70 ppm. The signal near −170 ppm correlates with protons at 13.1 ppm and 8.2 ppm, corresponding to the theophylline imidazole NH and adjacent CH proton. The nitrogen resonance near −70 ppm correlates with protons at 16.4 ppm and 3.0 ppm. The 16.4 ppm correlation arises from the maleic acid hydroxyl proton that hydrogen bonds to the theophylline imine nitrogen, while the 3.0 ppm correlation reflects the spatial proximity of this nitrogen to the theophylline methyl groups.

### Solid-state NMR experiments on the theophylline-malonic acid cocrystal

The theophylline–malonic acid cocrystal represents the most structurally complex system examined here because the asymmetric unit contains two symmetry-independent pairs (*Z*′ = 2). Consequently, the crystal structure contains four crystallographically distinct carboxylic acid hydroxyl groups (Fig. 5A). Despite this structural complexity, the ^1^H spin-echo spectrum shows only two dominant hydroxyl proton resonances at 13.1 ppm and 14.5 ppm (Fig. 5B–D). Analysis of the ^17^O → ^1^H D-RINEPT and ^1^H{^14^N} D-HMQC NMR spectra indicates that the 14.5 ppm signal corresponds to a carboxylic acid OH hydrogen bonded to the imine nitrogen of theophylline, whereas the 13.1 ppm signal corresponds to a carboxylic acid OH hydrogen bonded to a carbonyl oxygen atom of either theophylline or another malonic acid molecule. A strong ^17^O → ^1^H correlation at 12.4 ppm additionally indicates hydrogen bonding between a malonic acid carbonyl oxygen and the theophylline NH group.

Fig. 5E summarizes the ^17^O center-of-mass shift assignments. ^17^O spin-echo and CPMG NMR spectra recorded at magnetic field strengths of 25.8 T, 14.1 T, and 9.4 T are shown in Fig. 5F. At 9.4 T the spectrum appears as a broad unresolved feature due to extensive overlap of the oxygen resonances, whereas at 25.8 T three distinct regions are resolved near 170 ppm, 240 ppm, and 330 ppm. Integration of the 25.8 T spectrum confirms the relative populations of the hydroxyl, exchanging, and carbonyl environments. Oxygen atoms (5) and (12) participate in a carboxylic acid dimer and undergo tautomerization between hydroxyl and carbonyl configurations (Fig. S9). As a result, these sites share a single averaged ^17^O resonance near 240 ppm resulting from fast chemical exchange. The resonance at ∼240 ppm exhibits a broad lineshape and is absent in the CPMG NMR spectra recorded at 25.8 T and 14.1 T, consistent with a short *T*_*2*_ arising from the tautomerization process. This resonance remains clearly visible in the spin-echo spectrum even at −10.5 °C, indicating that tautomerization between oxygen sites (5) and (12) remains rapid on the NMR time scale (Fig. S9). The remaining carbonyl oxygen atoms appear near 330 ppm, while the hydroxyl oxygen atoms appear near 170 ppm.

Additional structural insight is obtained from ^17^O → ^1^H D-RINEPT spectra recorded at 25.8 T (Fig. 5G and H). With a total recoupling time of 480 µs, correlations involving the exchanging oxygen sites (5,12) are observed, whereas these correlations disappear at longer recoupling times. In the longer-recoupling spectrum the 14.5 ppm resonance resolves into two closely spaced peaks assigned to hydroxyl oxygen atoms (1) and (3) (Fig. 5H inset). The hydroxyl oxygen atom (1) shows a correlation with carbonyl oxygen (11), which belongs to the same carboxylic acid group and is the only carbonyl oxygen not directly involved in hydrogen bonding. In contrast, carbonyl oxygen atoms (9) and (10) show correlations with the theophylline imidazole NH proton at 12.4 ppm.

Dynamic exchange between oxygen sites is directly observed in the 25.8 T ^17^O EXSY NMR spectrum shown in Fig. 5I. With a mixing time of 100 ms, exchange cross-peaks are observed between hydroxyl oxygen atoms (1) and (3) with carbonyl oxygen atoms (11) and (10), respectively. The EXSY-filtered ^17^O → ^1^H D-RINEPT spectrum (Fig. 5J) confirms that these oxygen sites all correlate with the proton resonance at 14.5 ppm.

Although ^17^O–^17^O dipolar-mediated spin exchange could, in principle, also generate cross-peaks in an EXSY experiment, we believe this contribution is minor. The ^17^O enrichment of the carboxylic acid coformers is estimated to be below 20%, such that only a fraction of carboxyl groups contain two ^17^O nuclei. Furthermore, the observed correlations occur between hydroxyl and carbonyl oxygen environments that are expected to interconvert through proton transfer or carboxyl-group reorientation. Additionally, the fast MAS frequencies of 50 kHz and the large frequency separation (*ca.* 20 kHz) of the ^17^O NMR signals will also inhibit ^17^O spin exchange. Therefore, we attribute the observed cross-peaks primarily to chemical exchange, while recognizing that a minor contribution from ^17^O–^17^O dipolar-mediated spin exchange cannot be completely excluded.

Comparison of the ^17^O spin-echo NMR spectrum with projections from the correlation experiments (Fig. 5K) allows individual oxygen sites to be assigned within the carbonyl (∼330 ppm) and hydroxyl (∼170 ppm) regions. Finally, the 25.8 T ^17^O spectrum was analytically simulated and compared with plane-wave DFT calculations (Fig. 5L). The resulting shifts and quadrupolar parameters agree well with calculated values (Table S2), enabling assignment of carbonyl oxygen atoms (11), (10), and (9) and hydroxyl oxygen atoms (1), (3), and (6) from left to right in their respective spectral regions. Together, the ^17^O → ^1^H and ^1^H{^14^N} correlation experiments, EXSY measurements, high-field ^17^O NMR spectra provide a comprehensive description of the hydrogen bonding interactions and dynamic tautomerization processes present in the theophylline–malonic acid cocrystal.

### Plane-wave DFT calculations of ^1^H, ^14^N and ^17^O NMR observables

**Fig. 6:**
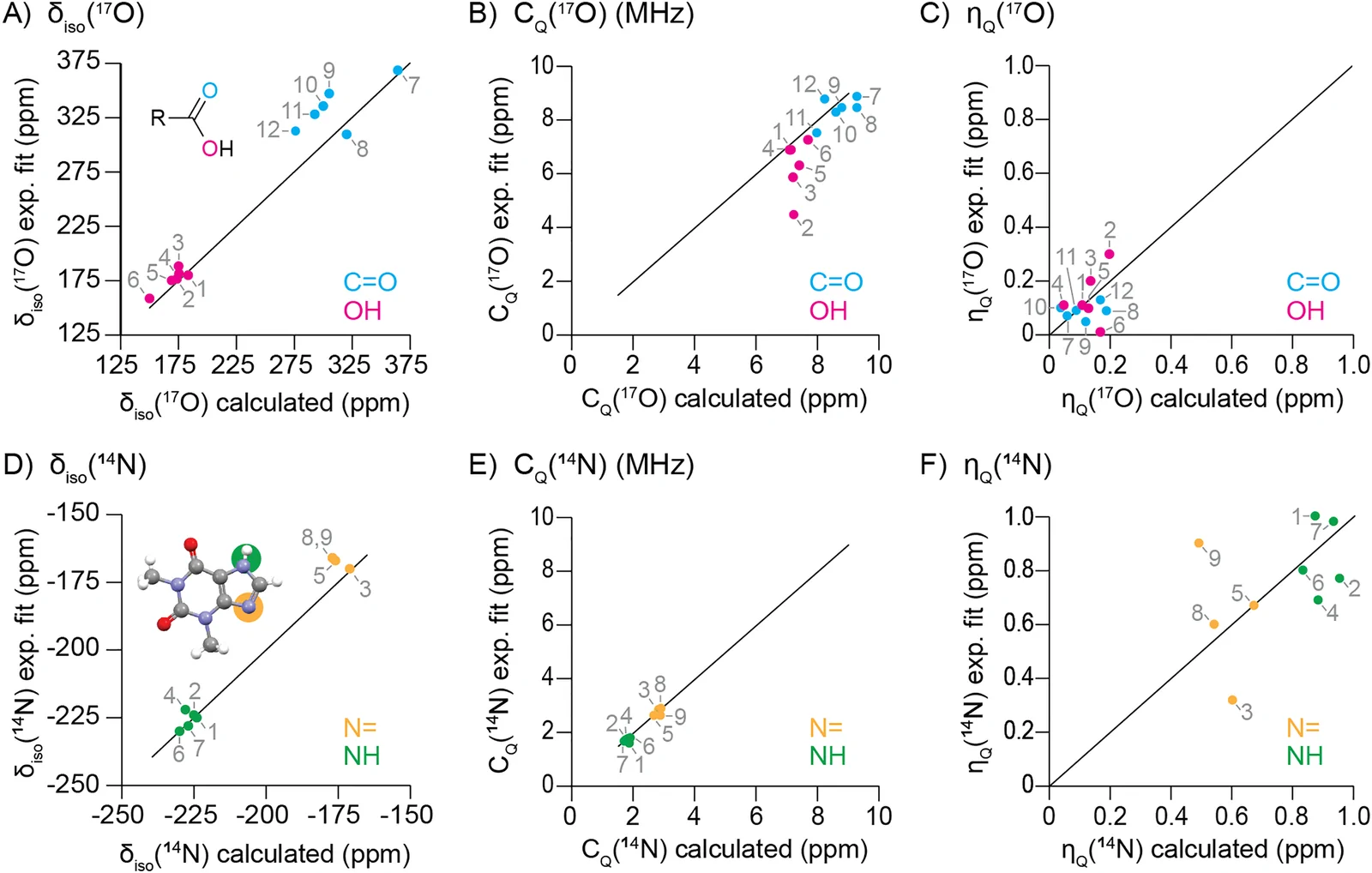
Comparison of experimental fit (exp. fit) and DFT calculated parameters for theophylline cocrystals. Diagonal lines are guides for the eye that indicate identical experimental and calculated parameters. (A) Isotropic chemical shift (*δ*_iso_) for ^17^O NMR spectra. (B) Quadrupolar coupling constant (*C*_Q_) for ^17^O NMR spectra. (C) EFG tensor asymmetry parameter (*η*_Q_) for ^17^O NMR. (D) Isotropic ^14^N chemical shifts. (E) *C*_Q_ for ^14^N NMR spectra. (F) *η*_Q_ for ^14^N NMR. ^17^O NMR spectra: (1) maleic OH, (2,3,5) malonic OH, (4) oxalic OH, (6) benzoic OH, (7) maleic C=O, (8) oxalic C=O, (9,10,11) malonic OH, (12) benzoic C=O. ^14^N NMR spectra: (1) benzoic NH, (2) oxalic NH, (3) oxalic N=, (4) maleic NH, (5) maleic N=, (6) malonic NH1, (7) malonic NH2, (8) malonic N1=, (9) malonic N2=. *C*_Q_ values are plotted as absolute values. DFT calculated parameters are provided in Table 1 and Tables S1, and S3–S5. Experimental fit parameters are provided in Tables S3–S5. Oxygen sites in maleic and malonic acid cocrystals undergoing dynamic exchange are omitted.

Experimental ^17^O CPMG NMR spectra acquired at 14.1 T with 40 kHz MAS frequencies and projections from 2D ^1^H{^14^N} D-HMQC spectra acquired at 9.4 T with 50 kHz MAS frequencies were fit (Fig. S10) and compared to parameters obtained by plane-wave DFT calculations (Fig. 6 and Table 1, and Tables S1, S4, and S5). ^17^O NMR spectra were fit with two sites (OH at *ca.* 175 ppm and C=O at *ca.* 300 ppm) and the corresponding DFT calculated values for the maleic and malonic acid cocrystals (which contain multiple OH and C=O signals) were averaged. Calibration curves from previous reports^53,82^ were used to convert ^17^O and ^14^N chemical shielding values to shifts. EFG tensor parameters *C*_Q_ and *η*_Q_ for ^17^O spectra were also corrected using the calibration curves. A comparison of the experimental and DFT calculated NMR parameters is summarized in Table 1. In general, the DFT calculated parameters are in good agreement with experimental results (Fig. 6).

**Table 1:** Comparison of experimental and DFT calculated NMR parameters for hydrogen bonded sites in the theophylline–cocrystals

Cocrystal	Site	Experimental			DFT calculated			|Absolute difference|^d^		
		*δ* a	*C*_Q_ b,	*η*_Q_ c	*δ* a	|*C*_Q_^b^|,	*η*_Q_ c	*δ* a	*C*_Q_ b,	*η*_Q_ c
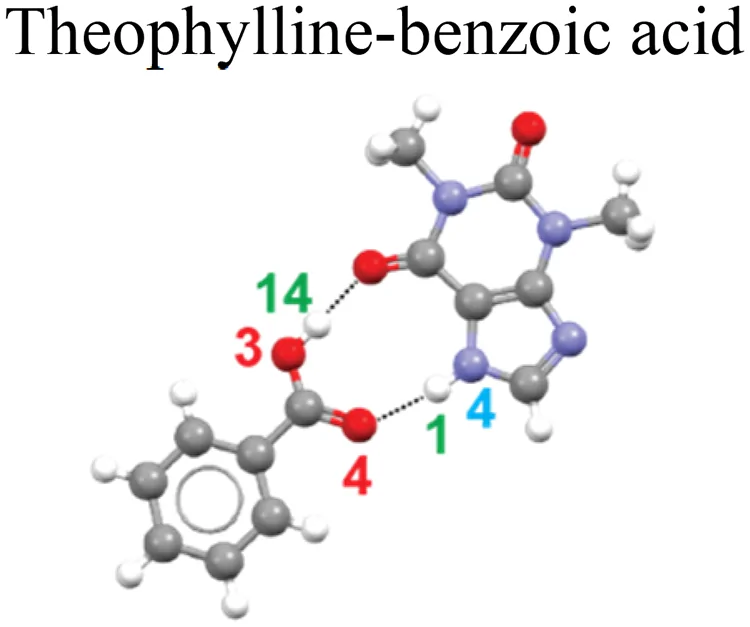	H-1	12.6			13.0			0.4		
	H-14	12.0			12.0			0		
	O-3	158	7.3	0.01	150	7.7	0.17	8	0.4	0.16
	O-4	313	8.8	0.13	276	8.2	0.17	37	0.6	0.04
	N-4	–225	1.6	1.00	–224	1.8	0.87	1	0.2	0.13
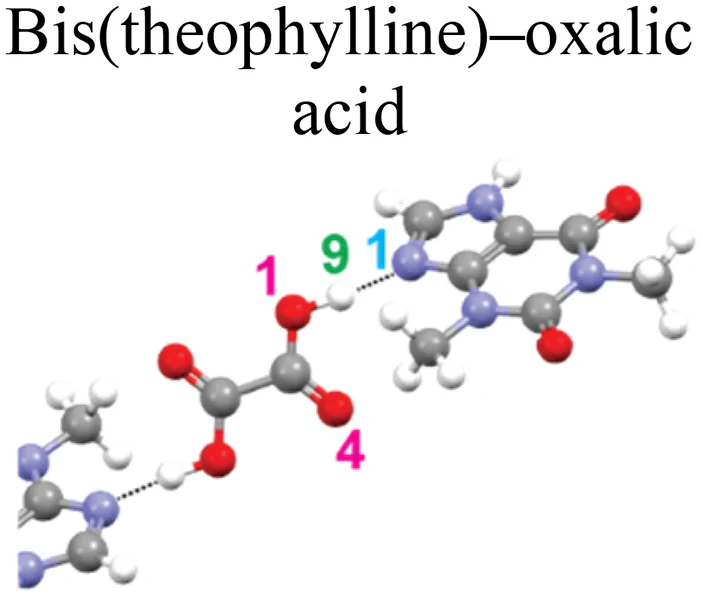	H-9	15.2			14.3			0.9		
	O-1	180	6.9	0.11	175	7.1	0.05	5	0.2	0.06
	O-4	310	8.5	0.09	320	9.3	0.19	10	0.8	0.1
	N-1	–170	2.9	0.32	–171	2.8	0.60	1	0.1	0.28
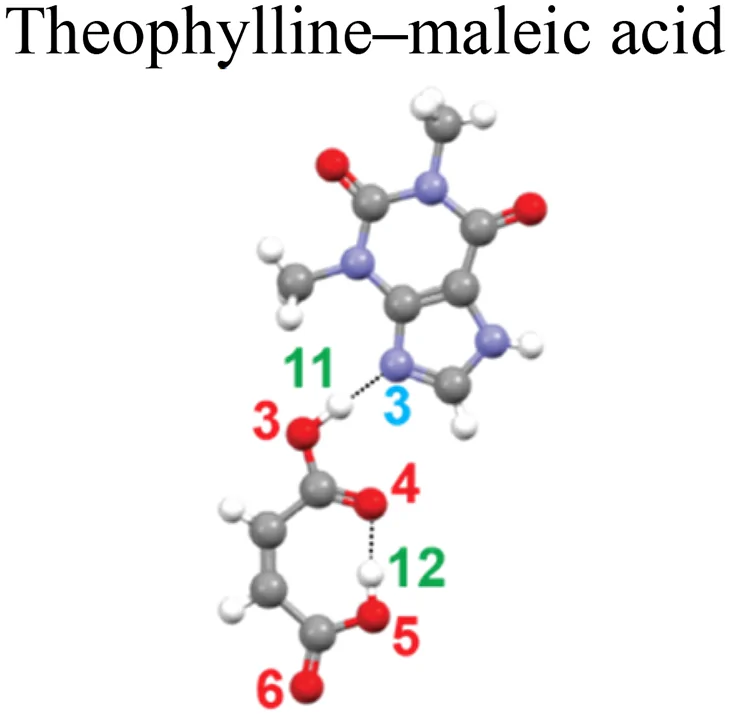	H-11	16.5			15.7			0.8		
	H-12	14.5			14.1			0.4		
	O-3	240^e^			198	6.8	0.09			
	O-4	240^e^			288	7.6	0.26			
	O-5	180	6.9	0.11	183	7.2	0.11	3	0.2	0
	O-6	369	8.9	0.07	364	9.3	0.06	5	0.4	0.01
	N-3	–167	2.7	0.67	–176	2.7	0.67	9	0.0	0
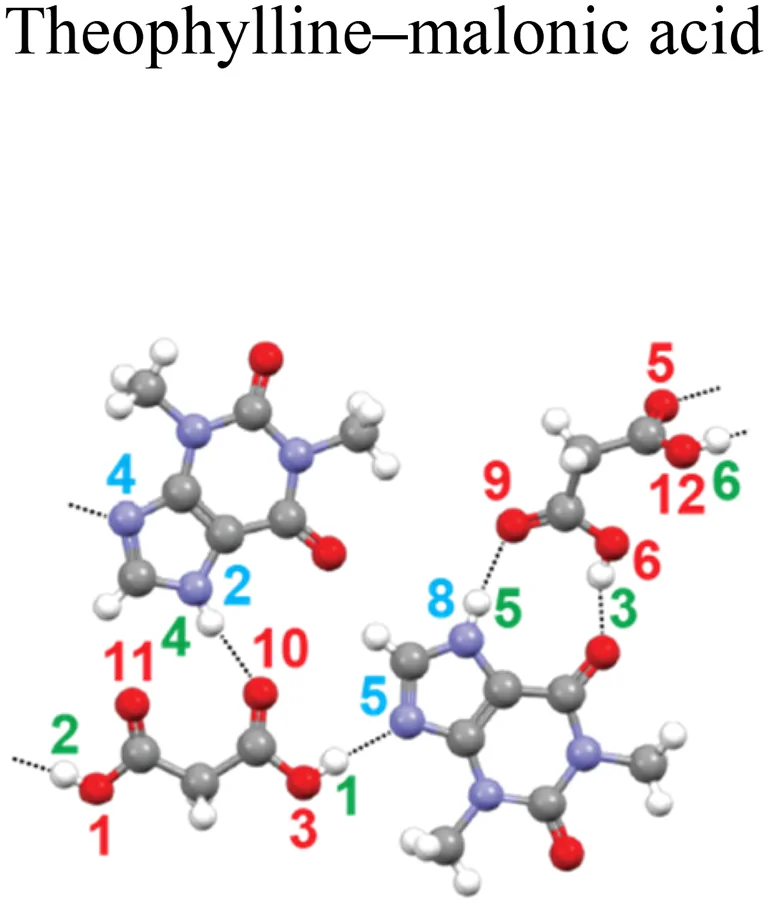	H-1	14.5			14.2			0.3		
	H-2	14.5			13.4			1.1		
	H-3	13.1			12.6			0.5		
	H-4	12.4			11.8			0.6		
	H-5	12.4			11.6			0.8		
	H-6	13.1			13.8			0.7		
	O-1	188	5.9	0.20	175	7.2	0.14	13	1.3	0.06
	O-3	177	4.5	0.30	174	7.2	0.20	3	2.7	0.10
	O-5	240^f^			290	7.7	0.37			
	O-12	240^f^			172	7.0	0.09			
	O-6	175	6.3	0.10	170	7.4	0.13	5	1.1	0.03
	O-9	328	7.6	0.09	293	8.0	0.09	35	0.4	0
	O-10	336	8.3	0.10	300	8.6	0.04	36	0.3	0.06
	O-11	347	8.5	0.05	305	8.8	0.12	42	0.3	0.07
	N-5	–166	2.9	0.60	–177	2.9	0.54	11	0.0	0.06
	N-2	–228	1.6	0.98	–227	1.7	0.93	1	0.0	0.05

a Chemical shifts (*δ*, ppm).

b Absolute quadrupolar coupling constants (|*C*_Q_|, MHz).

c Asymmetry parameters (*η*_Q_).

d Absolute difference between experimental and DFT calculated values.

e An averaged signal centered at *ca.* 240 ppm is attributed to dynamic exchange between oxygen sites O-3 and O-4.

f An averaged signal centered at *ca.* 240 ppm is attributed to dynamic exchange between oxygen sites O-5 and O-12. Experimental ^17^O parameters for the benzoic and oxalic acid cocrystals were obtained from fits to the 14.T spectra, whereas those for maleic and malonic were obtained from fits to the 25.8 T spectra. Experimental ^14^N parameters were obtained from fits to the 9.4 T spectra. DFT calculated shieldings were converted to shifts using previously reported calibration relationships.^53,82^ Additional DFT calculated NMR parameters are provided in the SI.

## Conclusion

This work establishes a multinuclear SSNMR strategy for directly probing hydrogen bonding interactions in pharmaceutical cocrystals. Facile ^17^O enrichment of carboxylic acid coformers was achieved through isotope exchange with ^17^O-enriched water under mild conditions, and the exchange reactions were conveniently monitored by ^17^O solution NMR spectroscopy. The combination of ^1^H-detected ^17^O → ^1^H D-RINEPT and ^1^H{^14^N} D-HMQC experiments enabled assignment of overlapping ^1^H resonances and direct identification of hydrogen bonding interactions in theophylline cocrystals with benzoic, oxalic, maleic, and malonic acid coformers. The combination of ^1^H-detected ^14^N and ^17^O solid-state NMR experiments directly enables inter-molecular connectivity to be determined without resorting to chemical shift analysis or DFT modeling. Variation of the dipolar recoupling time in the D-RINEPT experiments further provided qualitative information on spatial proximities, with shorter recoupling times preferentially highlighting directly bonded or very close nuclei, while longer recoupling times reveal additional longer-range correlations within the hydrogen bonding network. Complementary ^1^H spin-diffusion and dipolar double-quantum experiments provided additional evidence for intermolecular proximities and further corroborated the connectivity with the hydrogen bonded networks. Experimental ^17^O and ^14^N NMR parameters were in good agreement with plane-wave DFT calculations, supporting the structural assignments derived from the correlation experiments. Measurements performed at high magnetic field strengths (up to 25.8 T) further improved the resolution of the ^17^O NMR spectra, enabling distinct oxygen environments and dynamic exchange processes within the cocrystals to be clearly distinguished. With access to higher magnetic fields and faster MAS frequencies it should be possible to further enhance ^1^H, ^14^N and ^17^O spectral resolution, enabling application of these types of experiments to biomolecules where understanding intermolecular hydrogen bonding interactions is of key importance. In this context, ^13^C-detection of ^17^O NMR spectra and the use of ^15^N (with isotope labels) in place of ^14^N would further enhance spectral resolution, enabling studies of larger systems.^55^

## Methods

### Materials

Malonic acid, d-malic acid, oxalic acid, maleic acid, and theophylline were purchased from Sigma Aldrich, Inc. Methanol, chloroform, and concentrated HCl were purchased from Fisher Scientific. Benzoic acid was purchased from Oakwood Chemical. 39.3% ^17^O-enriched water was purchased from CortecNet Inc. Oxalic acid was recrystallized in distilled water and dried open to the atmosphere on the benchtop to form the α-dihydrate polymorph. All other materials were used as received without further purification.

### Solution NMR spectroscopy

Solution experiments were recorded on a 14.1 T (ν_0_(^1^H) = 600 MHz) Bruker standard-bore magnet equipped with an AVANCE III console and a BBFO double-resonance probe. The solution ^17^O NMR spectra were referenced to H_2_O with the ^17^O resonance set to 0 ppm.

### Solid-state NMR spectroscopy

Solid-state NMR experiments were performed on Bruker spectrometers operating at magnetic field strengths of 9.4 T, 14.1 T, and 25.8 T, corresponding to ^1^H Larmor frequencies of 400 MHz, 600 MHZ, and 1.1 GHz, respectively. Experiments performed at Iowa State University primarily utilized a 9.4 T Burker wide-bore magnet equipped with a Avance III HD console and a 1.3 mm HX MAS probe configured in ^1^H–^17^O or ^1^H–^14^N mode. Experiments performed at Ames National Laboratory utilized a 14.1 T Bruker wide-bore magnet equipped with a Bruker NEO console and a 1.3 mm Phoenix HXY probe configured in ^1^H–^17^O double resonance mode. Experiments performed at the National Magnetic Resonance Facility at Madison utilized a 25.8 T Bruker narrow-bore magnet equipped with an Avance NEO console and a 1.2 mm Phoenix HFXY MAS probe configured in ^1^H–^17^O or ^1^H–^14^N mode. Samples were packed into 1.3 mm or 1.2 mm zirconia rotors and spun at magic-angle spinning (MAS) frequencies of 50 kHz, unless stated otherwise. ^1^H chemical shifts were referenced to neat tetramethyl silane (TMS) by using adamantane as a secondary standard (*δ*(^1^H)  =  1.82 ppm). ^17^O and ^14^N chemical shifts were indirectly referenced to H_2_O and NH_3_, respectively, using the previously published IUPAC recommended relative NMR frequencies.^83^^1^H spin-echo spectra were acquired using a standard Hahn echo pulse sequence to reduce probe background signals. Two-dimensional ^17^O → ^1^H dipolar correlation spectra were acquired using a D-RINEPT pulse sequence employing *S*R4^2^_1_ symmetry-based dipolar recoupling.^52,54,55,84^ Two-dimensional ^1^H{^14^N} dipolar correlation spectra were acquired using a ^1^H{^14^N} D-HMQC pulse sequence with *S*R4^2^_1_ dipolar recoupling and rotor-synchronized ^14^N excitation pulses of one rotor cycle duration.^52,57,59,60,77,79^ Additional proton–proton 2D correlation NMR experiments were performed using ^1^H spin-diffusion and ^1^H single-quantum–double-quantum (SQ–DQ) dipolar correlation experiments^85^ to probe intermolecular proton proximities. The back-to-back (BABA) recoupling sequence was used for acquisition of dipolar SQ–DQ 2D NMR spectra.^85^ Detailed acquisition parameters for all NMR experiments are provided in the SI.

### Plane-wave DFT calculations

Periodic density functional theory (DFT) calculations were performed using the CASTEP code^86^ (materials studio CASTEP version 2018). Initial crystal structures were obtained as crystallographic information files (CIFs) from the Cambridge Structural Database (CSD).^87^ Hydrogen atom positions were geometry optimized while all non-hydrogen atomic coordinates and lattice parameters were fixed to the experimental crystallographic values. Geometry optimization was performed using the BFGS minimization algorithm. Calculations employed the Perdew–Burke–Ernzerhof (PBE) exchange–correlation functional together with the Tkatchenko–Scheffler (TS) semi-empirical dispersion correction to account for intermolecular interactions in the molecular crystals. A plane-wave basis set cutoff energy of 630 eV was used. Convergence of the calculated ^1^H, ^14^N, and ^17^O NMR parameters with respect to the plane-wave cutoff energy was verified by comparison with calculations performed at higher cutoff energies. Brillouin zone sampling was performed using a 2 × 1 × 2 Monkhorst–Pack *k*-point grid. NMR parameters were calculated using the CASTEP magnetic resonance module with the gauge-including projector augmented wave (GIPAW) method.^88^ Chemical shielding tensors and electric field gradient (EFG) tensors were obtained for all atoms. Calculated chemical shieldings (*σ*) were converted to shifts (*δ*) using previously reported shielding-to-shift calibration relationships for ^14^N and ^17^O (see SI).

### Powder X-ray diffraction (PXRD)

PXRD was performed on a Rigaku Ultima IV Diffractometer using Cu radiation (Kα, *λ* = 1.54056 Å). Data were collected from 5° to 50° at a continuous scan rate of 0.03° min^−1^. Powder patterns were compared to simulated powder patterns calculated using Mercury software^89^ employing crystallographic information files (CIF) from single crystal X-ray experiments obtained from Cambridge structural database (CSD).^87^

## Author contributions

J. P. B. synthesized the theophylline cocrystals, performed the PXRD, solution NMR, and solid-state NMR experiments (except the 25.8 T experiments), carried out the DFT calculations, analyzed and interpreted the data, and wrote the manuscript. K. R. and S. L. C. developed the initial ^17^O-labeling methodology for carboxylic acids and performed preliminary solution and solid-state NMR experiments. A. L. P. performed 25.8 T solid-state NMR experiments at the National Magnetic Resonance Facility at Madison. A. J. R. contributed to the conception and scientific direction of the cocrystal study, developed the solid-state NMR methodologies, performed the 25.8 T solid-state NMR experiments, supervised the project, discussed the interpretation of the results, and edited the manuscript. All authors reviewed and approved the final manuscript.

## Conflicts of interest

There are no conflicts of interest to declare.

## Supplementary Material

CP-028-D6CP02064J-s001

## Data Availability

Raw NMR data, pulse sequences and CASTEP outputs are available at https://zenodo.org/records/20142311 or https://doi.org/10.5281/zenodo.20142311. Supplementary information (SI): provides detailed experimental procedures, additional solid-state NMR spectra, PXRD data, and computational results referenced in the main text. Additional 2D ^17^O → ^1^H and ^1^H{^14^N} correlation spectra, homonuclear ^1^H–^1^H correlation experiments, spectral fits, and pulse sequences are provided to support the assignments and hydrogen bonding analyses discussed in the manuscript. See DOI: https://doi.org/10.1039/d6cp02064j.

## References

[cit1] Rodríguez-Spong B., Price C. P., Jayasankar A., Matzger A., Rodríguez-Hornedo N. (2004). General Principles of Pharmaceutical Solid Polymorphism: A Supramolecular Perspective. Adv. Drug Delivery Rev..

[cit2] Singhal D., Curatolo W. (2004). Drug Polymorphism and Dosage Form Design: A Practical Perspective. Adv. Drug Delivery Rev..

[cit3] Aaltonen J., Alleso M., Mirza S., Koradia V., Gordon K. C., Rantanen J. (2009). Solid Form Screening—A Review. Eur. J. Pharm. Biopharm..

[cit4] Healy A. M., Worku Z. A., Kumar D., Madi A. M. (2017). Pharmaceutical solvates, hydrates, and amorphous forms: A emphasis on cocrystals. Adv. Drug Delivery Rev..

[cit5] Shi Y., Zhang W., Wang B., Sun C. (2022). Recent Advances in Drug Polymorphs: Aspects of Solid-State Properties and Selective Crystallization. Int. J. Pharm. Sci..

[cit6] FDA. Guidance for Industry: ANDAs - Pharmaceutical Solid Polymorphism Chemistry, Manufacturing, and Controls Information, 2007

[cit7] Trask A. V. (2007). An overview of pharmaceutical cocrystals as intellectual property. Mol. Pharm..

[cit8] Hoffman M. and Lindeman J. A., Co-Crystals: Commercial Opportunities and Patent Considerations. In Pharmaceutical Salts and Co-Crystals, ed Wouters, J., Quéré, L., Royal Society of Chemistry, Cambridge, UK, 2011, pp. 318–329

[cit9] FDA. Guidance for Industry: Regulatory Classification of Pharmaceutical Co-Crystals Guidance for Industry, 2018

[cit10] Almarsson O., Zaworotko M. J. (2004). Crystal engineering of the composition of pharmaceutical phases. Do pharmaceutical co-crystals represent a new path to improved medicines?. Chem. Commun..

[cit11] Aakeröy C. B., Salmon D. J. (2005). Building Co-Crystals with Molecular Sense and Supramolecular Sensibility. CrystEngComm.

[cit12] Vishweshwar P., McMahon J. A., Bis J. A., Zaworotko M. J. (2006). Pharmaceutical Co-Crystals. J. Pharm. Sci..

[cit13] Shan N., Zaworotko M. J. (2008). The Role of Cocrystals in Pharmaceutical Science. Drug Discovery Today.

[cit14] Schultheiss N., Newman A. (2009). Pharmaceutical cocrystals and their physicochemical properties. Cryst. Growth Des..

[cit15] Almarsson O., Peterson M. L., Zaworotko M. (2012). The A to Z of pharmaceutical cocrystals: a decade of fast-moving new science and patents. Pharm. Pat. Analyst.

[cit16] Steed J. W. (2013). The role of co-crystals in pharmaceutical design. Tips.

[cit17] Aitipamula S., Chow P. S., Tan R. B. H. H. (2014). Polymorphism in Cocrystals: A Review and Assessment of Its Significance. CrystEngComm.

[cit18] Bolla G., Nangia A. (2016). Pharmaceutical Cocrystals: Walking the Talk. Chem. Commun..

[cit19] Vioglio P. C., Chierotti M. R., Gobetto R. (2017). Pharmaceutical aspects of salt and cocrystal forms of APIs and characterization challenges. Adv. Drug Delivery Rev..

[cit20] Karimi-Jafari M., Padrela L., Walker G. M., Croker D. M. (2018). Creating Cocrystals: A Review of Pharmaceutical Cocrystal Preparation Routes and Applications. Cryst. Growth Des..

[cit21] Guo M., Sun X., Chen J., Cai T. (2021). Pharmaceutical cocrystals: A review of preparations, physicochemical properties and applications. Acta Pharm. Sin. B..

[cit22] Bolla G., Sarma B., Nangia A. K. (2022). Crystal Engineering of Pharmaceutical Cocrystals in the Discovery and Development of Improved Drugs. Chem. Rev..

[cit23] Singh M., Barua H., Sainaga Jyothi V. G. S., Dhondale M. R., Nambiar A. G., Agrawal A. K., Kumar P., Shastri N. R., Kumar D. (2023). Cocrystals by design: A rational conformer selection approach for tackling the API problems. Pharmaceutics.

[cit24] Chettri A., Subba A., Singh G. P., Bag P. P. (2024). Pharmaceutical co-crystals: A green way to enhance drug stability and solubility for improved therapeutic efficacy. J. Pharm. Pharmacol..

[cit25] Aitipamula S., Banerjee R., Bansal A. K., Biradha K., Cheney M. L., Choudhury A. R., Desiraju G. R., Dikundwar A. G., Dubey R., Duggirala N., Ghogale P. P., Ghosh S., Goswami P. K., Goud N. R., Jetti R. R. K. R., Karpinski P., Kaushik P., Kumar D., Kumar V., Moulton B., Mukherjee A., Mukherjee G., Myerson A. S., Puri V., Ramanan A., Rajamannar T., Reddy C. M., Rodriguez-Hornedo N., Rogers R. D., Row T. N. G., Sanphui P., Shan N., Shete G., Singh A., Sun C. C., Swift J. A., Thaimattam R., Thakur T. S., Kumar Thaper R., Thomas S. P., Tothadi S., Vangala V. R., Variankaval N., Vishweshwar P., Weyna D. R., Zaworotko M. J. (2012). Polymorphs, Salts, and Cocrystals: What's in a Name?. Cryst. Growth Des..

[cit26] Cruz-Cabeza A. J. (2012). Acid–base crystalline complexes and the pK_a_ rule. CrystEngComm.

[cit27] Childs S. L., Stahly G. P., Park A. (2007). The Salt-Cocrystal Continuum: The Influence of Crystal Structure on Ionization State. Mol. Pharm..

[cit28] Cruz-Cabeza A. J., Lusi M., Wheatcroft H. P., Bond A. D. (2022). The Role of Solvation in Proton Transfer Reactions: Implications for Predicting Salt/Co-crystal Formation Using the ΔpK_a_ Rule. Faraday Discuss..

[cit29] Newman J. A., Iuzzolino L., Tan M., Orth P., Bruhn J., Lee A. Y. (2022). From Powders to Single Crystals: A Crystallographer's Toolbox for Small-Molecule Structure Determination. Mol. Pharmaceutics.

[cit30] Chappa P., Jenjeti R. N., Kljun J., Mittapalli S., Pichler A., Desiraju G. R. (2025). Pharmaceutical Solid Form Screening and Selection: Which form emerges?. Cryst. Growth Des..

[cit31] Xiao Y., Wu C., Zhou L., Commins P., Li L., Naumov P., Yin Q. (2024). Current Trends and Advancements in Crystallization and Single-Crystal Structural Analysis of Small Molecules. Coord. Chem. Rev..

[cit32] Gruene T., Wennmacher J. T. C., Zaubitzer C., Holstein J. J., Heidler J., Fecteau-Lefebvre A., De Carlo S., Muller E., Goldie K. N., Regeni I., Li T., Santiso-Quinones G., Steinfeld G., Handschin S., van Genderen E., van Bokhoven J. A., Clever G. H., Pantelic R. (2018). Rapid Structure Determination of Microcrystalline Molecular Compounds Using Electron Diffraction. Angew. Chem., Int. Ed..

[cit33] Friščić T., Jones W. (2009). Recent Advances in Understanding the Mechanism of Cocrystal Formation via Grinding. Cryst. Growth Des..

[cit34] Braga D., Maini L., Grepioni F. (2013). Mechanochemical preparation of co-crystals. Chem. Soc. Rev..

[cit35] Steiner T. (2002). The hydrogen bond in the solid state. Angew. Chem., Int. Ed..

[cit36] Cooper R. I., Thompson A. L., Watkin D. J. (2010). CRYSTALS enhancements: dealing with hydrogen atoms in refinement. J. Appl. Cryst..

[cit37] Vogt F. G., Clawson J. S., Strohmeier M., Edwards A. J., Pham T. N., Watson S. A. (2009). Solid-state NMR analysis of organic cocrystals and complexes. Cryst. Growth Des..

[cit38] Khan M., Enkelmann V., Brunklaus G. (2010). Crystal Engineering of Pharmaceutical Co-crystals: Application of Methyl Paraben as Molecular Hook. J. Am. Chem. Soc..

[cit39] Tatton A. S., Pham T. N., Vogt F. G., Iuga D., Edwards A. J., Brown S. P. (2013). Probing Hydrogen Bonding in Cocrystals and Amorphous Dispersions Using ^14^N−^1^H HMQC Solid-State NMR. Mol. Pharmaceutics.

[cit40] Vogt F. G., Yin H., Forcino R. G., Wu L. (2013). ^17^O Solid-State NMR as a Sensitive Probe of Hydrogen Bonding in Crystalline and Amorphous Solid Forms of Diflunisal. Mol. Pharmaceutics.

[cit41] Stevens J. S., Byard S. J., Seaton C. C., Sadiq G., Davey R. J., Schroeder S. L. M. (2014). Proton transfer and hydrogen bonding in the organic solid state: a XRD/XPS/ssNMR study of 17 organic acid-base complexes. Phys. Chem. Chem. Phys..

[cit42] Pindelska E., Sokal A., Szeleszczuk L., Pisklak D. M., Kolodziejski W. (2014). Solid-state NMR studies of theophylline co-crystals with dicarboxylic acids. J. Pharm. Biomed. Anal..

[cit43] Lüdeker D., Brunklaus G. (2015). NMR crystallography of ezetimibe co-crystals. Solid State Nucl. Magn. Reson..

[cit44] Luedeker D., Gossmann R., Langer K., Brunklaus G. (2016). Crystal engineering of pharmaceutical co-crystals: “NMR crystallography” of niclosamide co-crystals. Cryst. Growth Des..

[cit45] Rajput L., Banik M., Yarava J. R., Joseph S., Pandey M. K., Nishiyama Y., Desiraju G. R. (2017). Exploring the Salt-Cocrystal Continuum with Solid-state NMR Using Natural Abundance Samples: Implications for Crystal Engineering. IUCrJ.

[cit46] Pindelska E., Sokal A., Kolodziejski W. (2017). Pharmaceutical Cocrystals, Salts and Polymorphs: Advanced Characterization Techniques. Adv. Drug Delivery Rev..

[cit47] Zhao L., Hanrahan M. P., Chakravarty P., DiPasquale A. G., Sirois L. E., Nagapudi K., Lubach J. W., Rosssini A. J. (2018). Characterization of Pharmaceutical Cocrystals and Salts by Dynamic Nuclear Polarization-Enhanced Solid-State NMR Spectroscopy. Cryst. Growth Des..

[cit48] Wijesekara A. V., Venkatesh A., Lampkin B. J., VanVeller B., Lubach J. W., Nagapudi K., Hung I., Gor’kov P. L., Zhehong Gan Z., Rossini A. J. (2020). Fast Acquisition of Proton-Detected HETCOR Solid-State NMR Spectra of Quadrupolar Nuclei and Rapid Measurement of NH Bond Lengths by Frequency Selective HMQC and RESPDOR Pulse Sequences. Chem. – Eur. J..

[cit49] Bernasconi D., Bordignon S., Rossi F., Priola E., Nervi C., Gobetto R., Voinovich D., Hasa D., Duong N. T., Nishiyama Y., Chierotti M. R. (2020). Selective Synthesis of a Salt and a Cocrystal of the Ethionamide–Salicylic Acid System. Cryst. Growth Des..

[cit50] Szell P. M. J., Lewandowski J. R., Blade H., Hughes L. P., Lill S. O. N., Brown S. P. (2021). Taming the dynamics in a pharmaceutical by cocrystallization: investigating the impact of the conformer by solid-state NMR. CrystEngComm.

[cit51] Grosu I. G., Iuga D., Tatman B. P., Miclaus M. O., Brown S. P., Filip C. (2024). Salt/co-crystal discrimination made easy by ^17^O solid-state NMR. Chem. Methods.

[cit52] Brinkmann A., Kentgens A. P. M. (2006). Proton-Selective ^17^O–^1^H Distance Measurements in Fast Magic-Angle-Spinning Solid-State NMR Spectroscopy for the Determination of Hydrogen Bond Lengths. J. Am. Chem. Soc..

[cit53] Carnahan S. L., Lampkin B. J., Naik P., Hanrahan M. P., Slowing I. I., VanVeller B., Wu G., Rossini A. J. (2019). Probing O−H Bonding through Proton Detected ^1^H−^17^O Double Resonance Solid-State NMR Spectroscopy. J. Am. Chem. Soc..

[cit54] Venkatesh A., Hanrahan M. P., Rossini A. J. (2017). Proton Detection of MAS Solid-State NMR Spectra of Half-Integer Quadrupolar Nuclei. Solid State Nucl. Magn. Reson..

[cit55] Keeler E. G., Michaelis V. K., Colvin M. T., Hung I., Gor’kov P. L., Cross T. A., Gan Z., Griffin R. G. (2017). ^17^O MAS NMR Correlation Spectroscopy at High Magnetic Fields. J. Am. Chem. Soc..

[cit56] Wu G., Dai Y., Hung I., Gan Z., Matlinska M. A., Wasylishen R. E. (2025). Probing location of hydrogen atoms in low-barrier hydrogen bonds in 1,3-diketone molecules by H/D isotope shifts in solid state ^17^O nuclear magnetic resonance and ^1^H–^17^O distance measurements. J. Phys. Chem. A.

[cit57] Nishiyama Y., Endo Y., Nemoto T., Utsumi H., Yamauchi K., Hioka K., Asakura T. (2011). Very fast magic angle spinning ^1^H-^14^N 2D solid-state NMR: Sub-micro-liter sample data collection in a few minutes. J. Magn. Reson..

[cit58] Grüne M., Luxenhofer R., Iuga D., Brown S. P., Pöppler A.-C. (2020). ^14^N–^1^H HMQC Solid-State NMR as a Powerful Tool to Study Amorphous Formulations – An Exemplary Study of Paclitaxel Loaded Polymer Micelles. J. Mater. Chem. B.

[cit59] Cavadini S., Antonijevic S., Lupulescu A., Bodenhausen G. (2007). Indirect detection of nitrogen-14 in solid-state NMR spectroscopy. ChemPhysChem.

[cit60] Cavadini S., Abraham A., Bodenhausen G. (2007). Proton-detected nitrogen-14 NMR by recoupling of heteronuclear dipolar interactions using symmetry-based sequences. Chem. Phys. Lett..

[cit61] Wei J., Antzutkin O. N., Filippov A. V., Iuga D., Lam P. Y., Barrow M. P., Dupree R., Brown S. P., O’Connor P. B. (2016). Amyloid hydrogen bonding polymorphism evaluated by ^15^N{^17^O}REAPDOR solid-state NMR and ultra-high resolution Fourier Transform ion cyclotron resonance mass spectrometry. Biochemistry.

[cit62] Hung I., Uldry A.-C., Becker-Baldus J., Webber A. L., Wong A., Smith M. E., Joyce S. A., Yates J. R., Pickard C. J., Dupree R., Brown S. P. (2009). Probing heteronuclear ^15^N–^17^O and ^13^C–^17^O connectivities and proximities by solid-state NMR spectroscopy. J. Am. Chem. Soc..

[cit63] Tatman B. P., Modha H., Brown S. P. (2023). Comparison of methods for ^14^N-^1^H recoupling in ^14^N-^1^H HMQC MAS NMR. J. Magn. Reson..

[cit64] Barnes P. J. (2013). Pulmonary Perspectives: Theophylline. Am. J. Respir. Crit. Care Med..

[cit65] Huang Y., Zhou L., Yang W., Li Y., Yang Y., Zhang Z., Wang C., Zhang X., Yun Q. (2019). Preparation of Theophylline-Benzoic Acid Cocrystal and On-Line Monitoring of Cocrystallization Process in Solution by Raman Spectroscopy. Crystals.

[cit66] Corpinot M. K., Stratford S. A., Arhangelskis M., Anka-Lufford J., Halasz I., Judaš N., Jones W., Bučar D. (2016). On the predictability of supramolecular interactions in molecular cocrystals – the view from the bench. CrystEngComm.

[cit67] Trask A. V., Motherwell W. D. S., Jones W. (2006). Physical stability enhancement of theophylline via cocrystallization. Int. J. Pharm..

[cit68] Macchietti L., Casali L., Emmerling F., Braga D., Grepioni F. (2024). Deriving kinetic insights from mechanochemically synthesized compounds using multivariate analysis (MCR-ALS) of powder X-ray diffraction data. RSC Mechanochem..

[cit69] Redington R. L. (1976). Kinetics of oxygen-18 exchange between carboxylic acids and water. J. Phys. Chem..

[cit70] Métro T.-X., Gervais C., Martinez A., Bonhomme C., Laurencin D. (2017). Unleashing the Potential of 17O Nmr Spectroscopy Using Mechanochemistry. Angew. Chem., Int. Ed..

[cit71] Špačková J., Fabra C., Mittelette S., Gaillard E., Chen C.-H., Cazals G., Lebrun A., Sene S., Berthomieu D., Chen K., Gan Z., Gervais C., Métro T.-X., Laurencin D. (2020). Unveiling the Structure and Reactivity of Fatty-Acid Based (Nano)Materials Thanks to Efficient and Scalable 17O and 18O-Isotopic Labeling Schemes. J. Am. Chem. Soc..

[cit72] Seyfried M. S., Lauber B. S., Luedtke N. W. (2010). Multiple-Turnover Isotopic Labeling of Fmoc- and Boc-Protected Amino Acids with Oxygen Isotopes. Org. Lett..

[cit73] Theodorou V., Skobridis K., Alivertis D., Gerothanassis I. P. (2014). Synthetic Methodologies in Organic Chemistry Involving Incorporation of O-17 and O-18 Isotopes. J. Labelled Comp. Radiopharm..

[cit74] Wu G. (2016). Solid-State O-17 NMR Studies of Organic and Biological Molecules: Recent Advances and Future Directions. Solid State Nucl. Magn. Reson..

[cit75] Pike K. J., Lemaitre V., Kukol A., Anupõld T., Samoson A., Howes A. P., Watts A., Smith M. E., Dupree R. (2004). Solid-State ^17^O NMR of Amino Acids. J. Phys. Chem. B.

[cit76] Hagaman E. W., Chen B., Jiao J., Parsons W. (2012). Solid-state ^17^O NMR study of benzoic acid adsorption on metal oxide surfaces. Solid State Nucl. Magn. Reson..

[cit77] Amoureux J. P., Trebosc J., Wiench J., Pruski M. (2007). HMQC and Refocused-INEPT Experiments. Involving Half-Integer Quadrupolar Nuclei in Solids. J. Magn. Reson..

[cit78] Gan Z. (2007). ^13^C/^14^N heteronuclear multiple-quantum correlation with rotary resonance and REDOR dipolar recoupling. J. Magn. Reson..

[cit79] Shen M., Trébosc J., O’Dell L. A., Lafon O., Pourpoint F., Hu B., Chen Q., Amoureux J.-P. (2015). Comparison of Various NMR Methods for the Indirect Detection of Nitrogen-14 Nuclei via Protons in Solids. J. Magn. Reson..

[cit80] Lu J., Hung I., Brinkmann A., Gan Z., Kong X., Wu G. (2017). Solid-state ^17^O NMR reveals hydrogen-bonding energetics: not all low-barrier hydrogen bonds are strong. Angew. Chem., Int. Ed..

[cit81] Štoček J. R., Socha O., Císařová I., Slanina T., Dracínský M. (2022). Importance of Nuclear Quantum Effects for Molecular Cocrystals with Short Hydrogen Bonds. J. Am. Chem. Soc..

[cit82] Dorn R. W., Ryan M. J., Kim T.-H., Goh T. W., Venkatesh A., Heintz P. M., Zhou L., Huang W., Rossini A. J. (2020). Identifying the Molecular Edge Termination of Exfoliated Hexagonal Boron Nitride Nanosheets with Solid-State NMR Spectroscopy and Plane-Wave DFT Calculations. Chem. Mater..

[cit83] Harris R. K., Becker E. D., Cabral de Menezes S. M., Goodfellow R., Granger P. (2002). NMR nomenclature: nuclear spin properties and conventions for chemical shifts: IUPAC recommendations 2001. Solid State Nucl. Magn. Reson..

[cit84] Trebosc J., Hu B., Amoureux J.-P., Gan Z. (2007). Through-Space R^3^-HETCOR Experiments between Spin-1/2 and Half-Integer Quadrupolar Nuclei in Solid-State NMR. J. Magn. Reson..

[cit85] Feike M., Demco D. E., Graf R., Gottwald J., Hafner S., Spiess H. W. (1996). Broadband Multiple-Quantum NMR Spectroscopy. J. Magn. Reson., Ser. A.

[cit86] Clark S. J., Segall M. D., Pickard C. J., Hasnip P. J., Probert M. I. J., Refson K., Payne M. C. (2005). First principles methods using CASTEP. Z. Kristallogr..

[cit87] Groom C. R., Bruno I. J., Lightfoot M. P., Ward S. C. (2016). The Cambridge Structural Database. Acta Cryst.

[cit88] Yates J. R., Pickard C. J., Mauri F. (2007). Calculation of NMR Chemical Shifts for Extended Systems Using Ultrasoft Pseudopotentials. Phys. Rev. B: Condens. Matter Mater. Phys..

[cit89] Macrae C. F., Sovago I., Cottrell S. J., Galerk P. T. A., McCabe P., Pidcock E., Platings M., Shields G. P., Stevens J. S., Towler M., Wood P. A. (2020). J. AppMercury 4.0: from visualization to analysis, design and prediction. J. Appl. Cryst..

