# Supplementary material for: Elucidation of hydrogen bonding networks in theophylline cocrystals by 1H-detected 17O and 14N solid-state NMR spectroscopy
Source: Phys Chem Chem Phys. 2026 Sep 4;28(37):22943–57. doi: 10.1039/d6cp02064j (PMC13559685; doi:10.1039/d6cp02064j)
Supplement: Supplementary file 1 [file CP-028-D6CP02064J-s001.pdf]

Supplementary Information (SI) for Physical Chemistry Chemical Physics.
This journal is © the Owner Societies 2026

Supplementary Information

Elucidation of Hydrogen Bonding Networks in Theophylline Cocrystals by ^{1}H-Detected ^{17}O and ^{14}N Solid-State NMR Spectroscopy

*Joseph P. Bequette[1], Kipper Riemersma[1], Scott L. Carnahan[1], Alexander L. Paterson[2], Aaron J. Rossini[1,2]**

[1] *Iowa State University, Department of Chemistry, Ames, IA, USA 50011*
[2] *National Magnetic Resonance Facility at Madison (NMRFAM), University of Wisconsin-Madison, Madison, Wisconsin, USA, 53706*

AUTHOR INFORMATION
Corresponding Author
*e-mail: arossini@iastate.edu, phone: 515-294-8952

Table of Contents

	Page(s)
**Procedures and Calculations**	
^{17}O labeling of oxalic, maleic, *D*-malic, malonic, and benzoic acids	S3
Calculation to determine ^{17}O % exchanged from solution NMR	S4
Preparation of theophylline-carboxylic acid cocrystals	S5
**Supplementary Figures**	
**Figure S1.** Two-dimensional ^{17}O→^{1}H D-RINEPT and ^{17}O spin echo solid-state NMR spectra of oxalic acid α-dihydrate	S6
**Figure S2.** PXRD patterns of theophylline-carboxylic acid cocrystals and starting reagents	S7 – S12
**Figure S3.** Two-dimensional ^{17}O→^{1}H D-RINEPT solid-state NMR spectra of theophylline– benzoic, maleic, and malonic acid cocrystals with indicated recoupling time	S13
**Figure S4.** Two-dimensional ^{1}H{^{14}N} D-HMQC solid-state NMR spectra of theophylline– benzoic, oxalic, maleic, and malonic acid cocrystals with indicated recoupling time	S14
**Figure S5.** ^{17}O solid-state NMR spectra of the theophylline-oxalic acid cocrystal	S15
**Figure S6.** Two-dimensional homonuclear ^{1}H–^{1}H spin-diffusion single-quantum–single-quantum (SQ–SQ) solid-state NMR spectra of theophylline– benzoic, oxalic, maleic, and malonic acid cocrystals	S15 – S16
**Figure S7.** Two-dimensional homonuclear ^{1}H–^{1}H dipolar single-quantum–double-quantum (SQ–DQ) solid-state NMR spectra of theophylline– oxalic, maleic, and malonic acid cocrystals	S17 – S18
**Figure S8.** ^{17}O spin echo solid-state NMR spectra of theophylline–maleic acid acquired at 25.8 T with comparison to simulated spectral fits	S19
**Figure S9.** ^{17}O spin echo solid-state NMR spectra of theophylline–malonic acid acquired at 25.8 T	S20
**Figure S10.** ^{17}O and ^{14}N solid-state NMR spectral fitting of theophylline cocrystals	S21
**Figure S11.** Solid-state NMR pulse sequences	S22
**Supplementary Tables**	
**Table S1.** Plane-wave DFT calculated ^{17}O, ^{14}N, and ^{1}H NMR parameters	S23 – S25
**Table S2.** Experimental ^{13}C chemical shifts recorded at 25.8 T for adamantane under variable-temperature (VT) conditions	S26
**Table S3.** Comparison between 25.8 T experimental and DFT calculated ^{17}O solid-state NMR parameters	S26
**Table S4.** Comparison between 14.1 T experimental and DFT calculated ^{17}O solid-state NMR parameters	S27
**Table S5.** Comparison between 14.1 experimental and DFT calculated ^{14}N solid-state NMR parameters	S28
**Table S6.** Experimental NMR parameters	S29 – S32
**References**	S33 – S34

^{17}O Labeling of oxalic, maleic, D-malic, malonic, and benzoic acids

Oxalic acid. Oxalic acid dihydrate (49.1 mg) and 39.30% ^{17}O-enriched water (102.9 mg) were added to a standard 1-mL GC vial, capped, and heated in an oven at 80 °C for 25 hours. To remove the water, the vial was uncapped and left in the oven for ca. 24 hours.

Maleic acid. Maleic acid (145.4 mg) and 39.30% ^{17}O-enriched water (82.5 mg) were added to a standard 1-mL GC vial, capped, and heated in an oven at 80 °C for 25 hours. To remove the water, the vial was uncapped and left in the oven for an additional 23 hours, followed by placement under vacuum at room temperature for ca. 24 hours.

***D*-Malic acid**. Malic acid (55.4 mg), 39.30% ^{17}O-enriched water (17.5 mg), and distilled water (89.0 mg) were added to a standard 1-mL GC vial, capped, and heated in an oven at 80 °C for ca. 72 hours. To remove the water, the vial was uncapped and left in the oven for ca. 24 hours.

Malonic acid. Malonic acid (102.8 mg) and 39.30% ^{17}O-enriched water (138.3 mg) were added to a standard 1-mL GC vial, capped, and stored in a drawer for 18 days, after which the vial was uncapped and placed under vacuum for ca. 24 hours to remove the water.

Benzoic acid. Following a similar procedure by Hagaman *et al.*[1], benzoic acid (104.3 mg), 39.3% aqueous ^{17}O-water (290 μL), and concentrated HCl (10 μL) were added to a 3-mL screw top vial, capped, and then heated at 110 °C for ca. 24 hours. After heating, the vial was placed under vacuum for ca. 24 hours to remove the water and HCl.

Calculation to determine ^{17}O % exchanged from solution NMR

% exchanged is given by the fraction of the experimental ^{17}O NMR integral ratio of acid to water (R_{exp}) to the equilibrium integral ratio (R_{eq}) corresponding to 100% exchange and uniform distribution of ^{17}O among all exchangeable oxygen atoms. An example calculation is provided below for a solution of maleic acid (47.8 mg, 0.412 mmol), 39.3% aqueous $H_2{}^{17}O$ (18.3 mg total of water which corresponds to 7.4 mg and 0.390 mmol $H_2{}^{17}O$ and 10.9 mg and 0.603 mmol $H_2{}^{16}O$), and distilled water (88.3 mg, 4.90 mmol). Note, each maleic acid molecule contains 4 exchangeable oxygen atoms.

$$R_{exp} = \frac{I_{acid,exp}}{I_{acid,exp}+I_{water,exp}} = \frac{I_{acid,exp}}{I_{total,exp}} = \frac{491026791}{491026791+2211836252} = 0.182$$

$$R_{eq} = \frac{exchangeabale\ mmol\ O\ from\ acid}{total\ mmol\ of\ exchangeable\ O} = \frac{(0.412 \times 4)\ mmol}{(1.648+0.390 + 0.603 + 4.90)\ mmol} = 0.218$$

$$\%\ \text{exchanged} = \frac{R_{exp}}{R_{eq}} \times 100\% = \frac{0.182}{0.218} \times 100\% = 83\%$$

The equilibrium ^{17}O-labeling % ($^{17}O_{eq}$ %) is given by the fraction of ^{17}O to total exchangeable O × 100%

$$^{17}O_{eq}\ \% = \frac{\text{total moles of 17O}}{\text{total moles of O}} \times 100\% = \frac{0.390\ \text{mmol}}{[1.648 + 0.390 + 0.603 + 4.90]\ \text{mmol}} \times 100\% = 5.18\%$$

(^{17}O natural abundance is 0.037% and is considered negligible)

The experimental ^{17}O-labeling % ($^{17}O_{exp}$ %) of the carboxylic acid is given by the product of % exchanged and $^{17}O_{eq}$ % followed by division of 100%

$$^{17}O_{exp}\ \% = \frac{\%\ \text{exchanged} \times 17O eq\ \%}{100\ \%} = \frac{83\% \times 5.18\%}{100\%} = 4.3\%$$

Preparation of theophylline-carboxylic acid cocrystals

Preparation of theophylline cocrystals were adapted from published procedures[2,3] utilizing the prepared ^{17}O-enriched carboxylic acids.

Theophylline–benzoic acid. ^{17}O-enriched benzoic acid (50.1 mg) and theophylline (72.7 mg) were stirred in MeOH:H_2O (1:5 v/v) (417 µL : 2084 µL) for two days at room temperature to afford a fine suspension. The suspension was centrifuged and the liquid was removed. The remaining wet solid was left to air-dry for two days and subsequently placed under vacuum for 1 hour.

Bis(theophylline)–oxalic acid. ^{17}O-enriched oxalic acid (28.3 mg) and theophylline (109.6 mg) were placed in a narrow 5-mL screw-top vial along with chloroform (2.213 mL) and methanol (609 µL). The mixture was heated to a gentle boil for ca. 5 minutes until all solids were dissolved and then left to stand undisturbed. Crystallization commenced upon cooling and slow evaporation of the solvent. The cocrystals were collected by vacuum filtration and dried under vacuum.

Theophylline–maleic acid. ^{17}O-enriched maleic acid (104.0 mg) and theophylline (160.9 mg) were placed in a narrow 5-mL screw-top vial along with chloroform (2.400 mL), and methanol (400 µL). The mixture was heated to a gentle boil for ca. 5 minutes until all solids were dissolved and then left to stand undisturbed. Crystallization commenced upon cooling and slow evaporation of the solvent. The first batch of crystals contained unreacted theophylline and was set aside. The second batch was collected for analysis.

Theophylline–malonic acid. ^{17}O-enriched malonic acid (82.9 mg) and theophylline (143.5 mg) were placed in a glass vial along with chloroform (9.782 mL), and methanol (489 µL). The mixture was heated to a gentle boil for ca. 5 minutes until all solids were dissolved and then left to stand undisturbed. Crystallization commenced upon cooling and slow evaporation of the solvent. The cocrystals were collected by vacuum filtration and dried under vacuum.

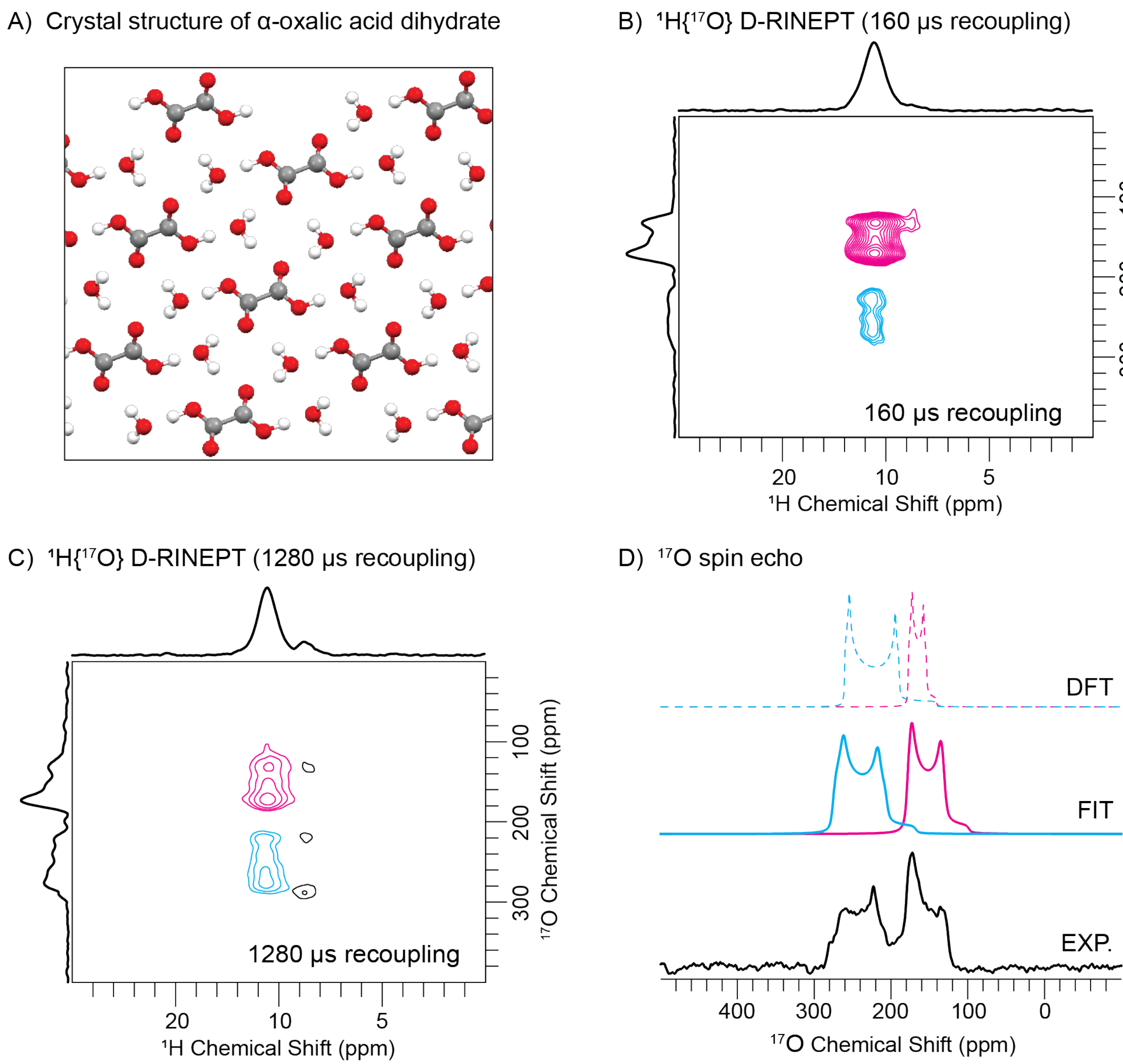

Figure S1. Two-dimensional $^{17}O\rightarrow{}^{1}H$ D-RINEPT and one-dimensional ^{17}O spin echo solid-state NMR spectra of oxalic acid α-dihydrate. (A) X-ray crystal structure of oxalic acid α-dihydrate (CSD OXACDH04[4]). (B) $^{1}H\{^{17}O\}$ D-RINEPT NMR spectrum obtained with 160 μs recoupling. (C) $^{1}H\{^{17}O\}$ D-RINEPT NMR spectrum obtained with 1280 μs recoupling. (D) Experimental ^{17}O spin echo NMR spectrum (bottom) fit with Bruker TopSpin SOLA software[5] (middle) and DFT calculated spectrum (top). DFT calculated and experimental fit NMR parameters for δ_{iso}, C_Q, and η_Q are provided in Tables S1 and S4. Experiments were performed with a 14.1 T magnetic field and a 50 kHz MAS frequency.

PXRD patterns of theophylline-carboxylic acid cocrystals and starting reagents

Experimental PXRD patterns were compared to simulated PXRD patterns calculated using Mercury software[6] employing Crystallographic Information Files (CIF) from single crystal X-ray experiments obtained from Cambridge Structural Database (CSD)[7]

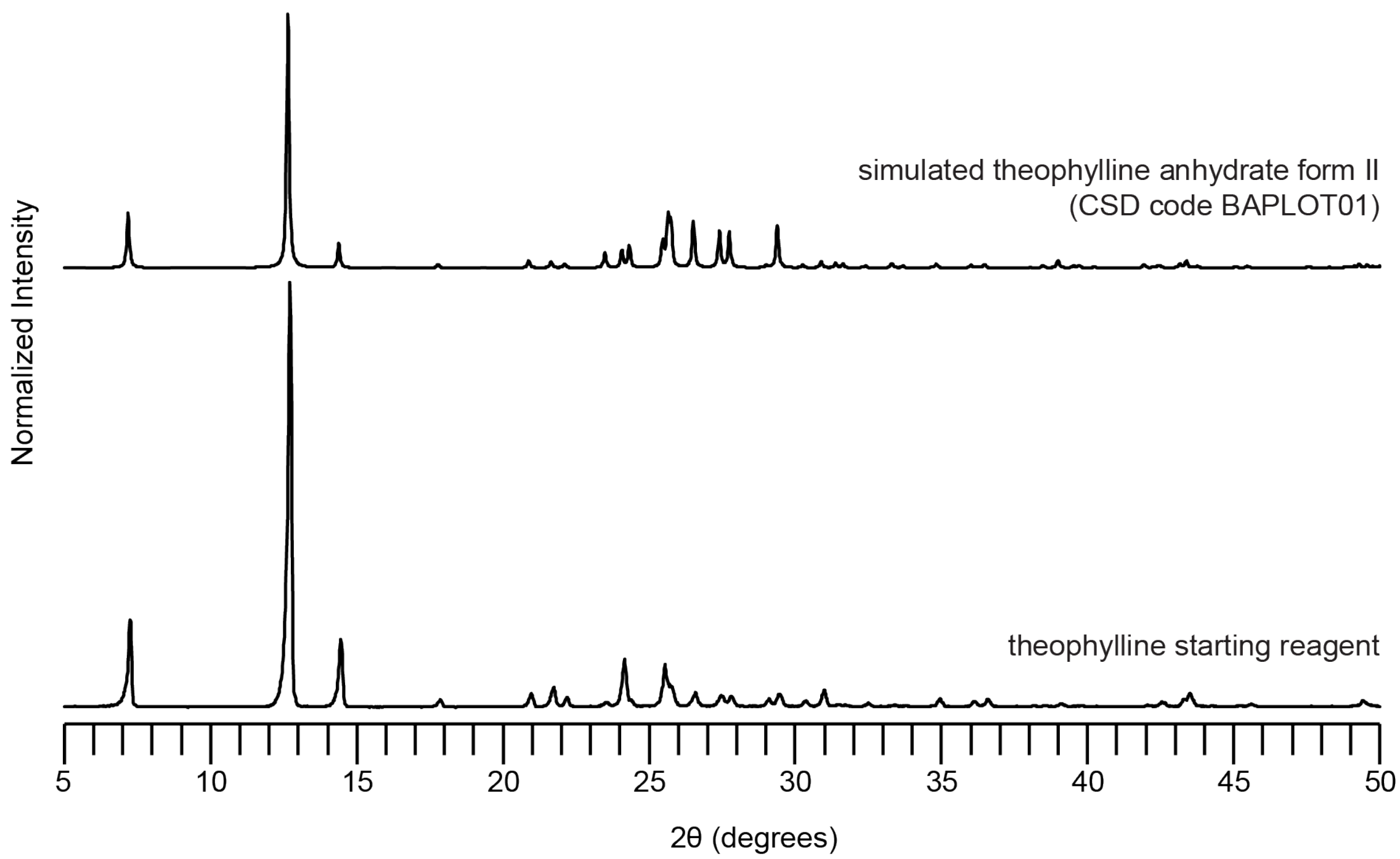

Figure S2A. PXRD pattern of theophylline anhydrate form II. The experimental PXRD pattern of the purchased theophylline starting material (bottom) matches the pattern simulated from the CIF file with CSD code BAPLOT01[8] corresponding to theophylline anhydrate form II (top). The purchased theophylline was used as received for the preparation of all cocrystals

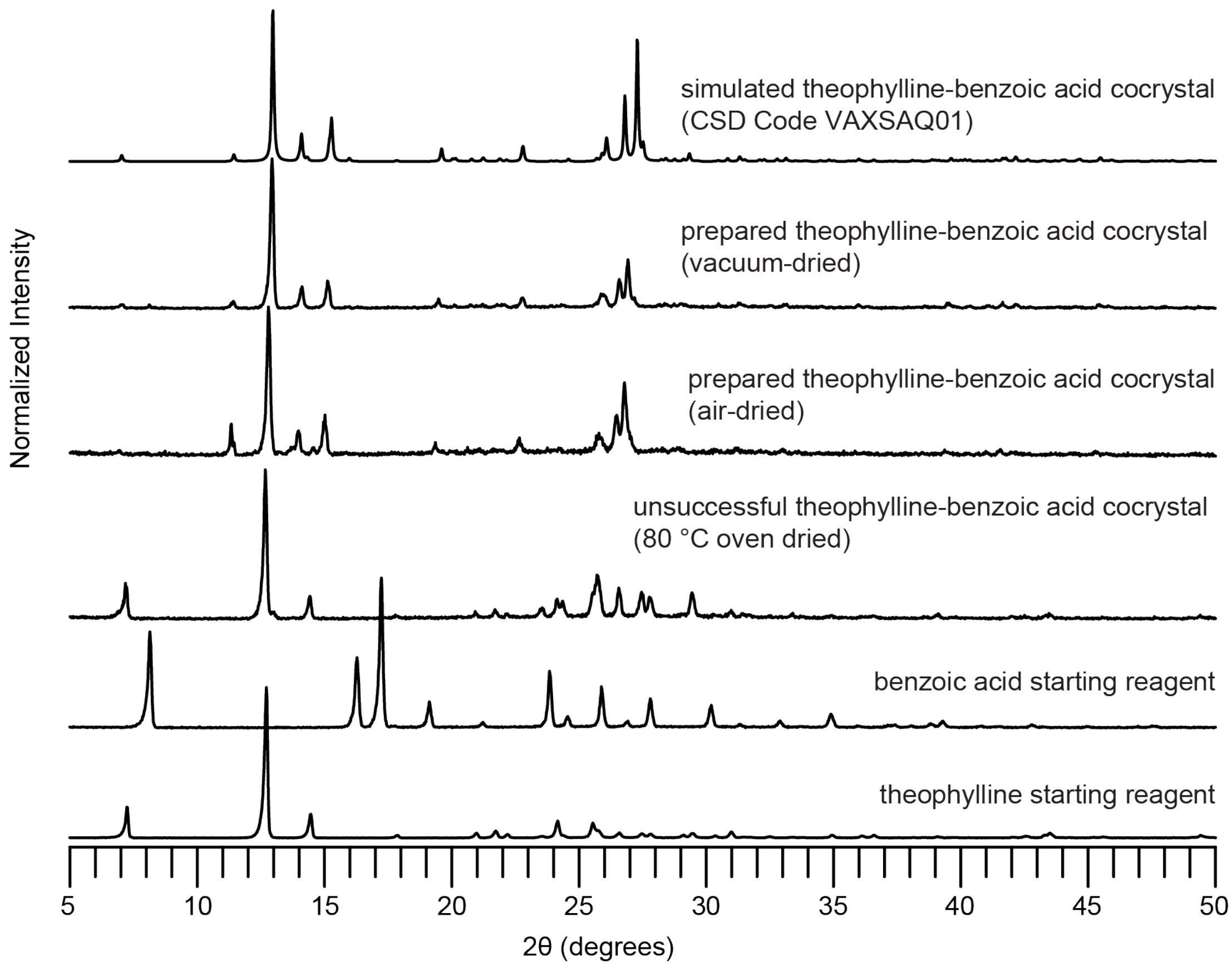

Figure S2B. PXRD patterns for the theophylline–benzoic acid cocrystal. Experimental PXRD patterns of the prepared samples obtained after air drying or vacuum drying match the pattern simulated from the CIF file with CSD code VAXSAQ01[9] for the theophylline–benzoic acid cocrystal (top). Drying the sample at 80 °C did not yield the cocrystal phase, likely due to benzoic acid volatilization. PXRD patterns of the starting materials (theophylline and benzoic acid) are included for comparison.

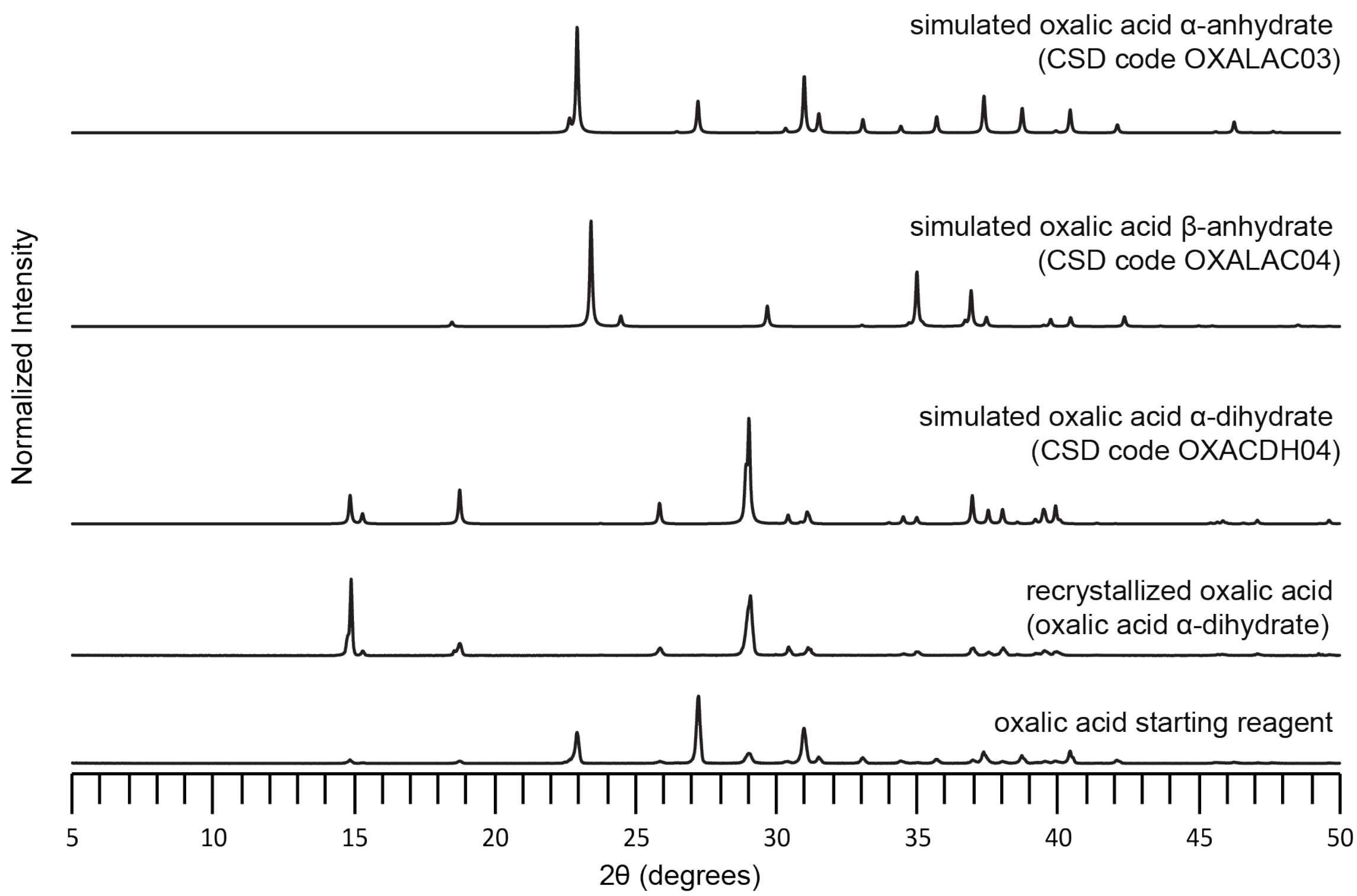

Figure S2C. PXRD patterns of oxalic acid. The experimental PXRD pattern of the purchased oxalic acid starting material (bottom) was compared with simulated patterns generated from CIF files with CSD codes OXALAC03[10], OXALAC04[10], OXACDH04[4]. The starting material was found to be a mixture of anhydrous and hydrated oxalic acid phases. Recrystallization from water afforded a single polymorph corresponding to oxalic acid α-dihydrate, whose PXRD pattern is shown above.

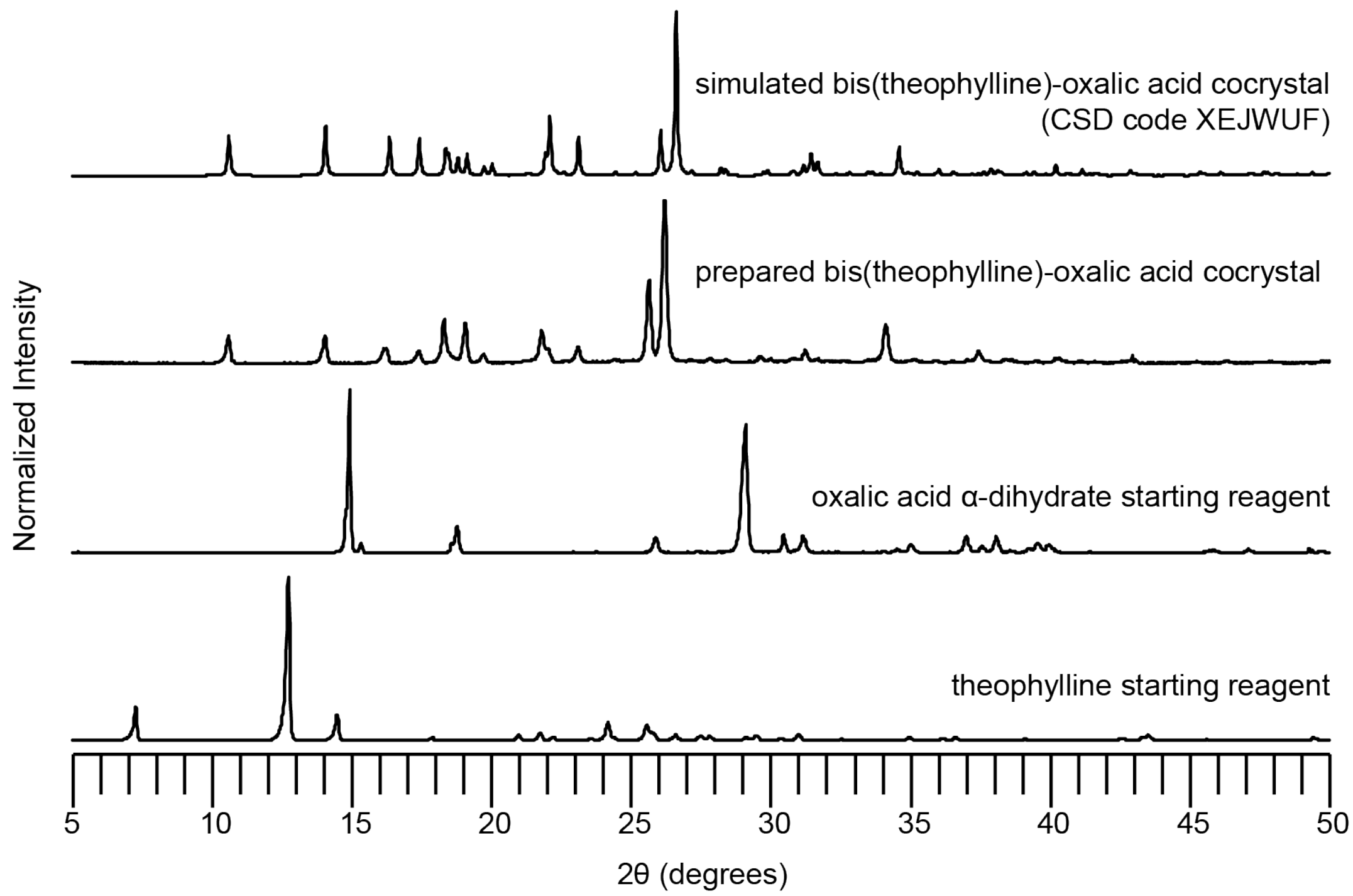

Figure S2D. PXRD pattern of bis(theophylline)–oxalic acid cocrystal. The experimental PXRD pattern of the prepared sample matches the pattern simulated from the CIF file with CSD code XEJWUF[2], corresponding to the bis(theophylline)–oxalic acid cocrystal.

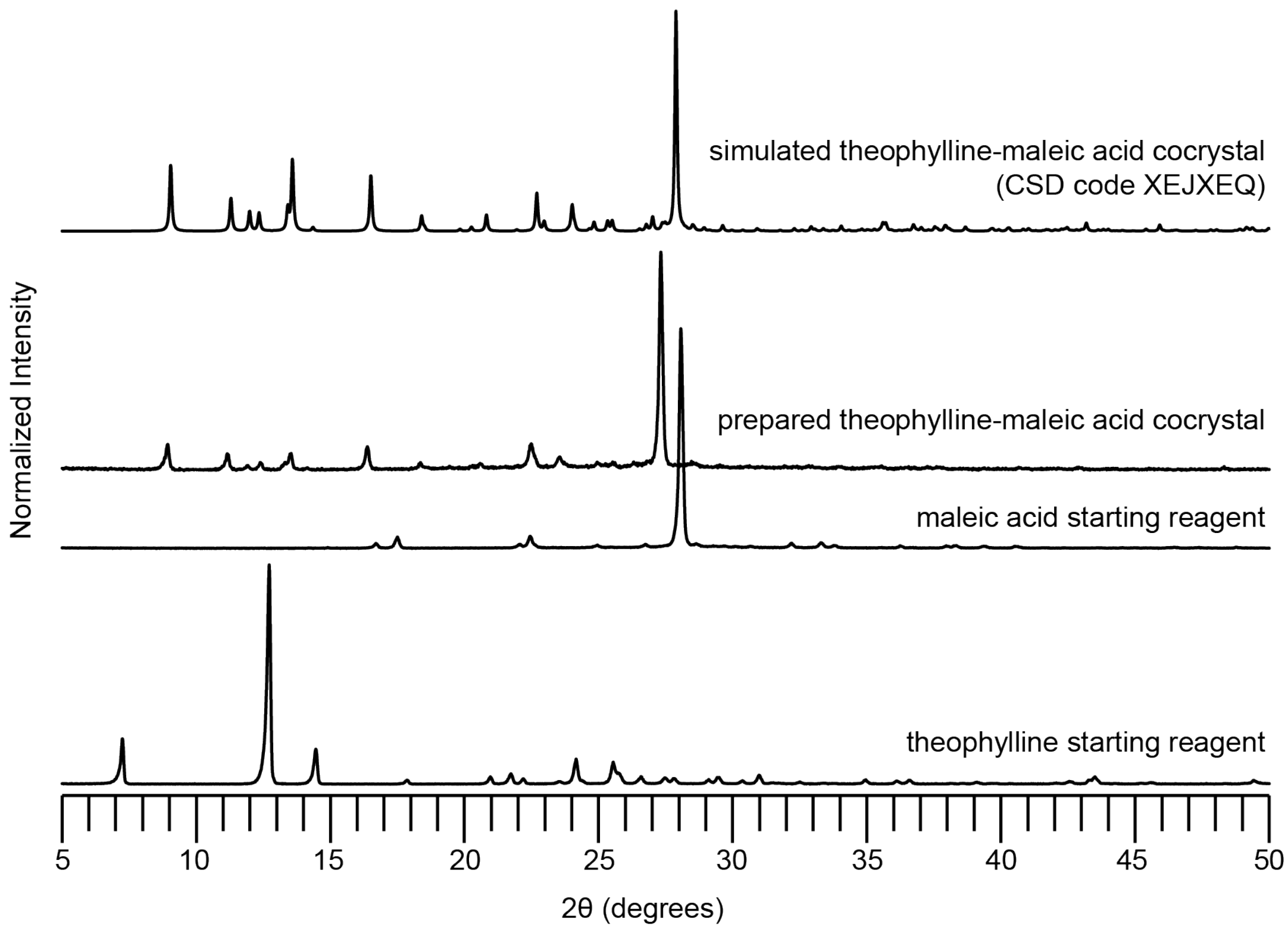

Figure S2E. PXRD pattern of the theophylline-maleic acid cocrystal. The experimental PXRD pattern of the prepared sample matches the pattern simulated from the CIF file with CSD code XEJXEQ[2], corresponding to the theophylline–maleic acid cocrystal.

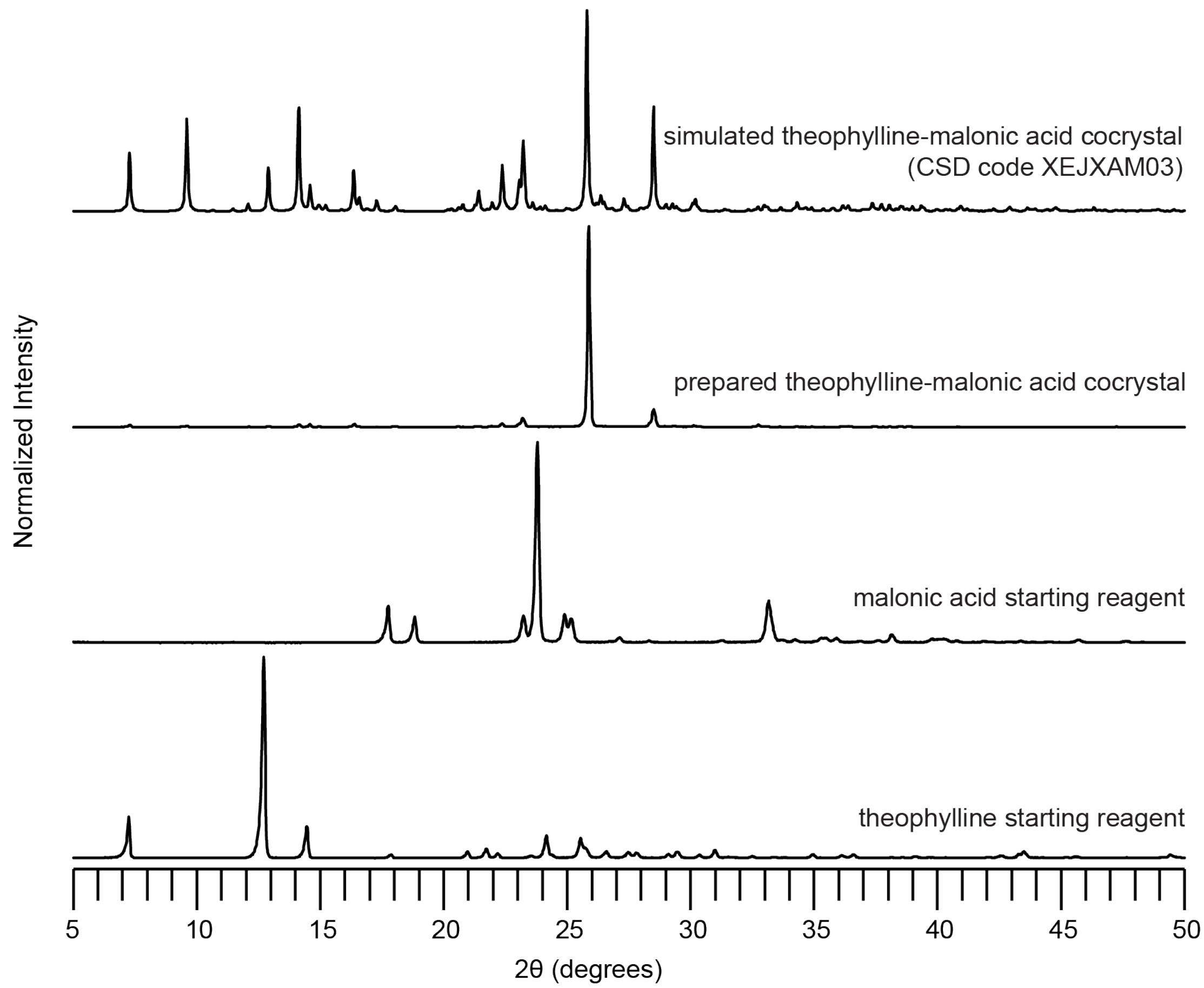

Figure S2F. PXRD pattern of the theophylline-malonic acid cocrystal. The experimental PXRD pattern of the prepared sample matches the pattern simulated from the CIF file with CSD code XEJXAM03[11], corresponding to the theophylline–malonic acid cocrystal.

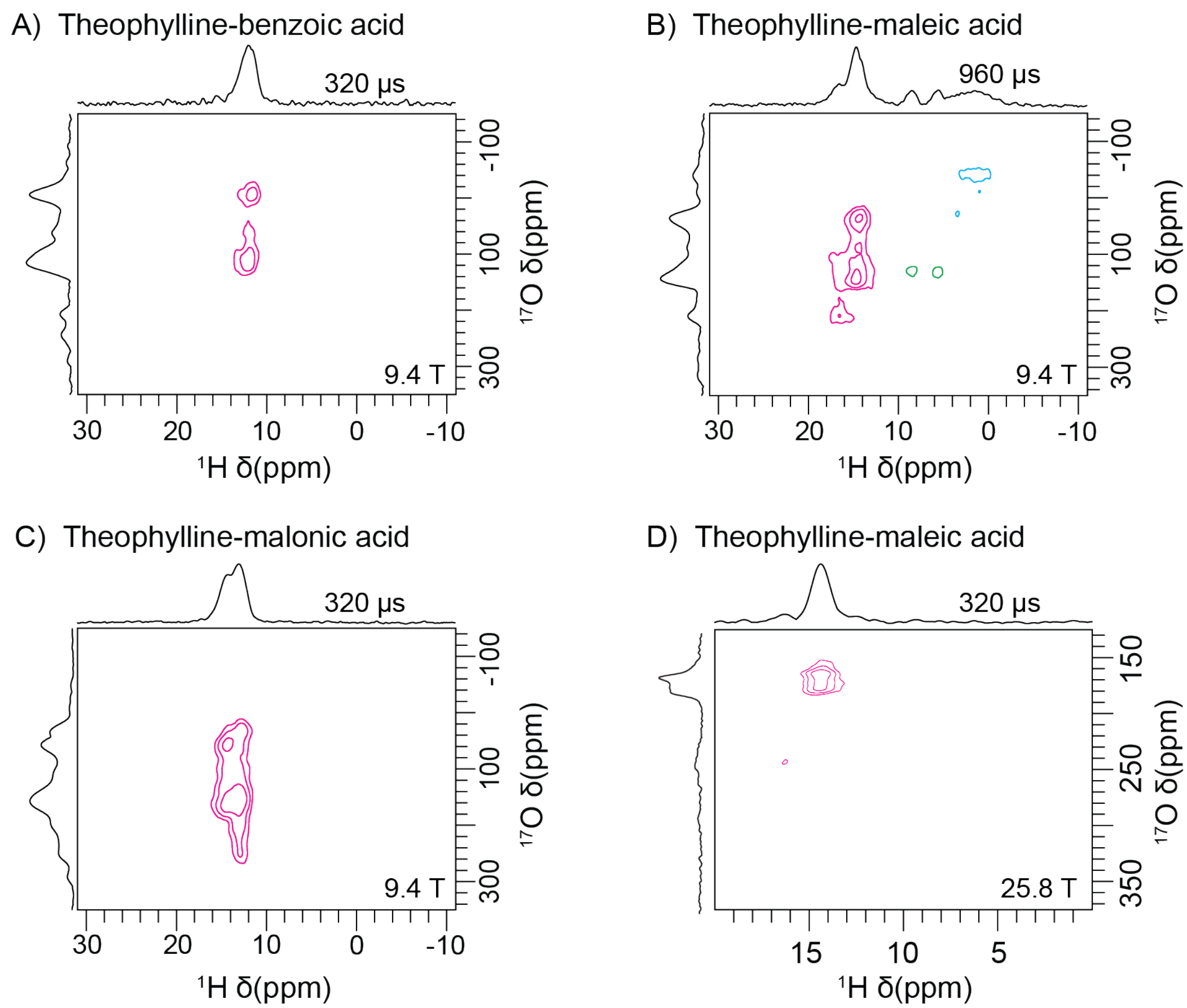

Figure S3. Two-dimensional $^{17}O \rightarrow ^{1}H$ D-RINEPT solid-state NMR spectra of theophylline cocrystals. (A) 9.4 T spectrum of theophylline–benzoic acid cocrystal with a total recoupling time of 320 μs. (B) 9.4 T spectrum of theophylline–maleic acid cocrystal with a total recoupling time of 960 μs. (C) 9.4 T spectrum of theophylline–malonic acid cocrystal with a total recoupling time of 320 μs. (D) 25.8 T spectrum of theophylline–maleic acid cocrystal acquired at an estimated sample temperature of 51 ºC with a total recoupling time of 320 μs. Spectra shown in Panels A–C were acquired with a sample temperature of approximately 40 °C, a 9.4 T magnetic field and a 50 kHz MAS frequency. Sample temperatures were estimated from the temperature-dependent ^{13}C chemical shift difference of adamantane (Table S2).[12] The spectrum in Panel D was acquired with a 25.8 T magnetic field and a 50 kHz MAS frequency.

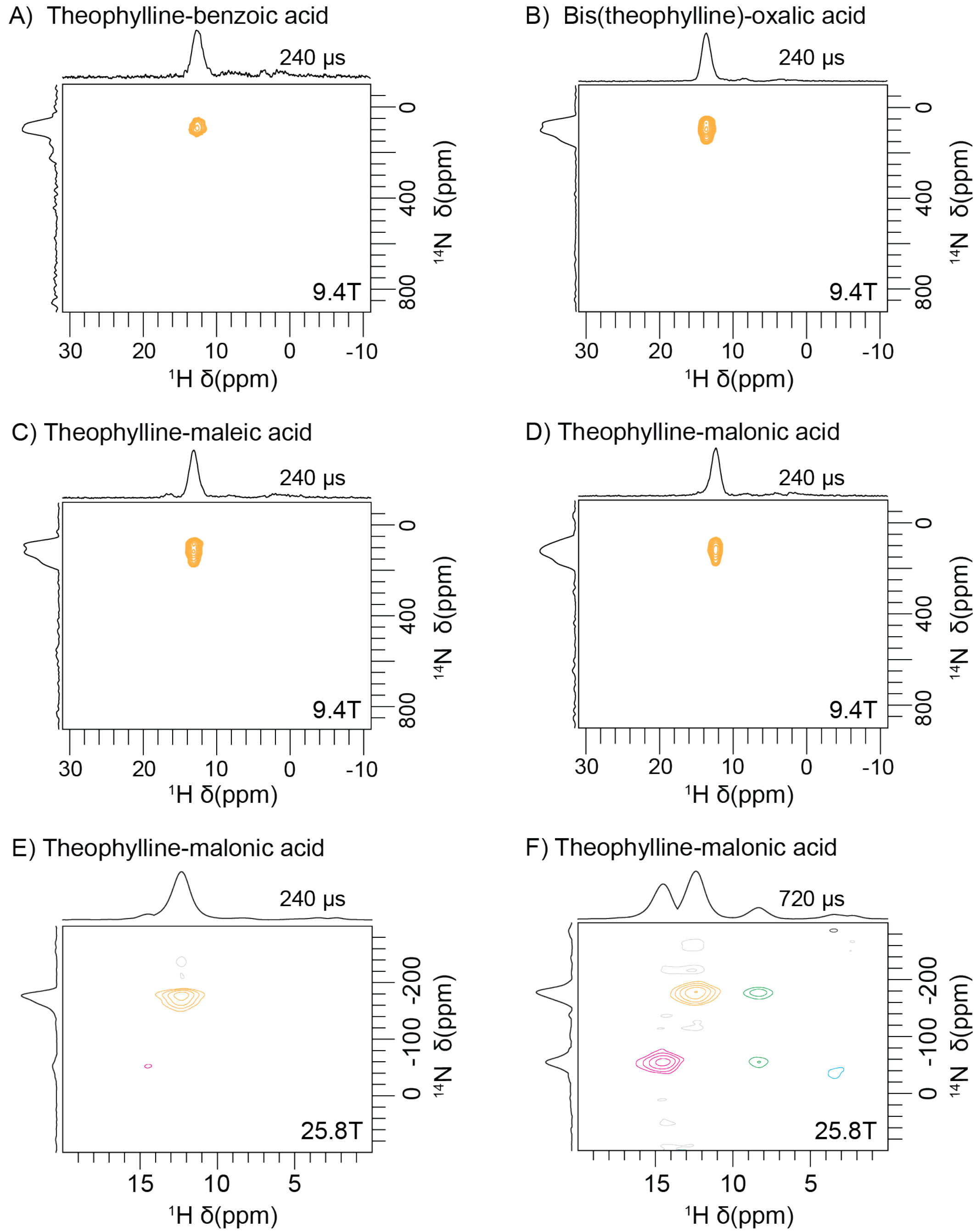

Figure S4. Two-dimensional $^{1}H\{^{14}N\}$ D-HMQC solid-state NMR spectra of theophylline cocrystals. (A) 9.4 T spectrum of theophylline–benzoic acid with a total recoupling time of 240 µs. (B) 9.4 T spectrum of bis(theophylline)–oxalic acid with a total recoupling time of 240 µs. (C) 9.4 T spectrum of theophylline–maleic acid with a total recoupling time of 240 µs. (D) 9.4 T spectrum of theophylline–malonic acid with a total recoupling time of 240 µs. (E) 25.8 T spectrum of theophylline–malonic acid with a total recoupling time of 320 µs. (F) 25.8 T spectrum of theophylline–malonic acid with a total recoupling time of 720 µs. Spectra in Panels A–D were acquired with a 9.4 T magnetic field and a 50 kHz MAS frequency. Spectra in Panels E and F were acquired with a 25.8 T magnetic field and a 50 kHz MAS frequency.

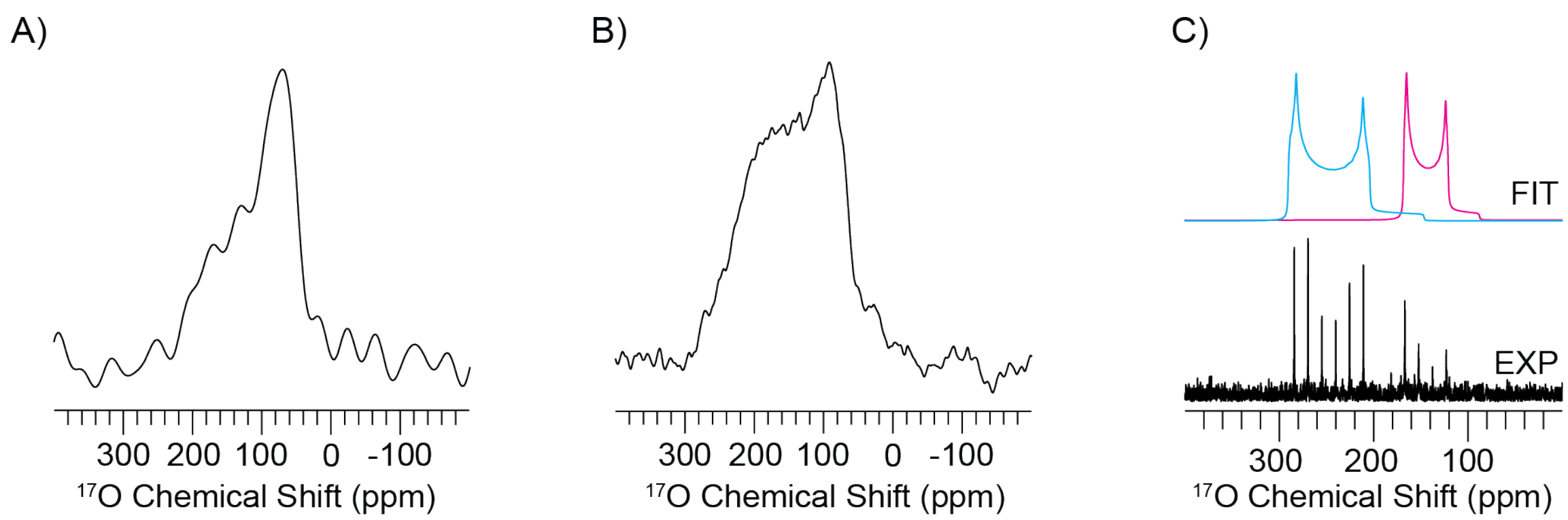

Figure S5. ^{17}O solid-state NMR spectra of the theophylline-oxalic acid cocrystal. (A) ^{17}O NMR spectrum extracted from the $^{17}O{\rightarrow}^{1}H$ D-RINEPT NMR spectrum obtained with a total recoupling time of 480 µs at 9.4 T. (B) Directly detected ^{17}O spin echo NMR spectrum obtained at 9.4 T. (C) Directly detected ^{17}O QCPMG NMR spectrum obtained at 14.1 T. Spectra in Panels A and B were acquired with a 9.4 T magnetic field and a 50 kHz MAS frequency. The spectrum in Panel C was acquired with a 14.1 T magnetic field and a 50 kHz MAS frequency.

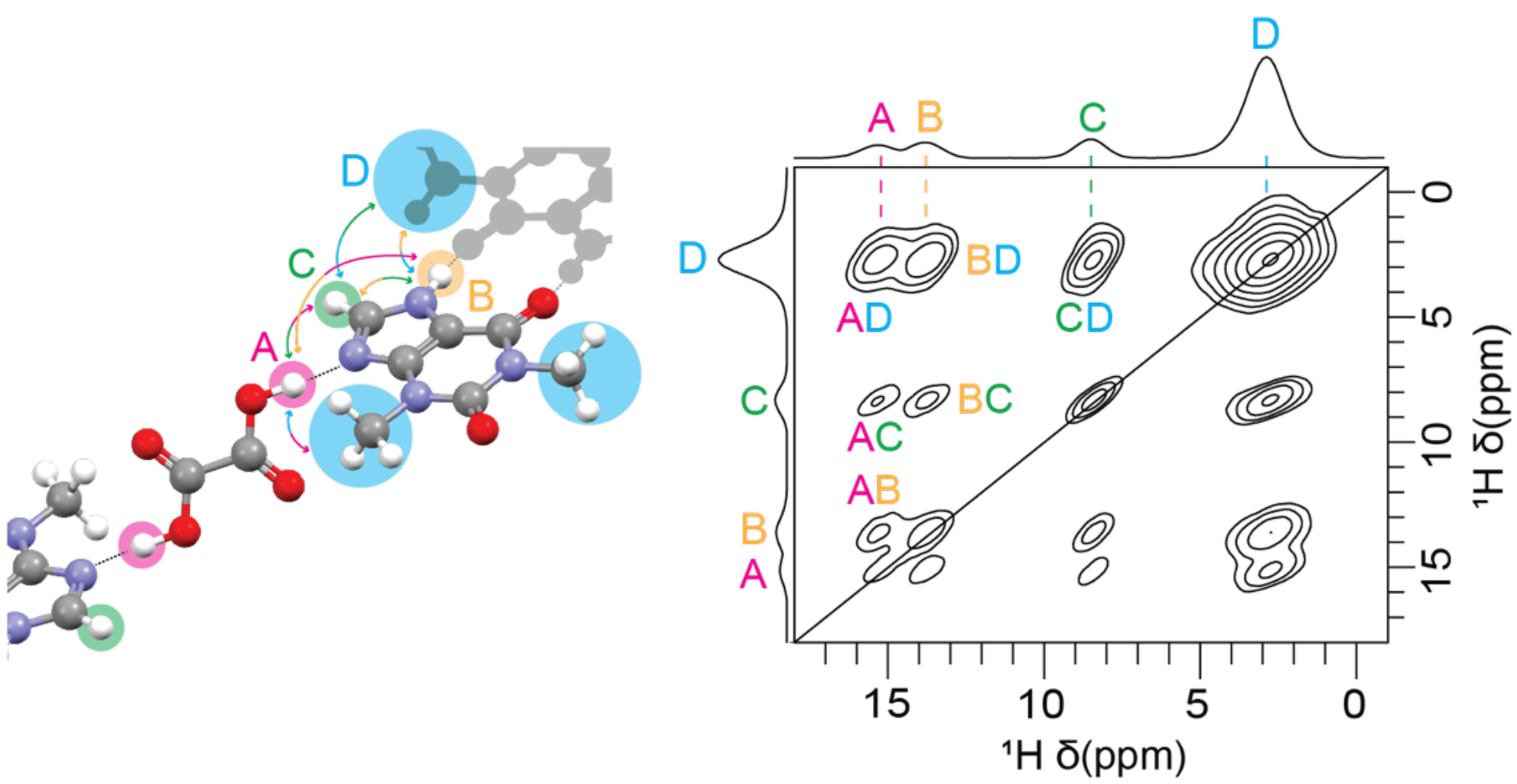

Figure S6A. Two-dimensional homonuclear ^{1}H–^{1}H spin-diffusion single-quantum–single-quantum (SQ–SQ) solid-state NMR spectrum of the bis(theophylline)–oxalic acid cocrystal acquired with a 9.4 T magnetic field, a 50 kHz MAS frequency, and a spin-diffusion mixing time of 200 ms. Proton site assignments shown in the structural diagram correspond to A (carboxylic acid OH), B (theophylline NH), C (theophylline CH), and D (theophylline CH_3) protons. Cross-peaks arise from ^{1}H–^{1}H dipolar-mediated spin diffusion between spatially proximate protons.

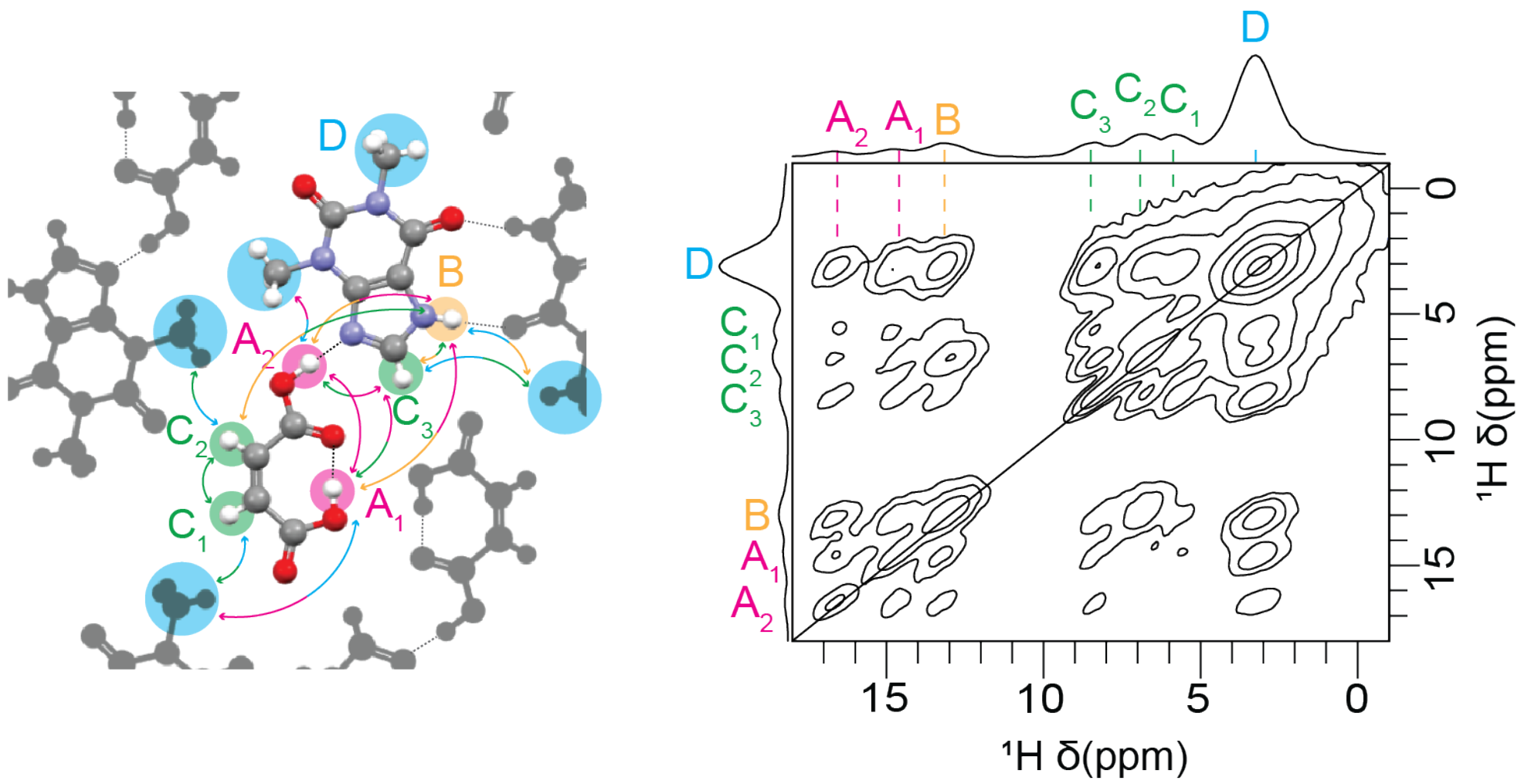

Figure S6B Two-dimensional homonuclear ^1H–^1H spin-diffusion single-quantum–single-quantum (SQ–SQ) solid-state NMR spectrum of the theophylline–maleic acid cocrystal acquired with a 9.4 T magnetic field, a 50 kHz MAS frequency, and a spin-diffusion mixing time of 200 ms. Proton site assignments shown in the structural diagram correspond to A_1/A_2 (carboxylic acid OH), B (theophylline NH), C_1/C_2 (maleic CH), C_3 (theophylline CH) and D (theophylline CH_3) protons. Cross-peaks arise from ^1H–^1H dipolar-mediated spin diffusion between spatially proximate protons.

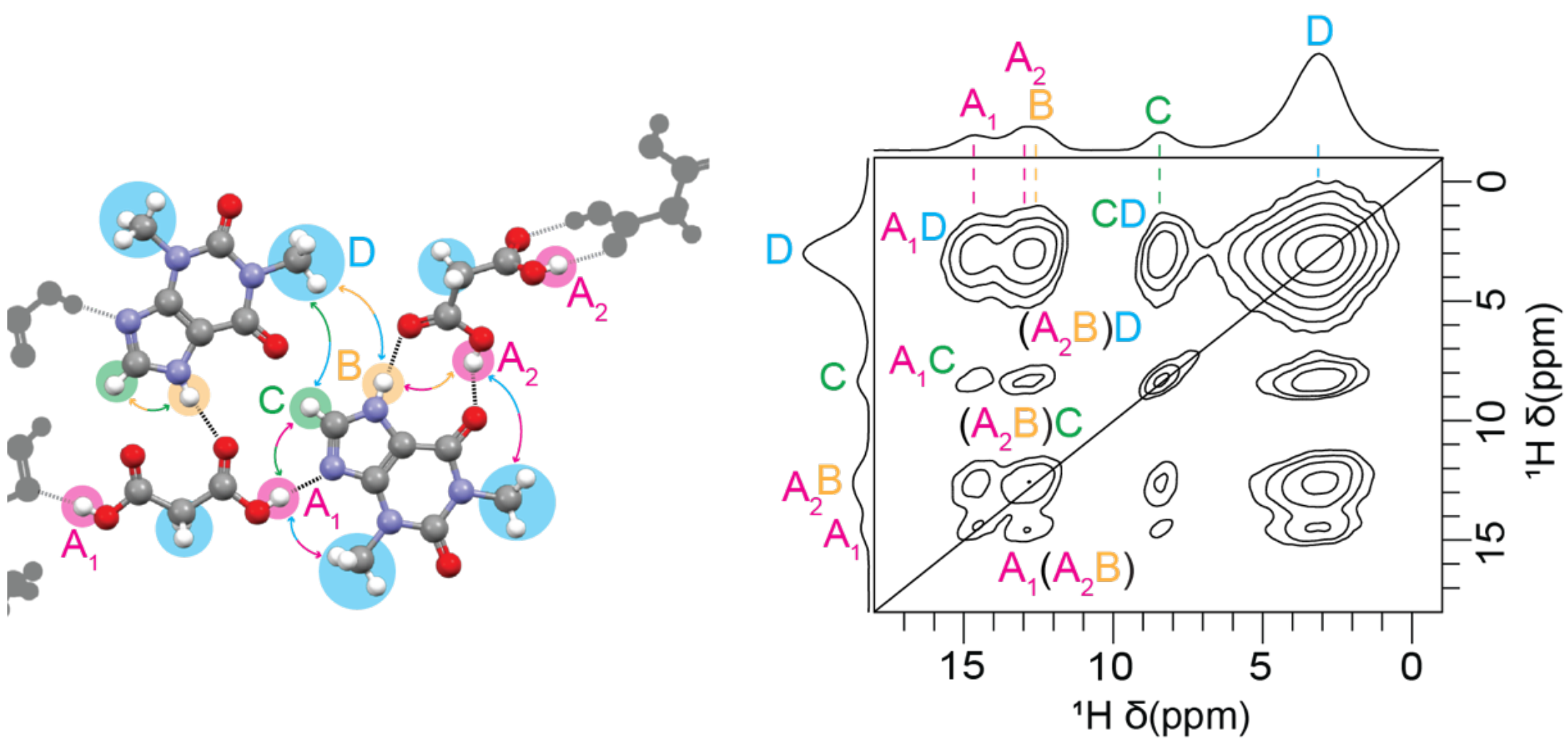

Figure S6C Two-dimensional homonuclear ^1H–^1H spin-diffusion single-quantum–single-quantum (SQ–SQ) solid-state NMR spectrum of the theophylline–malonic acid cocrystal acquired at with a 9.4 T magnetic field, a 50 kHz MAS frequency, and a spin-diffusion mixing time of 200 ms. Proton site assignments shown in the structural diagram correspond to A_1/A_2 (carboxylic acid OH), B (theophylline NH), C (theophylline CH), and D (theophylline CH_3) protons. Cross-peaks arise from ^1H–^1H dipolar-mediated spin diffusion between spatially proximate protons.

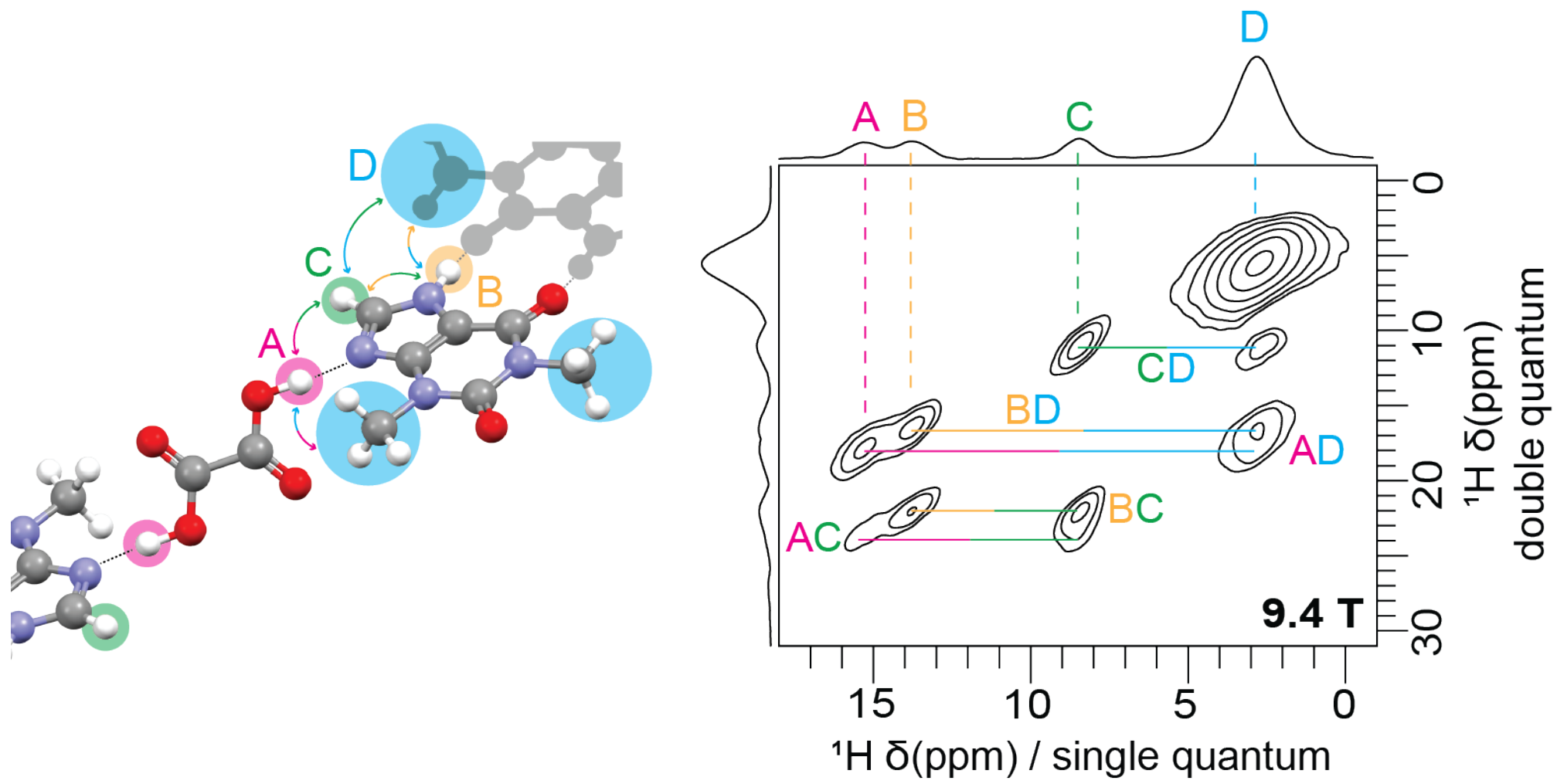

Figure S7A. Two-dimensional homonuclear ^1H–^1H single-quantum–double-quantum (SQ–DQ) solid-state NMR spectrum of the bis(theophylline)–oxalic acid cocrystal acquired with a 9.4 T magnetic field, a 50 kHz MAS, and BABA recoupling for 1 rotor period. Proton site assignments shown in the structural diagram correspond to A (carboxylic acid OH), B (theophylline NH), C (theophylline CH), and D (theophylline CH_3) protons. The SQ–DQ correlations arise from strong ^1H–^1H dipolar couplings between spatially proximate protons.

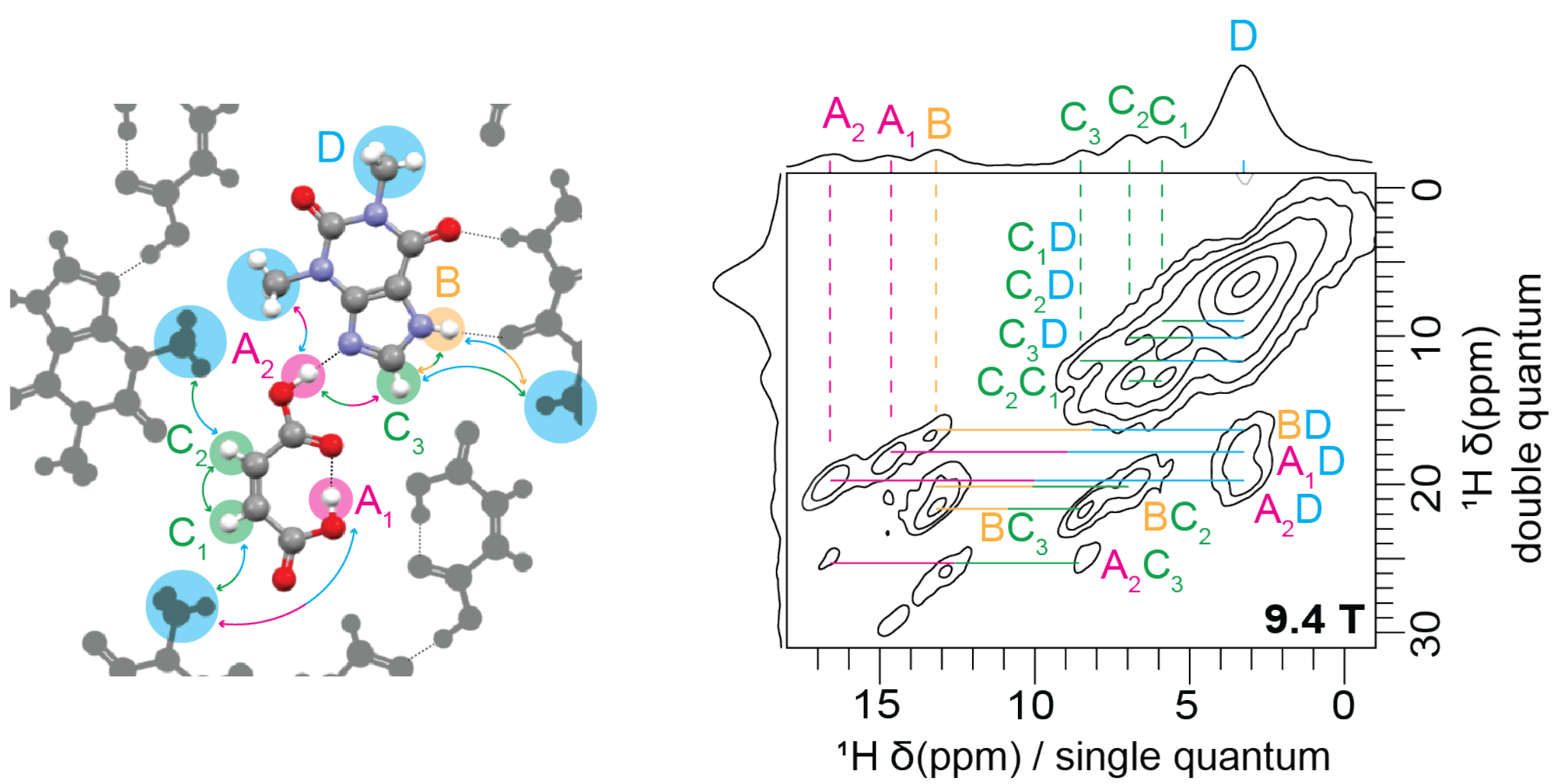

Figure S7B. Two-dimensional homonuclear ^1H–^1H single-quantum–double-quantum (SQ–DQ) solid-state NMR spectrum of the theophylline–maleic acid cocrystal acquired with a 9.4 T magnetic field, a 50 kHz MAS frequency, and BABA recoupling for 1 rotor period. Proton site assignments shown in the structural diagram A_1/A_2 (carboxylic acid OH), B (theophylline NH), C_1/C_2 (maleic CH), C_3 (theophylline CH) and D (theophylline CH_3) protons. The SQ–DQ correlations arise from strong ^1H–^1H dipolar couplings between spatially proximate protons.

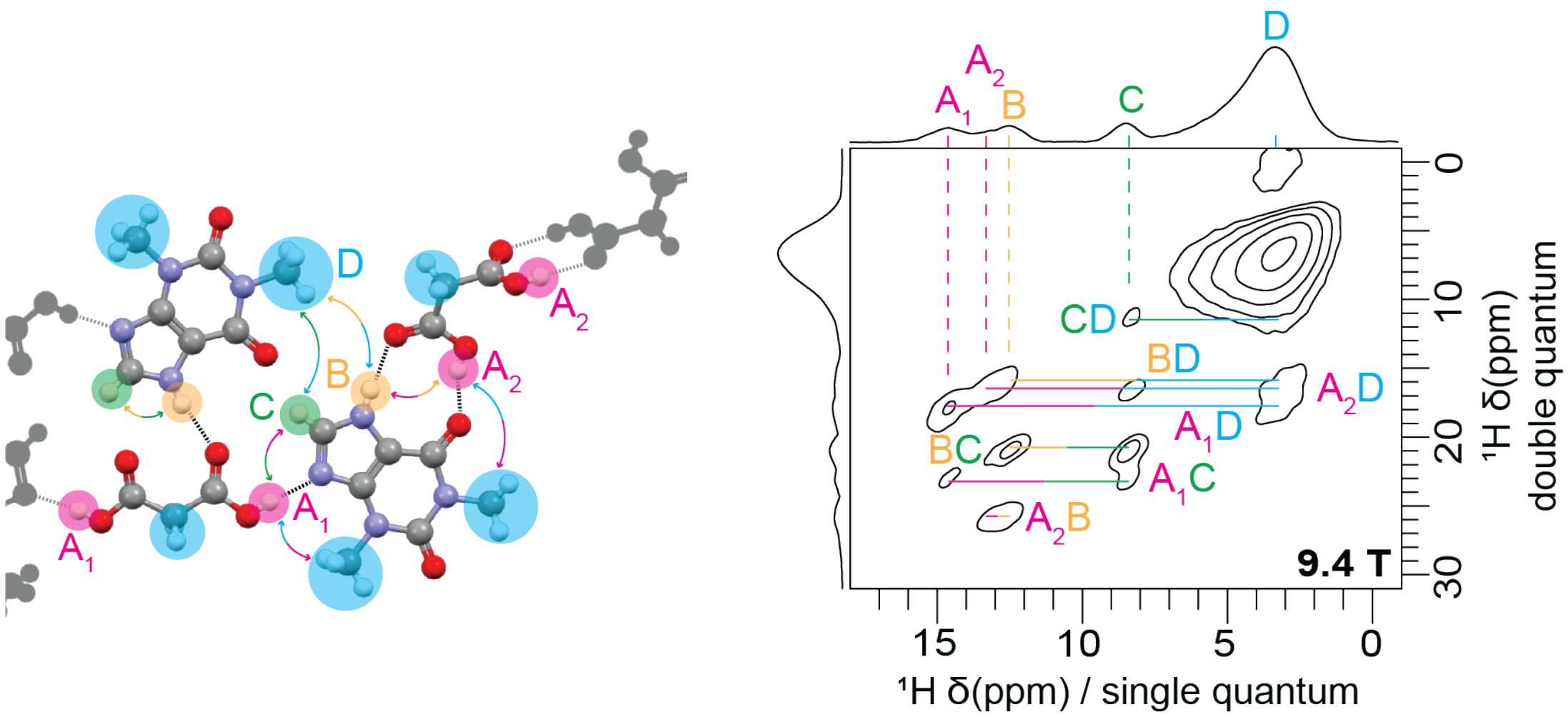

Figure S7C. Two-dimensional homonuclear ^1H–^1H single-quantum–double-quantum (SQ–DQ) solid-state NMR spectrum of the theophylline–malonic acid cocrystal acquired with a 9.4 T magnetic field, a 50 kHz MAS frequency, and BABA recoupling for 1 rotor period. Proton site assignments shown in the structural diagram correspond to A_1/A_2 (carboxylic acid OH), B (theophylline NH), C (theophylline CH), and D (theophylline CH_3) protons. The SQ–DQ correlations arise from strong ^1H–^1H dipolar couplings between spatially proximate protons.

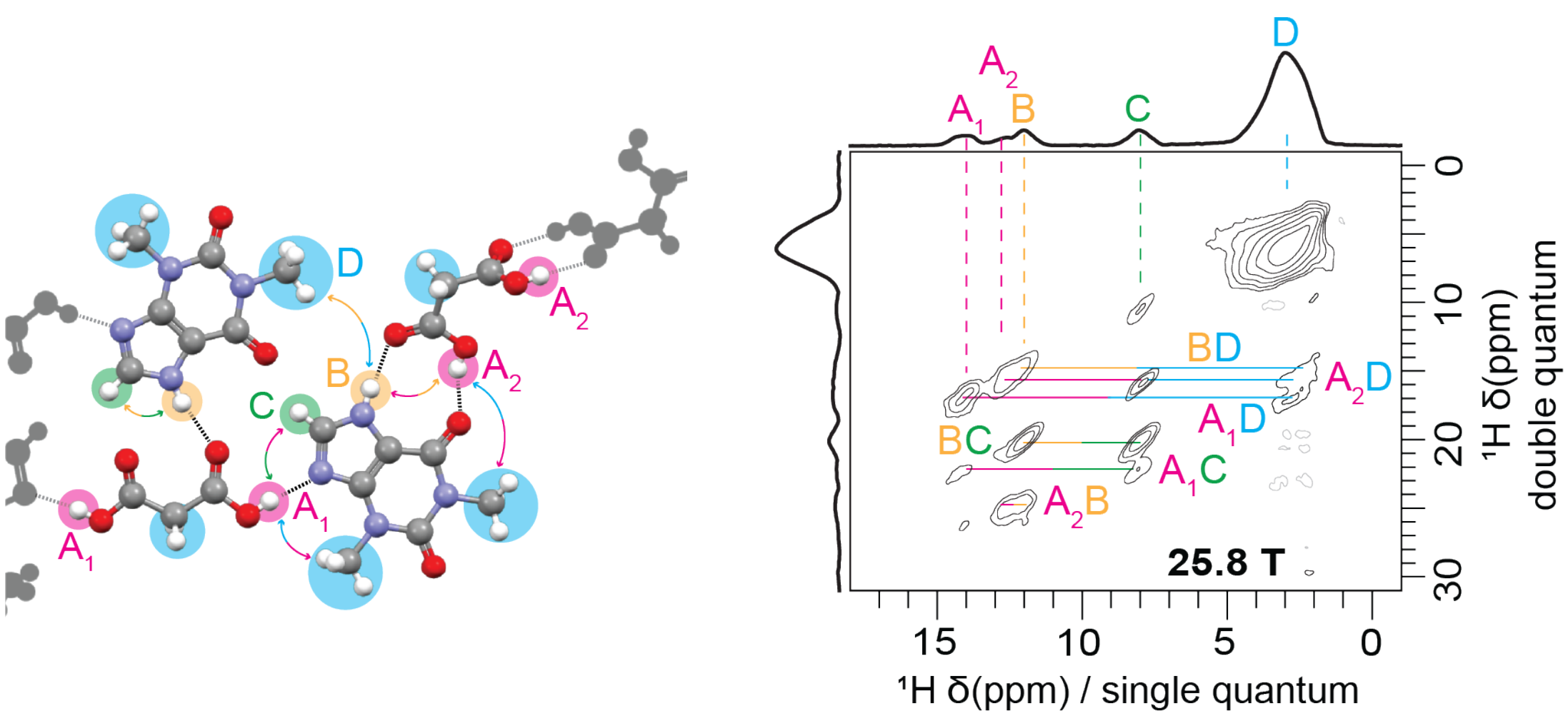

Figure S7D. Two-dimensional homonuclear ^1H–^1H single-quantum–double-quantum (SQ–DQ) solid-state NMR spectrum of the theophylline–malonic acid cocrystal acquired at with a 25.8 T magnetic field, a 50 kHz MAS frequency, and using BABA recoupling for 1 rotor period. Proton site assignments shown in the structural diagram correspond to A_1/A_2 (carboxylic acid OH), B (theophylline NH), C (theophylline CH), and D (theophylline CH_3) protons. The SQ–DQ correlations arise from strong ^1H–^1H dipolar couplings between spatially proximate protons.

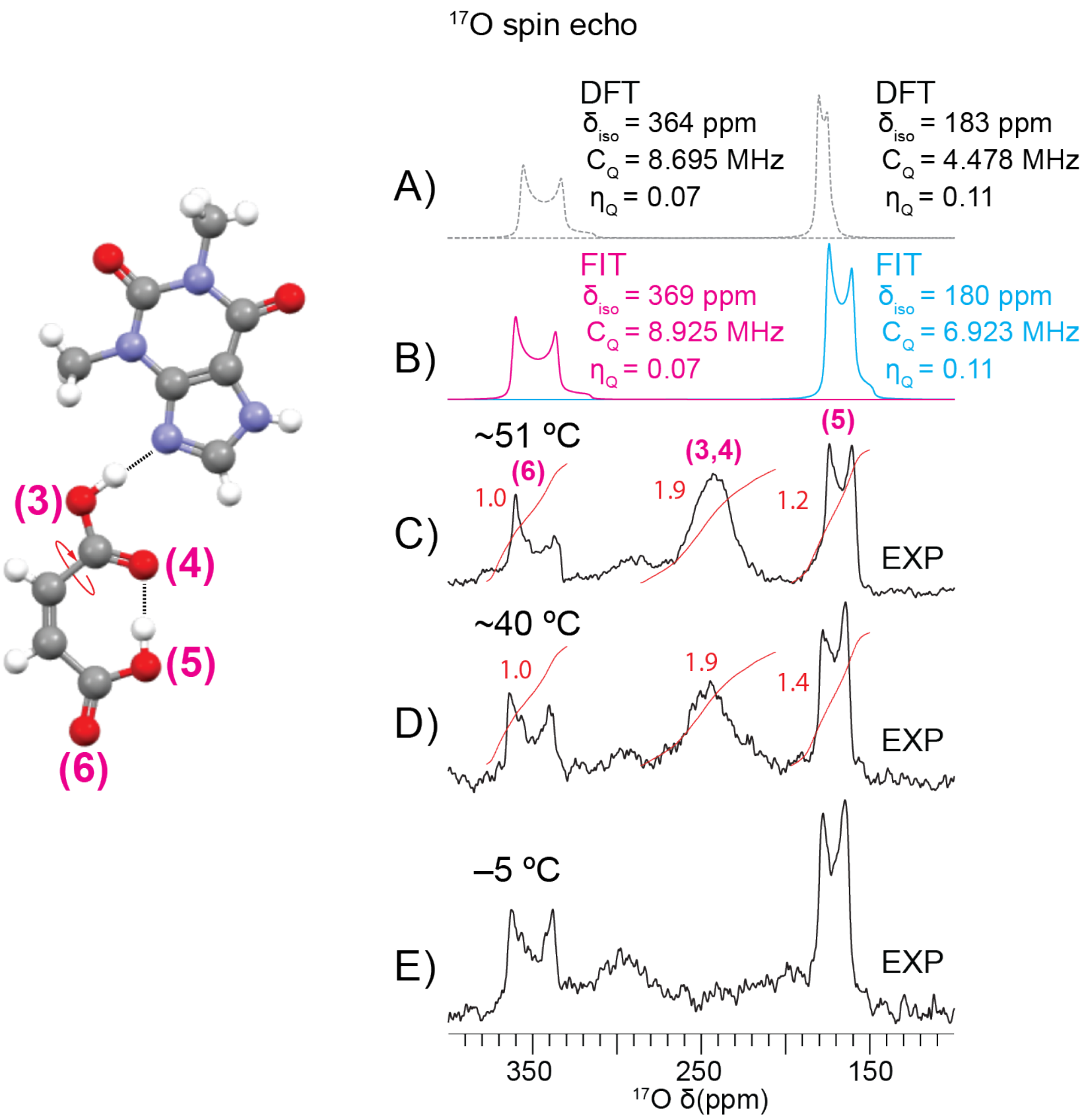

Figure S8. Experimental ^{17}O spin-echo solid-state NMR spectra of the theophylline–maleic acid cocrystal acquired with a 25.8 T magnetic field and a 50 kHz MAS frequency. Experimental NMR spectra are compared to analytically simulated NMR spectra. (A) DFT-calculated spectrum. (B) Best-fit simulation of the experimental ^{17}O spin echo spectrum. (C–E) Experimental ^{17}O spin echo spectra acquired at estimated sample temperatures of 51 ºC (C), 40 ºC (D), and –5 ºC (E). Integrals of the three spectral regions at 350, 240, and 170 ppm are 1.0, 1.9, and 1.2 at approximately 51 ºC and 1.0, 1.9 and 1.4 at approximately 40 ºC, corresponding to oxygen sites O(6), O(3,4), and O(5), respectively. All spectra were acquired using a spin echo pulse sequence with a recycle delay (d1) of 2 s. Estimated sample temperatures of approximately 51 °C and 40 °C were determined from the temperature-dependent ^{13}C chemical shift difference of adamantane (Table S2).[12] (Table S2). The –5 °C value corresponds to the variable-temperature (VT) set point.

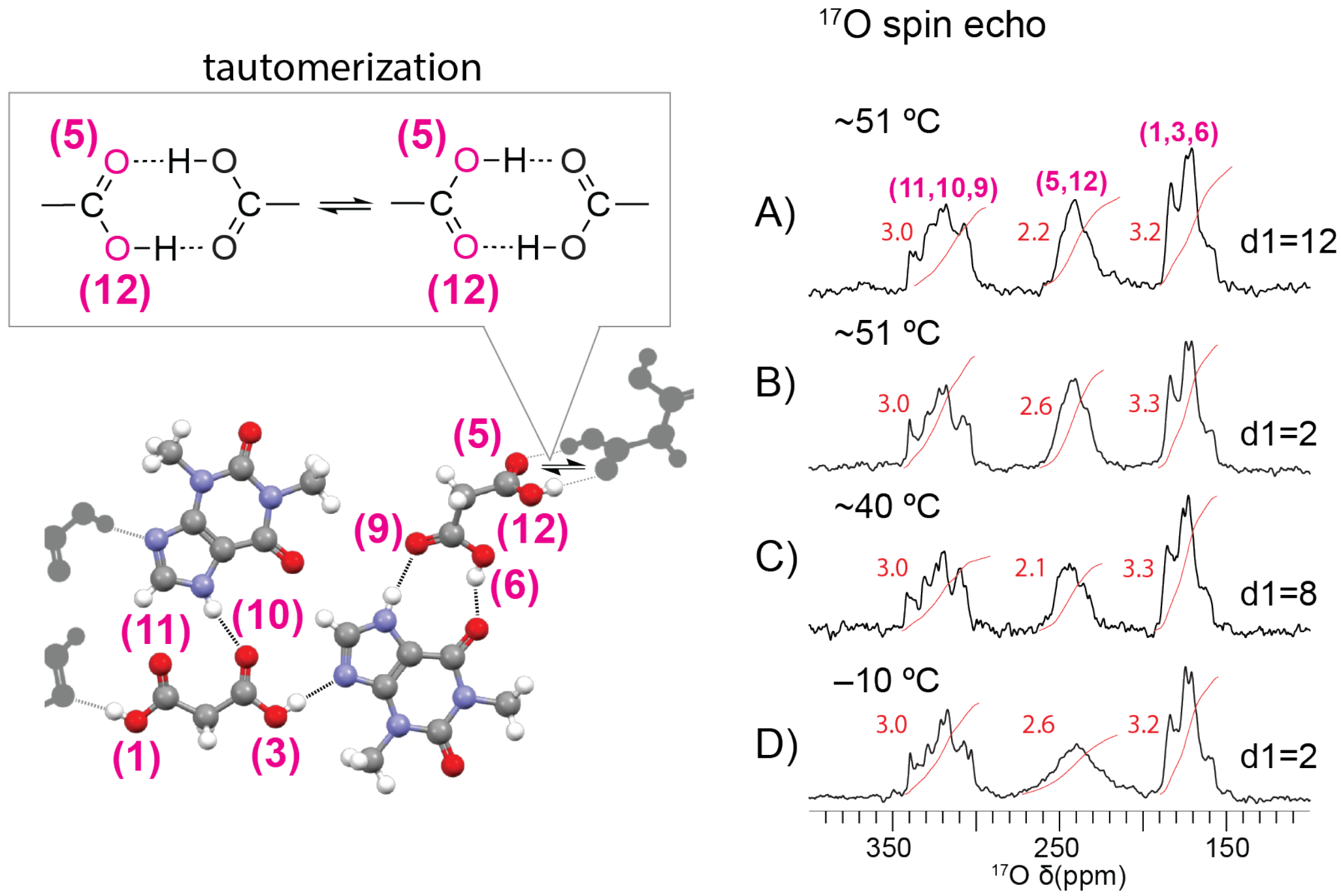

Figure S9. ^{17}O spin echo solid-state NMR spectra of the theophylline–malonic acid cocrystal acquired with a 25.8 T magnetic field and 50 kHz MAS frequency. (A) Spectrum acquired with an estimated sample temperature of 51 °C with a recycle delay (d1) of 12 s. Integrals of the three spectral regions at approximately 330, 240, and 170 ppm are 3.0, 2.2, and 3.2, respectively, corresponding to oxygen sites O(11,10,9), O(5,12), and O(1,3,6). (B) Spectrum acquired at an estimated sample temperature of approximately 51 °C with a recycle delay (d1) of 2 s, with integral ratios 3.0, 2.6, and 3.3. (C) Spectrum acquired at an estimated sample temperature of 40°C with a recycle delay (d1) of 8 s, with integral ratios 3.0, 2.1, and 3.3. (D) Spectrum acquired with an estimated sample temperature of –10 °C with a recycle delay (d1) of 2 s, with integral ratios of 3.0, 2.6, and 3.2. Differences in the integral ratios arise from variations in relaxation behavior and incomplete relaxation when shorter recycle delays are used. Estimated sample temperatures of approximately 51 °C and 40 °C were determined from the temperature-dependent ^{13}C chemical shift difference of adamantane (Table S2).[12] The –10 °C value corresponds to the variable-temperature (VT) set point.

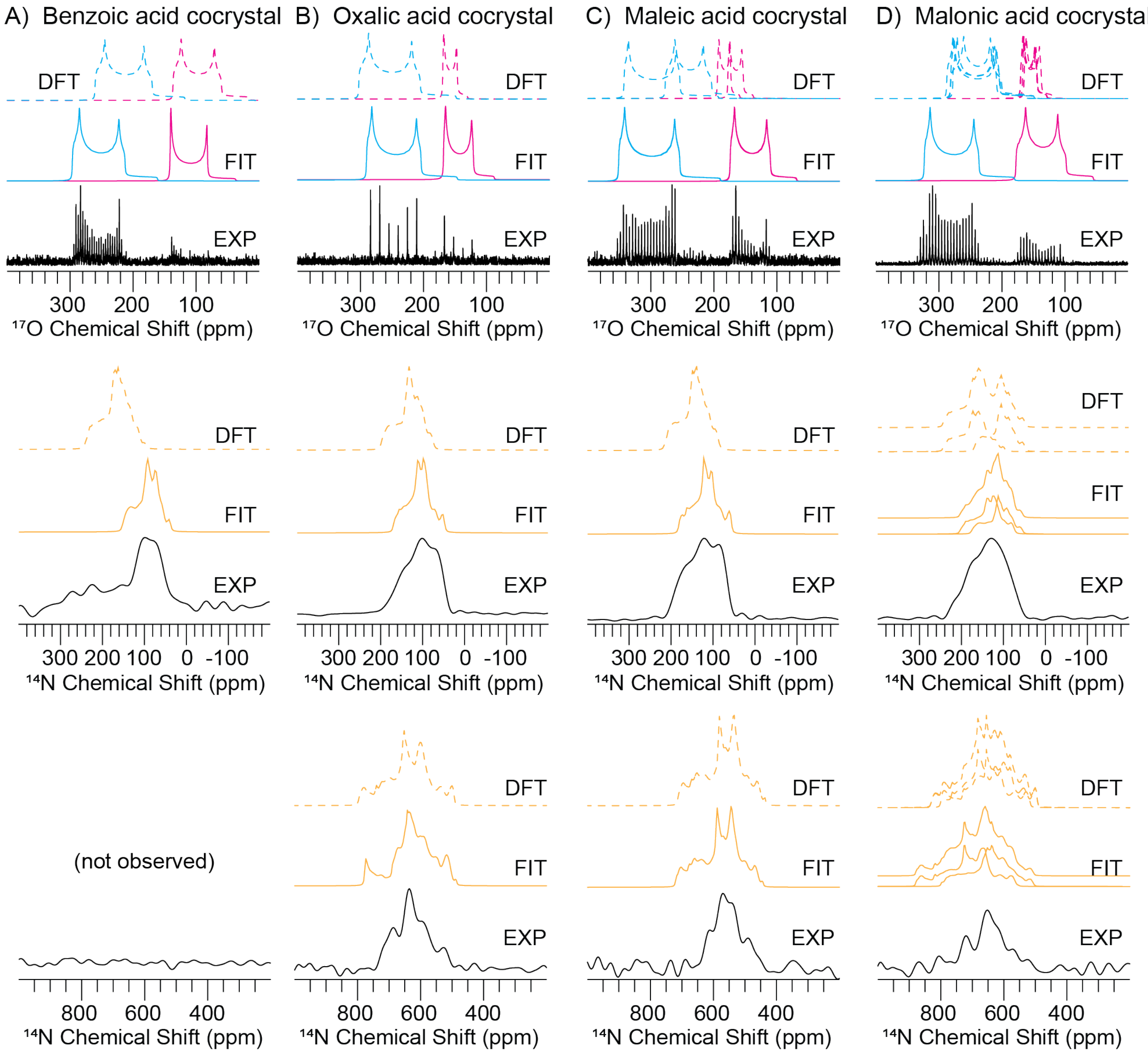

Figure S10. ^{17}O and ^{14}N SSNMR spectral fitting of (A) theophylline–benzoic acid cocrystal, (B) bis(theophylline)–oxalic acid cocrystal, (C) theophylline–maleic acid cocrystal, and (D) theophylline–malonic acid cocrystal. **Top**: ^{17}O QCPMG spectra. **Middle**: projections of the indirect dimension of two-dimensional $^{1}H\{^{14}N\}$ D-HMQC spectra acquired with a total recoupling time of 240 μs. **Bottom**: projections of the indirect dimension of two-dimensional $^{1}H\{^{14}N\}$ D-HMQC spectra acquired with a total recoupling time of 960 μs. The ^{14}N resonance centered at ca. 100 ppm corresponds to the imidazole N–H group of theophylline, whereas the resonance centered at ca. 600 ppm corresponds to the non-protonated imidazole nitrogen involved in hydrogen bonding with the carboxylic acid O–H group. The ^{17}O NMR spectra were acquired with a 14.1 T magnetic field. The $^{1}H\{^{14}N\}$ D-HMQC experiments were acquired with a 9.4 T magnetic field and 50 kHz MAS frequency. Additional experimental acquisition parameters, including MAS frequencies for the ^{17}O experiments, are provided in Table S3. All spectra were fit using the SOLA fitting module within Bruker TopSpin software.[5] DFT-calculated and experimentally fitted NMR parameters are provided in Tables S1, S4, and S5.

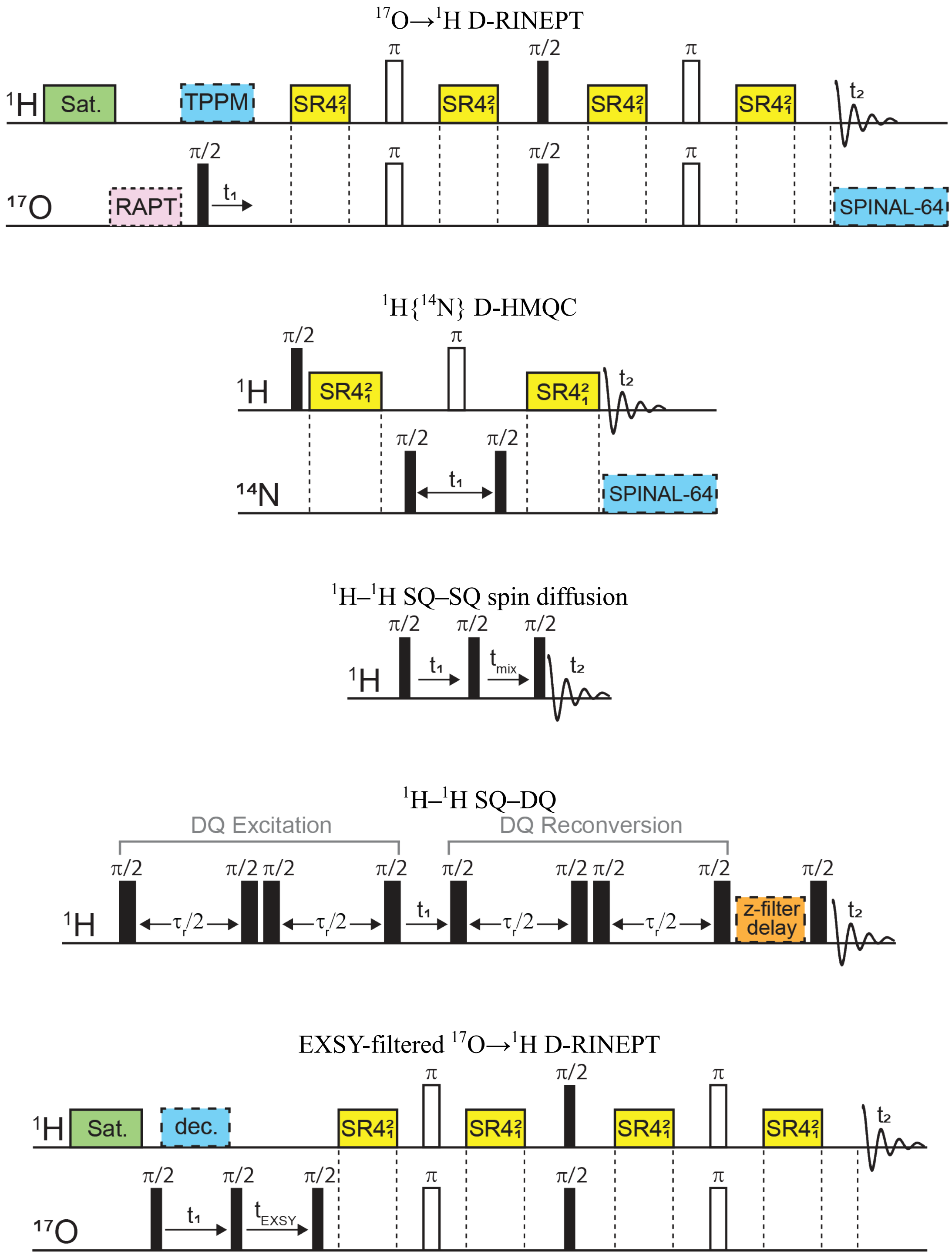

Figure S11. Solid-state NMR pulse sequences used in this work.

Table S1. Plane-wave DFT (GIPAW) calculated ^{17}O, ^{14}N, and ^{1}H NMR parameters.

**Dihydrate or Cocrystal**	**Site Number**	**σ_{iso} (ppm)**	**η_Q**	**$\|C_Q\|$ (MHz)**	**δ_{iso}[a] (ppm)**
Theophylline–benzoic acid 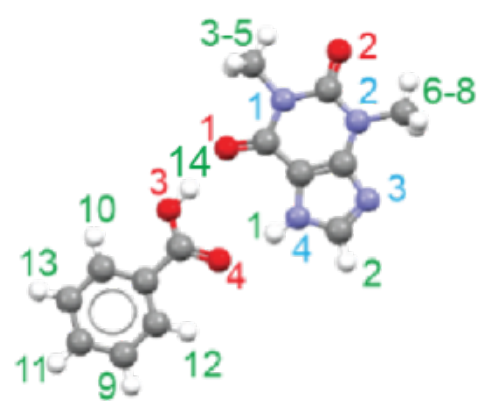	O1	–3.9	0.55	7.9	247
	O2	0.3	0.45	8.2	243
	O3	93.9	0.17	7.7	150
	O4	–32.8	0.17	8.2	276
	N1	66.6	0.09	3.5	–226
	N2	101.0	0.01	3.8	–258
	N3	–8.2	0.26	3.5	–155
	N4	64.1	0.87	1.9	–224
	H1	16.1	-	-	13.0
	H2	21.5	-	-	7.9
	H3	27.9	-	-	1.9
	H4	27.2	-	-	2.6
	H5	26.5	-	-	3.2
	H6	26.8	-	-	3.0
	H7	27.1	-	-	2.7
	H8	27.8	-	-	2.0
	H9	23.7	-	-	5.9
	H10	24.2	-	-	5.4
	H11	22.2	-	-	7.2
	H12	22.7	-	-	6.8
	H13	23.5	-	-	6.0
	H14	17.2	-	-	12.0
Bis(theophylline)–oxalic acid 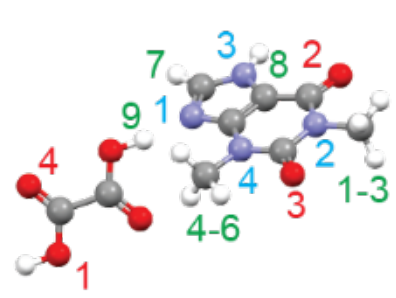	O1	69.4	0.05	7.1	175
	O2	–19.9	0.47	8.3	263
	O3	10.7	0.45	8.1	233
	O4	–76.8	0.19	9.3	320
	N1	8.4	0.6	2.8	–171
	N2	63.9	0.12	3.5	–223
	N3	65.4	0.95	1.7	–225
	N4	105.5	0.03	3.9	–263
	H1	26.7	-	-	3.0
	H2	27.7	-	-	2.1
	H3	28.9	-	-	0.9
	H4	28.1	-	-	1.7
	H5	27.3	-	-	2.4
	H6	27.9	-	-	2.0
	H7	22.0	-	-	7.4
	H8	15.8	-	-	13.2
	H9	14.7	-	-	14.3
Theophylline–maleic acid	O1	–21.1	0.43	8.5	264
	O2	3.3	0.38	8.1	240
	O3	45.4	0.09	6.8	198
	O4	–44.9	0.26	7.6	288
	O5	61.0	0.11	7.2	183
	O6	–121.7	0.06	9.3	364
	N1	68.4	0.88	1.8	–228

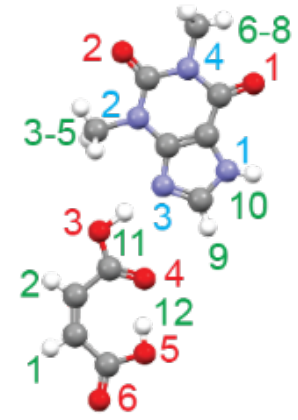	N2	104.9	0.02	3.9	–262
	N3	13.3	0.67	2.7	–176
	N4	63.0	0.1	3.4	–223
	N5	68.4	0.88	1.8	–228
	N6	104.9	0.02	3.9	–262
	N7	13.3	0.67	2.7	–176
	N8	63.0	0.1	3.4	–223
	H1	25.1	-	-	4.6
	H2	23.7	-	-	5.9
	H3	27.2	-	-	2.6
	H4	28.3	-	-	1.5
	H5	26.4	-	-	3.3
	H6	28.4	-	-	1.5
	H7	27.9	-	-	2.0
	H8	25.1	-	-	4.5
	H9	22.2	-	-	7.3
	H10	16.4	-	-	12.7
	H11	13.2	-	-	15.7
	H12	14.9	-	-	14.1
Theophylline–malonic acid 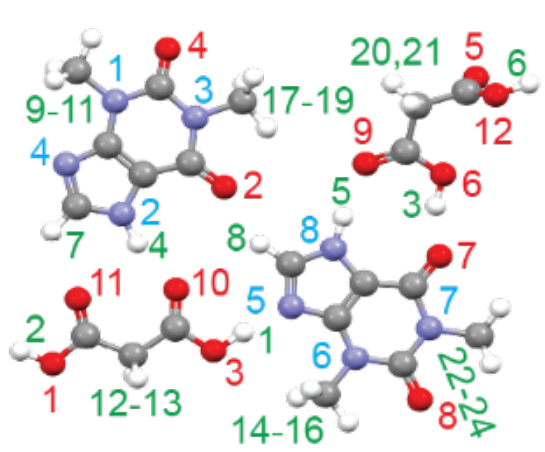	O1	69.2	0.14	7.2	175
	O2	–33.4	0.26	8.6	277
	O3	68.8	0.2	7.2	175
	O4	2.3	0.37	8.1	241
	O5	–47.3	0.37	7.7	290
	O6	73.5	0.13	7.4	170
	O7	–4.6	0.52	7.9	248
	O8	5.2	0.36	8.0	238
	O9	–49.8	0.09	7.9	293
	O10	–57.5	0.04	8.6	300
	O11	–62.4	0.12	8.8	305
	O12	71.6	0.09	7.0	172
	N1	106.8	0.04	3.9	–264
	N2	68.3	0.93	1.7	–227
	N3	67.5	0.11	3.5	–227
	N4	14.5	0.49	2.9	–177
	N5	14.5	0.54	2.9	–177
	N6	107.7	0.01	3.9	–265
	N7	69.1	0.11	3.5	–228
	N8	70.5	0.83	1.9	–230
	H1	14.8	-	-	14.2
	H2	15.7	-	-	13.4
	H3	16.5	-	-	12.6
	H4	17.4	-	-	11.8
	H5	17.6	-	-	11.6
	H6	15.2	-	-	13.8
	H7	22.3	-	-	7.2
	H8	22.1	-	-	7.3
	H9	27.9	-	-	1.9
	H10	25.9	-	-	3.8
	H11	26.9	-	-	2.9
	H12	26.2	-	-	3.5

	H13	27.0	-	-	2.7
	H14	28.2	-	-	1.6
	H15	27.7	-	-	2.1
	H16	26.8	-	-	3.0
	H17	25.8	-	-	3.9
	H18	28.3	-	-	1.6
	H19	26.9	-	-	2.9
	H20	27.4	-	-	2.4
	H21	27.3	-	-	2.4
	H22	28.5	-	-	1.3
	H23	28.3	-	-	1.5
	H24	27.2	-	-	2.5
Oxalic acid α-dihydrate	O1	62.5	0.27	6.8	181
	O2	–33.5	0.07	8.1	277
	O3	262.2	0.7	8.3	–17
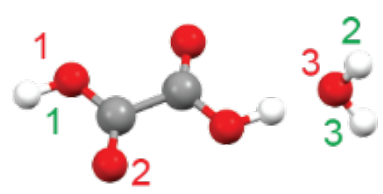	H1	12.6	-	-	16.2
	H2	24.2	-	-	5.4
	H3	25.1	-	-	4.6

(a) Previously reported calibration relationships[13,14] were used to convert DFT calculated isotropic shielding values (σ_{iso}) to isotropic shift values (δ_{iso}) for ^{17}O, ^{14}N, and ^{1}H values: $\delta_{iso}(^{17}O) = (-0.9918)(\sigma_{iso}) + 243.39$; $\delta_{iso}(^{14/15}N) = (-0.9421)(\sigma_{iso}) - 163.19$; $\delta_{iso}(^{1}H) = (-0.9374)(\sigma_{iso}) + 28.067$. The calibration relationships were derived using CASTEP/GIPAW calculations with the PBE exchange-correlation functional, Tkatchenko–Scheffler dispersion correction, and a 630 eV plane-wave cutoff energy. The present calculations employed the same computational methodology and cutoff energy, differing only in the CASTEP version and geometry optimization protocol.

Table S2. Experimental ^{13}C chemical shifts recorded at 25.8 T for adamantane under variable-temperature (VT) conditions

VT condition (ºC)	MAS rate (kHz)	^{13}C peak 1 chemical shift (ppm)	^{13}C peak 2 chemical shift (ppm)	Difference between chemical shifts (ppm)	Estimated Temperature (ºC)[a]
25	30	37.8785	28.8273	9.0512	28.0
25	40	37.8775	28.8218	9.0557	31.1
25	50	37.8742	28.8105	9.0637	36.6
35	50	37.8804	28.8073	9.0731	43.1
45	50	37.8872	28.8025	9.0847	51.0
ambient (no flow)	50	37.8581	28.789	9.0691	40.3

(a) Temperatures were estimated using the formula[12] T (ºC) = –7.15 + (686.9) × (Δ – 9) – (0.72) × (Δ – 9)^2, where T is the estimated sample temperature in ºC and Δ is the difference between chemical shifts in ppm. This method accounts for sample heating arising from rotational friction during magic-angle spinning (MAS).

Table S3. Comparison between 25.8 T experimental and DFT calculated ^{17}O SSNMR parameters

Compound / ^{17}O site	Experimental Fit			DFT calculated[a]			
	δ_{iso} (ppm)	C_Q (MHz)	η_Q	δ_{iso} (ppm)	$\|C_Q\|$ (MHz)	η_Q	CSD code
theophylline–maleic acid / O3				198	6.8	0.09	XEJXEQ
theophylline–maleic acid / O5	180	6.9	0.11	183	7.2	0.11	
theophylline–maleic acid / O4				288	7.6	0.26	
theophylline–maleic acid / O6	369	8.9	0.07	364	9.3	0.06	
theophylline–malonic acid / O1	188	5.9	0.20	175	7.2	0.14	XEJXAM03
theophylline–malonic acid / O3	177	4.5	0.30	174	7.2	0.20	
theophylline–malonic acid / O6	175	6.3	0.10	170	7.4	0.13	
theophylline–malonic acid / O12				172	7.0	0.09	
theophylline–malonic acid / O5				290	7.7	0.37	
theophylline–malonic acid / O9	328	7.6	0.09	293	8.0	0.09	
theophylline–malonic acid / O10	336	8.3	0.10	300	8.6	0.04	
theophylline–malonic acid / O11	347	8.5	0.05	305	8.8	0.12	

(a) The following equation derived from a previous calibration plot[13] was used to convert ^{17}O DFT calculated isotropic chemical shielding values (σ_{iso}) to isotropic chemical shift values (δ_{iso}): $\delta_{iso}(^{17}O) = (-0.9918)(\sigma_{iso}) + 243.39$.

Table S4. Comparison between 14.1 T experimental and DFT calculated ^{17}O SSNMR parameters

Compound / ^{17}O site	Experimental Fit			DFT calculated[a]			
	δ_{iso} (ppm)	C_Q (MHz)	η_Q	δ_{iso} (ppm)	$\lvert C_Q \rvert$ (MHz)	η_Q	CSD code
oxalic acid dihydrate / O1	189	6.3	0.11	181	6.8	0.27	OXACDH04
oxalic acid dihydrate / O2	286	7.2	0.20	277	8.1	0.07	
theophylline–benzoic acid / O3	158	7.3	0.01	150	7.7	0.17	VAXSAQ01
theophylline–benzoic acid / O4	313	8.2	0.13	276	8.2	0.17	
bis(theophylline)–oxalic acid / O1	180	6.4	0.06	175	7.1	0.05	XEJWUF
bis(theophylline)–oxalic acid / O4	310	8.5	0.09	320	9.3	0.19	
theophylline–maleic acid / O3	188	7.3	0.1	198	6.8	0.09	XEJXEQ
theophylline–maleic acid / O5				183	7.2	0.11	
theophylline–maleic acid / O4	373	9.0	0.09	288	7.6	0.26	
theophylline–maleic acid / O6				364	9.3	0.06	
theophylline–malonic acid / O1	190	7.7	0.20	175	7.2	0.14	XEJXAM03
theophylline–malonic acid / O3				175	7.2	0.20	
theophylline–malonic acid / O6				170	7.4	0.13	
theophylline–malonic acid / O12				172	7.0	0.09	
theophylline–malonic acid / O5	342	8.5	0.10	290	7.7	0.37	
theophylline–malonic acid / O9				293	8.0	0.09	
theophylline–malonic acid / O10				300	8.6	0.04	
theophylline–malonic acid / O11				305	8.8	0.12	

(a) The following equation derived from a previous calibration plot[13] were used to convert ^{17}O DFT calculated isotropic chemical shielding values (σ_{iso}) to isotropic chemical shift values (δ_{iso}): $\delta_{iso}(^{17}O) = (-0.9918)(\sigma_{iso}) + 243.39$.

Table S5. Comparison between 14.1 T experimental and DFT calculated ^{14}N SSNMR parameters

Compound / ^{14}N site	Experimental Fit			DFT calculated[c]			
	δ_{iso} (ppm)	C_Q (MHz)	η_Q	δ_{iso} (ppm)	$\lvert C_Q \rvert$ (MHz)	η_Q	CSD code
theophylline–benzoic acid / N4	–225	1.6	1.0	–224	1.9	0.87	VAXSAQ01
bis(theophylline)–oxalic acid / N3	–224<br>–222[a]	1.7	0.77	–225	1.7	0.95	XEJWUF
bis(theophylline)–oxalic acid / N1	–170<br>–165[a]	2.9	0.32	–171	2.8	0.60	
theophylline–maleic acid / N1	–222<br>–223[a]<br>–221[b]	1.8	0.69	–228	1.8	0.88	XEJXEQ
theophylline–maleic acid / N3	–167<br>–168[a]<br>–160[b]	2.7	0.67	–176	2.7	0.67	
theophylline–malonic acid / N8	–230	1.8	0.80	–230	1.9	0.83	XEJXAM03
theophylline–malonic acid / N2	–228<br>–225[a]<br>–223[b]	1.7	0.98	–227	1.7	0.93	
theophylline–malonic acid / N5	–166	2.9	0.60	–177	2.9	0.54	
theophylline–malonic acid / N4	–166<br>–167[a]<br>–165[b]	2.7	0.90	–177	2.9	0.49	

(a) ^{15}N NMR experimental reported values by Stevens *et al.*[15] and (b) Pindelska *et al.*[16] (c) The following equation derived from a previous calibration plot[14] was used to convert ^{14}N DFT calculated isotropic chemical shielding values (σ_{iso}) to isotropic chemical shift values (δ_{iso}): $\delta_{iso}(^{14/15}N) = (-0.9421)(\sigma_{iso}) - 163.19$.

Table S6. Experimental NMR parameters.

Figure	NMR Frequency (MHz)	Expt.	Transmitter Offset (ppm)	Spectral Width (ppm)	$\tau_{rec.}$ delay (s)	# of Scans	TD Points	AQ (sec)	Line Broadening (Hz)
1A oxalic	14.1 T 81.4 (^{17}O)	^{17}O spin echo	250 (acid) 0 (water)	1001	0.4	256	32768	0.2009771	600
1B maleic	14.1 T 81.4 (^{17}O)	^{17}O spin echo	260 (acid) 0 (water)	1001	0.4	256	32768	0.2009771	600
1C D-malic	14.1 T 81.4 (^{17}O)	^{17}O spin echo	250 (acid) 0 (water)	1001	0.4	256	32768	0.2009771	600
1D malonic	14.1 T 81.4 (^{17}O)	^{17}O spin echo	260 (acid) 0 (water)	1001	0.4	256	32768	0.2009771	600
1E oxalic acid dihydrate	14.1 T 81.4 (^{17}O)	^{17}O spin echo	250	1001	0.4	256	32768	0.2009771	300
1F oxalic acid dihydrate (1280 µs)	14.1 T 600.2 (^{1}H) 81.4 (^{17}O)	2D ^{17}O$\rightarrow$^{1}H D-RINEPT (50 kHz MAS)	9.5 (^{1}H) 196 (^{17}O)	833 (F2) 1229 (F1)	0.4	512	8192 (F2) 128 (F1)	0.0081920 (F2) 0.0006399 (F1)	200
1F / S1B oxalic acid dihydrate (160 µs)	14.1 T 600.2 (^{1}H) 81.4 (^{17}O)	2D ^{17}O$\rightarrow$^{1}H D-RINEPT (50 kHz MAS)	9.5 (^{1}H) 196 (^{17}O)	833 (F2) 1229 (F1)	0.4	512	8192 (F2) 128 (F1)	0.0081920 (F2) 0.0006399 (F1)	200
2B benzoic	9.4 T 400.3 (^{1}H)	^{1}H spin echo (50 kHz)	29	500	150	8	8192	0.0204800	20
2C benzoic (960 µs)	9.4 T 400.3 (^{1}H) 54.3 (^{17}O)	2D ^{17}O$\rightarrow$^{1}H D-RINEPT (50 kHz MAS)	24 (^{1}H) 168 (^{17}O)	250 (F2) 921 (F1)	1	512	4096 (F2) 64 (F1)	0.0204800 (F2) 0.000640 (F1)	200
2C / S3A benzoic (320 µs)	9.4 T 400.3 (^{1}H) 54.3 (^{17}O)	2D ^{17}O$\rightarrow$^{1}H D-RINEPT (50 kHz MAS)	24 (^{1}H) 168 (^{17}O)	250 (F2) 921 (F1)	1	512	4096 (F2) 64 (F1)	0.0204800 (F2) 0.000640 (F1)	200
2D benzoic (960 µs)	9.4 T 400.3 (^{1}H) 28.9 (^{14}N)	2D ^{14}N$\rightarrow$^{1}H D-HMQC (50 kHz MAS)	29 (^{1}H) 692 (^{14}N)	500 (F2) 1728 (F1)	20	8	4096 (F2) 92 (F1)	0.0102400 (F2) 0.0009200 (F1)	50 (F2) 100 (F1)
2D / S4A benzoic (240 µs)	9.4 T 400.3 (^{1}H) 28.9 (^{14}N)	2D ^{14}N$\rightarrow$^{1}H D-HMQC (50 kHz MAS)	29 (^{1}H) 692 (^{14}N)	500 (F2) 1728 (F1)	20	8	4096 (F2) 92 (F1)	0.0102400 (F2) 0.0009200 (F1)	50 (F2) 100 (F1)
2E benzoic (left) (200 ms)	9.4 T 400.3 (^{1}H)	2D ^{1}H spin diffusion (50 kHz MAS)	24	500	10	4	4096 (F2) 64 (F1)	0.0102400 (F2) 0.0025600 (F1)	10
2E benzoic (right)	9.4 T 400.3 (^{1}H)	2D ^{1}H SQ/DQ (50 kHz MAS)	24	500	8	16	8192 (F2) 64 (F1)	0.0204800 (F2) 0.0012800 (F1)	10
3B oxalic	9.4 T 400.3 (^{1}H)	^{1}H spin echo (50 kHz MAS)	29	500	150	8	8192	0.0204800	10
3C oxalic (480 µs)	9.4 T 400.3 (^{1}H) 54.3 (^{17}O)	2D ^{17}O$\rightarrow$^{1}H D-RINEPT (50 kHz MAS)	24 (^{1}H) 168 (^{17}O)	250 (F2) 921 (F1)	0.15	128	4096 (F2) 64 (F1)	0.0204800 (F2) 0.000640 (F1)	200 (F2) 100 (F1)
3D oxalic (960 µs)	9.4 T 400.3 (^{1}H) 28.9 (^{14}N)	2D ^{14}N$\rightarrow$^{1}H D-HMQC (50 kHz MAS)	29 (^{1}H) 692 (^{14}N)	500 (F2) 1728 (F1)	20	8	4096 (F2) 92 (F1)	0.0102400 (F2) 0.0009200 (F1)	50 (F2) 100 (F1)
3D / S4B oxalic (240 µs)	9.4 T 400.3 (^{1}H) 28.9 (^{14}N)	2D ^{14}N$\rightarrow$^{1}H D-HMQC (50 kHz MAS)	29 (^{1}H) 692 (^{14}N)	500 (F2) 1728 (F1)	20	8	4096 (F2) 92 (F1)	0.0102400 (F2) 0.0009200 (F1)	50 (F2) 100 (F1)
4B maleic	9.4 T 400.3 (^{1}H)	^{1}H spin echo (50 kHz MAS)	29	500	150	8	8192	0.0204800	20
4C maleic (320 µs)	9.4 T 400.3 (^{1}H) 54.3 (^{17}O)	2D ^{17}O$\rightarrow$^{1}H D-RINEPT (50 kHz MAS)	24 (^{1}H) 168 (^{17}O)	250 (F2) 921 (F1)	1	512	4096 (F2) 64 (F1)	0.0204800 (F2) 0.000640 (F1)	200
4C / SS maleic	9.4 T 400.3 (^{1}H)	2D ^{17}O$\rightarrow$^{1}H D-RINEPT	24 (^{1}H) 168 (^{17}O)	250 (F2) 921 (F1)	1	512	4096 (F2)	0.0204800 (F2)	200

(960 µs)	54.3 (^{17}O)	(50 kHz MAS)					64 (F1)	0.000640<br>(F1)	
4D<br>maleic<br>(960 µs)	9.4 T<br>400.3 (^{1}H)<br>28.9 (^{14}N)	2D ^{14}N→^{1}H<br>D-HMQC<br>(50 kHz MAS)	29 (^{1}H)<br>692 (^{14}N)	500 (F2)<br>1728 (F1)	20	8	4096<br>(F2)<br>92 (F1)	0.0102400<br>(F2)<br>0.0009200<br>(F1)	50 (F2)<br>100 (F1)
4D / SS<br>maleic<br>(240 µs)	9.4 T<br>400.3 (^{1}H)<br>28.9 (^{14}N)	2D ^{14}N→^{1}H<br>D-HMQC<br>(50 kHz MAS)	29 (^{1}H)<br>692 (^{14}N)	500 (F2)<br>1728 (F1)	20	8	4096<br>(F2)<br>92 (F1)	0.0102400<br>(F2)<br>0.0009200<br>(F1)	50 (F2)<br>100 (F1)
4F<br>maleic<br>(top)<br>VT +45 ºC	25.8 T<br>149.2 (^{17}O)	^{17}O spin echo<br>(50 kHz MAS)	264	1675	2	2048	4096	0.0081920	200
4F<br>maleic<br>(2nd from top)<br>VT off	25.8 T<br>149.2 (^{17}O)	^{17}O CPMG<br>(50 kHz MAS)	17.2	1024	2	1340	8960	0.0224000	2
4F<br>maleic<br>(3rd from top)	14.1 T<br>81.4 (^{17}O)	^{17}O CPMG<br>(40 kHz MAS)	250	2457	1	6144	16600	0.0415000	5
4G<br>maleic<br>(1120 µs)<br>VT +45 ºC	25.8 T<br>1100.4 (^{1}H)<br>149.2 (^{17}O)	2D ^{17}O→^{1}H<br>D-RINEPT<br>(50 kHz MAS)	8.3 (^{1}H)<br>264 (^{17}O)	91 (F2)<br>335 (F1)	1.5	128	4000<br>(F2)<br>160 (F1)	0.020000<br>(F2)<br>0.0016000<br>(F1)	-20 (F2)<br>500 (F1)
4G / S3D<br>maleic<br>(320 µs)<br>VT +45 ºC	25.8 T<br>1100.4 (^{1}H)<br>149.2 (^{17}O)	2D ^{17}O→^{1}H<br>D-RINEPT<br>(50 kHz MAS)	14.1 (^{1}H)<br>264 (^{17}O)	91 (F2)<br>335 (F1)	1.25	64	4000<br>(F2)<br>232 (F1)	0.020000<br>(F2)<br>0.0023200<br>(F1)	-20 (F2)<br>500 (F1)
4G<br>maleic<br>(720 µs)<br>VT off	25.8 T<br>1100.4 (^{1}H)<br>79.5 (^{14}N)	2D ^{14}N→^{1}H<br>D-HMQC<br>(50 kHz MAS)	11.6 (^{1}H)<br>-75 (^{17}O)	91 (F2)<br>629 (F1)	10	8	4000<br>(F2)<br>128 (F1)	0.020000<br>(F2)<br>0.0012800<br>(F1)	-20 (F2)<br>500 (F1)
5B<br>malonic	9.4 T<br>400.3 (^{1}H)	^{1}H spin echo<br>(50 kHz MAS)	29	500	150	8	8192	0.0204800	20
5C<br>malonic<br>(960 µs)	9.4 T<br>400.3 (^{1}H)<br>54.3 (^{17}O)	2D ^{17}O→^{1}H<br>D-RINEPT<br>(50 kHz MAS)	24 (^{1}H)<br>168 (^{17}O)	250 (F2)<br>921 (F1)	1	512	4096<br>(F2)<br>64 (F1)	0.0204800<br>(F2)<br>0.000640<br>(F1)	200
5C<br>malonic<br>(960 µs)	9.4 T<br>400.3 (^{1}H)<br>54.3 (^{17}O)	2D ^{17}O→^{1}H<br>D-RINEPT<br>(50 kHz MAS)	24 (^{1}H)<br>168 (^{17}O)	250 (F2)<br>921 (F1)	1	512	4096<br>(F2)<br>64 (F1)	0.0204800<br>(F2)<br>0.000640<br>(F1)	200
5D<br>malonic<br>(960 µs)	9.4 T<br>400.3 (^{1}H)<br>28.9 (^{14}N)	2D ^{14}N→^{1}H<br>D-HMQC<br>(50 kHz MAS)	29 (^{1}H)<br>692 (^{14}N)	500 (F2)<br>1728 (F1)	20	8	4096<br>(F2)<br>92 (F1)	0.0102400<br>(F2)<br>0.0009200<br>(F1)	50 (F2)<br>100 (F1)
5D / SS<br>malonic<br>(240 µs)	9.4 T<br>400.3 (^{1}H)<br>28.9 (^{14}N)	2D ^{14}N→^{1}H<br>D-HMQC<br>(50 kHz MAS)	29 (^{1}H)<br>692 (^{14}N)	500 (F2)<br>1728 (F1)	20	8	4096<br>(F2)<br>92 (F1)	0.0102400<br>(F2)<br>0.0009200<br>(F1)	50 (F2)<br>100 (F1)
5F<br>malonic<br>(top)<br>VT +45 ºC	25.8 T<br>149.2 (^{17}O)	^{17}O spin echo<br>(50 kHz MAS)	264	1675	12	1600	4096	0.0081920	250
5F<br>malonic<br>(2nd from top)<br>VT off	25.8 T<br>149.2 (^{17}O)	^{17}O CPMG<br>(50 kHz MAS)	264	1340	2	1024	11520	0.028800	2
5F<br>malonic<br>(3rd from top)	14.1 T<br>81.4 (^{17}O)	^{17}O CPMG<br>(40 kHz MAS)	250	2457	1	6144	16600	0.041500	5
5F<br>malonic<br>(bottom)	9.4 T<br>54.3 (^{17}O)	^{17}O spin echo<br>(50 kHz MAS)	26	3685	0.2	16000	7200	0.0180000	200
5G<br>malonic<br>(480 µs)<br>VT +45 ºC	25.8 T<br>1100.4 (^{1}H)<br>149.2 (^{17}O)	2D ^{17}O→^{1}H<br>D-RINEPT<br>(50 kHz MAS)	12.2 (^{1}H)<br>264 (^{17}O)	91 (^{1}H)<br>335 (^{17}O)	1.5	16	4000<br>(F2)<br>232 (F1)	0.020000<br>(F2)<br>0.0023200<br>(F1)	-20 (F2)<br>800 (F1)
5H<br>malonic<br>(960 µs)<br>VT +45 ºC	25.8 T<br>1100.4 (^{1}H)<br>149.2 (^{17}O)	2D ^{17}O→^{1}H<br>D-RINEPT<br>(50 kHz MAS)	12.2 (^{1}H)<br>264 (^{17}O)	91 (^{1}H)<br>335 (^{17}O)	1.5	16	4000<br>(F2)<br>232 (F1)	0.020000<br>(F2)<br>0.0023200<br>(F1)	-20 (F2)<br>800 (F1)

5I malonic (100 ms) VT +45 ºC	25.8 T 149.2 (^{17}O)	2D ^{17}O EXSY (50 kHz MAS)	264 (^{17}O)	1675 (F2) 335 (F1)	1.25	64	4096 (F2) 192 (F1)	0.0081920 (F2) 0.0019200 (F1)	800
5J malonic (320 µs) VT +45 ºC	25.8 T 1100.4 (^{1}H) 149.2 (^{17}O)	2D ^{17}O EXSY-filtered ^{17}O→^{1}H D-RINEPT (50 kHz MAS)	8.3 (^{1}H) 264 (^{17}O)	91 (F2) 335 (F1)	1.6	176	4000 (F2) 160 (F1)	0.0200000 (F2) 0.0016000 (F1)	-10 (F2) 500 (F1)
S1B oxalic acid dihydrate (160 µs)	14.1 T 600.2 (^{1}H) 81.4 (^{17}O)	2D ^{17}O→^{1}H D-RINEPT (50 kHz MAS)	9.5 (^{1}H) 196 (^{17}O)	833 (F1) 1229 (F2)	0.4	192	8192 (F2) 128 (F1)	0.0081920 (F2) 0.0006399 (F1)	200
S1C oxalic acid dihydrate (1280 µs)	14.1 T 600.2 (^{1}H) 81.4 (^{17}O)	2D ^{17}O→^{1}H D-RINEPT (50 kHz MAS)	9.5 (^{1}H) 196 (^{17}O)	833 (F1) 1229 (F2)	0.4	512	8192 (F2) 128 (F1)	0.0081920 (F2) 0.0006399 (F1)	200
S1D oxalic acid dihydrate	14.1 T 81.4 (^{17}O)	^{17}O spin echo (50 kHz MAS)	196	2457	0.3	10240	2048	0.0051200	300
S3A benzoic (320 µs)	9.4 T 400.3 (^{1}H) 54.3 (^{17}O)	2D ^{17}O→^{1}H D-RINEPT (50 kHz MAS)	24 (^{1}H) 168 (^{17}O)	250	1	256	4096 (F2) 64 (F1)	0.0204800 (F2) 0.000640 (F1)	200
S3B maleic (960 µs)	9.4 T 400.3 (^{1}H) 54.3 (^{17}O)	2D ^{17}O→^{1}H D-RINEPT (50 kHz MAS)	24 (^{1}H) 168 (^{17}O)	250	1	512	4096 (F2) 64 (F1)	0.0204800 (F2) 0.000640 (F1)	200
S3C malonic (320 µs)	9.4 T 400.3 (^{1}H) 54.3 (^{17}O)	2D ^{17}O→^{1}H D-RINEPT (50 kHz MAS)	24 (^{1}H) 168 (^{17}O)	250	1	256	4096 (F2) 64 (F1)	0.0204800 (F2) 0.000640 (F1)	200
S3D maleic (320 µs) VT +45 ºC	25.8 T 1100.4 (^{1}H) 149.2 (^{17}O)	2D ^{17}O→^{1}H D-RINEPT (50 kHz MAS)	14.1 (^{1}H) 264 (^{17}O)	91 (F2) 335 (F1)	1.25	64	4000 (F2) 232 (F1)	0.020000 (F2) 0.0023200 (F1)	-20 (F2) 500 (F1)
S4A benzoic (240 µs)	9.4 T 400.3 (^{1}H) 28.9 (^{14}N)	2D ^{14}N→^{1}H D-HMQC (50 kHz MAS)	29 (^{1}H) 692 (^{14}N)	500 (F2) 1728 (F1)	30	8	4096 (F2) 92 (F1)	0.0102400 (F2) 0.0009200 (F1)	F2 (50) F1 (100)
S4B oxalic (240 µs)	9.4 T 400.3 (^{1}H) 28.9 (^{14}N)	2D ^{14}N→^{1}H D-HMQC (50 kHz MAS)	29 (^{1}H) 692 (^{14}N)	500 (F2) 1728 (F1)	30	8	4096 (F2) 92 (F1)	0.0102400 (F2) 0.0009200 (F1)	F2 (50) F1 (100)
S4C maleic ((240 µs)	9.4 T 400.3 (^{1}H) 28.9 (^{14}N)	2D ^{14}N→^{1}H D-HMQC (50 kHz MAS)	29 (^{1}H) 692 (^{14}N)	500 (F2) 1728 (F1)	30	8	4096 (F2) 92 (F1)	0.0102400 (F2) 0.0009200 (F1)	F2 (50) F1 (100)
S4D malonic (240 µs)	9.4 T 400.3 (^{1}H) 28.9 (^{14}N)	2D ^{14}N→^{1}H D-HMQC (50 kHz MAS)	29 (^{1}H) 692 (^{14}N)	500 (F2) 1728 (F1)	30	8	4096 (F2) 92 (F1)	0.0102400 (F2) 0.0009200 (F1)	F2 (50) F1 (100)
S4E malonic (240 µs) VT off	25.8 T 1100.4(^{1}H) (^{14}N)	2D ^{14}N→^{1}H D-HMQC (50 kHz MAS)	11.6 (^{1}H) -75.5 (^{14}N)	91 (F2) 629 (F1)	10	8	4000 (F2) 128 (F1)	0.0200000 (F2) 0.0012800 (F1)	800
S4F malonic (720 µs) VT off	25.8 T 1100.4(^{1}H) (^{14}N)	2D ^{14}N→^{1}H D-HMQC (50 kHz MAS)	11.6 (^{1}H) -75.5 (^{14}N)	91 (F2) 629 (F1)	10	8	4000 (F2) 128 (F1)	0.0200000 (F2) 0.0012800 (F1)	800
S6A oxalic (200 ms)	9.4 T 400.3 (^{1}H)	2D ^{1}H-^{1}H spin diffusion (50 kHz MAS)	23.507	500	8	16	8192	0.0204800	10
S6B maleic (200 ms)	9.4 T 400.3 (^{1}H)	2D ^{1}H-^{1}H spin diffusion (50 kHz MAS)	23.507	500	8	16	8192	0.0204800	10
S6C malonic (200 ms)	9.4 T 400.3 (^{1}H)	2D ^{1}H-^{1}H spin diffusion (50 kHz MAS)	23.507	500	8	16	8192	0.0204800	10
S7A oxalic	9.4 T 400.3 (^{1}H)	2D ^{1}H-^{1}H SQ/DQ (50 kHz MAS)	23.507	500	8	16	8192	0.0204800	10

S7B maleic	9.4 T 400.3 (^{1}H)	2D ^{1}H-^{1}H SQ/DQ (50 kHz MAS)	23.507	500	8	16	8192	0.0204800	10
S7C malonic	9.4 T 400.3 (^{1}H)	2D ^{1}H-^{1}H SQ/DQ (50 kHz MAS)	23.507	500	8	16	8192	0.0204800	10
S7D malonic	25.8 T 1100.4(^{1}H)	2D ^{1}H-^{1}H SQ/DQ (50 kHz MAS)	23.507	500	8	16	8192	0.0204800	10
S8 maleic (top) VT +45 ºC	25.8 T 149.2 (^{17}O)	^{17}O spin echo (50 kHz MAS)	264	1675	2	2048	4096	0.0081920	200
S8 maleic (middle) VT off	25.8 T 149.2 (^{17}O)	^{17}O spin echo (50 kHz MAS)	264	1675	2	512	4096	0.0081920	200
S8 maleic (bottom) VT –5.4 ºC	25.8 T 149.2 (^{17}O)	^{17}O spin echo (50 kHz MAS)	264	1675	2	512	4096	0.0081920	200
S9 malonic (top) VT +45 ºC	25.8 T 149.2 (^{17}O)	^{17}O spin echo (50 kHz MAS)	264	1675	12	1600	4096	0.0081920	250
S9 malonic (2nd from top) VT +45 ºC	25.8 T 149.2 (^{17}O)	^{17}O spin echo (50 kHz MAS)	264	1675	2	1024	4096	0.0081920	200
S9 malonic (3rd from top) VT off	25.8 T 149.2 (^{17}O)	^{17}O spin echo (50 kHz MAS)	264	1675	8	2048	4096	0.0081920	200
S9 malonic (bottom) VT –10.5 ºC	25.8 T 149.2 (^{17}O)	^{17}O spin echo (50 kHz MAS)	264	1675	2	1024	4096	0.0081920	200
S10A (top) benzoic	14.1 T 81.4 (^{17}O)	^{17}O QCPMG (30 kHz MAS)	250	2457	1	55296	16600	0.0415	5
S10A (middle) benzoic	9.4 T 400.3 (^{1}H) 28.9 (^{14}N)	2D ^{14}N→^{1}H D-HMQC (50 kHz MAS)	29 (^{1}H) 692 (^{14}N)	500 (F2) 1728 (F1)	30	8	4096 (F2) 92 (F1)	0.0102400 (F2) 0.0009200 (F1)	50 (F2) 100 (F1)
S10B (top) oxalic	14.1 T 81.4 (^{17}O)	^{17}O QCPMG (50 kHz MAS)	196	2457	60	512	17000	0.0415	1
S10B (middle) oxalic	9.4 T 400.3 (^{1}H) 28.9 (^{14}N)	2D ^{14}N→^{1}H D-HMQC (50 kHz MAS)	29 (^{1}H) 692 (^{14}N)	500 (F2) 1728 (F1)	30	8	4096 (F2) 92 (F1)	0.0102400 (F2) 0.0009200 (F1)	50 (F2) 100 (F1)
S10B (bottom) oxalic	9.4 T 400.3 (^{1}H) 28.9 (^{14}N)	2D ^{14}N→^{1}H D-HMQC (50 kHz MAS)	29 (^{1}H) 692 (^{14}N)	500 (F2) 1728 (F1)	20	8	4096 (F2) 92 (F1)	0.0102400 (F2) 0.0009200 (F1)	F2 (50) F1 (100)
S10C (top) maleic	14.1 T 81.4 (^{17}O)	^{17}O QCPMG (40 kHz MAS)	250	2457	1	6144	16600	0.0415	5
S10C (middle) maleic	9.4 T 400.3 (^{1}H) 28.9 (^{14}N)	2D ^{14}N→^{1}H D-HMQC (50 kHz MAS)	29 (^{1}H) 692 (^{14}N)	500 (F2) 1728 (F1)	30	8	4096 (F2) 92 (F1)	0.0102400 (F2) 0.0009200 (F1)	F2 (50) F1 (100)
S10C (bottom) maleic	9.4 T 400.3 (^{1}H) 28.9 (^{14}N)	2D ^{14}N→^{1}H D-HMQC (50 kHz MAS)	29 (^{1}H) 692 (^{14}N)	500 (F2) 1728 (F1)	20	8	4096 (F2) 92 (F1)	0.0102400 (F2) 0.0009200 (F1)	F2 (50) F1 (100)
S10D (top) malonic	14.1 T 81.4 (^{17}O)	^{17}O QCPMG (40 kHz MAS)	250	2457	1	6144	16600	0.0415	5
S10D (middle) malonic	9.4 T 400.3 (^{1}H) 28.9 (^{14}N)	2D ^{14}N→^{1}H D-HMQC (50 kHz MAS)	29 (^{1}H) 692 (^{14}N)	500 (F2) 1728 (F1)	30	8	4096 (F2) 92 (F1)	0.0102400 (F2) 0.0009200 (F1)	F2 (50) F1 (100)

S10D (bottom) malonic	9.4 T 400.3 (^{1}H) 28.9 (^{14}N)	2D ^{14}N→^{1}H D-HMQC (50 kHz MAS)	29 (^{1}H) 692 (^{14}N)	500 (F2) 1728 (F1)	20	8	4096 (F2) 92 (F1)	0.0102400 (F2) 0.0009200 (F1)	F2 (50) F1 (100)

Supplementary Information References:

(1) Hagaman E. W.; Chen, B.; Jiao, J.; Parsons, W. Solid-state ^{17}O NMR study of benzoic acid adsorption on metal oxide surfaces. *Solid State Nucl. Magn. Reson*. **2012**, *41*, 60–67.

(2) Trask, A. V.; Motherwell, W. D. S.; Jones, W. Physical stability enhancement of theophylline via cocrystallization. *Int. J. Pharm.* **2006**, *320*, 114-123. (CSD XEJWUF, CSD XEJXEQ)

(3) Huang, Y.; Zhou, L.; Yang, W.; Li, Y.; Yang, Y.; Zhang, Z.; Wang, C.; Zhang, X.; Yun, Q. Preparation of Theophylline-Benzoic Acid Cocrystal and On-Line Monitoring of Cocrystallization Process in Solution by Raman Spectroscopy. *Crystals* **2019**, *9*, 329.

(4) Sabine, T. M.; Cox, G. W.; Craven, B. M. A Neutron Diffraction Study of a-Oxalic Dihydrate. *Acta Crystallographica, Section B: Struct. Crystallogr. Cryst. Chem.*, **1969**, *25*, 2437-2441. (CSD OXACDH04)

(5) Bruker TopSpin Software, Version 3.7; Bruker BioSpin GmbH: Rheinstetten, Germany.

(6) Macrae, C. F.; Sovago, I.; Cottrell, S. J.; Galerk, P. T. A.; McCabe, P.; Pidcock, E.; Platings, M.; Shields, G. P.; Stevens, J. S.; Towler, M.; Wood, P. A. J. AppMercury 4.0: from visualization to analysis, design and prediction. *J. Appl. Cryst*., **2020**, *53*, 226–235.

(7) Groom, C. R.; Bruno, I. J.; Lightfoot, M. P.; Ward. S. C. The Cambridge Structural Database. *Acta Cryst*. **2016**, *B72*, 171–179.

(8) Ebisuzaki, Y.; Boyle, P. D.; Smith, J. A. Methylxanthines. I. Anhydrous theophylline. *Acta Crystallogr. C Struct. Chem.*, **1997**, 53, 777-779. (CSD BAPLOT01)

(9) Corpinot, M. K.; Stratford, S. A.; Arhangelskis, M.; Anka-Lufford, J.; Halasz, I.; Judaš, N.; Jones, W.; Bučar, D. On the predictability of supramolecular interactions in molecular cocrystals – the view from the bench. *CrystEngComm*, **2016**, *18*, 5434-5439. (CSD VAXSAQ01)

(10) Derissen, J. L.; Smit, P. H. Refinement of the Crystal Structures of Anhydrous α- and β-Oxalic Acids. *Acta. Crystallogr. B. Struct. Sci. Cryst. Eng. Mater.* **1974**, *30*, 2240-2242. (CSD OXALAC03, CSD OXALAC04)

(11) Macchietti, L.; Casali, L.; Emmerling, F.; Braga, D.; Grepioni, F. Deriving kinetic insights from mechanochemically synthesized compounds using multivariate analysis (MCR-ALS) of powder X-ray diffraction data. *RSC. Mechanochem*. **2024**, *1*, 106-115. (CSD XEJXAM03)

(12) Hoffman, R. Solid-state chemical-shift referencing with adamantane. *J. Magn. Reson*. **2022**, *340*, 107231.

(13) Carnahan, S. L.; Lampkin, B. J.; Naik, P.; Hanrahan, M. P.; Slowing, I. I.; VanVeller, B.; Wu, G.; Rossini, A. J. Probing O−H Bonding through Proton Detected $^{1}H-^{17}O$ Double Resonance Solid-State NMR Spectroscopy. *J. Am. Chem. Soc*. **2019**, *141*, 441−450.

(14) Dorn, R. W.; Ryan, M. J.; Kim, T.-H.; Goh, T. W.; Venkatesh, A.; Heintz, P. M.; Zhou, L.; Huang, W.; Rossini, A. J. Identifying the Molecular Edge Termination of Exfoliated Hexagonal Boron Nitride Nanosheets with Solid-State NMR Spectroscopy and Plane-Wave DFT Calculations. *Chem. Mater.* **2020**, *32*, 3109–3121.

(15) Stevens, J. S.; Byard, S. J.; Seaton, C. C.; Sadiq, G.; Davey, R. J.; Schroeder, S. L. M. Proton transfer and hydrogen bonding in the organic solid state: a combined XRD/XPS/ssNMR study of 17 organic acid-base complexes. *Phys. Chem. Chem.* Phys. **2014**, *16*, 1150-1160.

(16) Pindelska, E.; Sokal, A.; Szeleszczuk, L.; Pisklak, D. M.; Kolodziejski, W. Solid-state NMR studies of theophylline co-crystals with dicarboxylic acids. *J. Pharm. Biomed. Anal.* **2014**, *100*, 322–328.
